# The Pnictogen Bond, Together with Other Non-Covalent Interactions, in the Rational Design of One-, Two- and Three-Dimensional Organic-Inorganic Hybrid Metal Halide Perovskite Semiconducting Materials, and Beyond

**DOI:** 10.3390/ijms23158816

**Published:** 2022-08-08

**Authors:** Arpita Varadwaj, Pradeep R. Varadwaj, Helder M. Marques, Koichi Yamashita

**Affiliations:** 1 Department of Chemical System Engineering, School of Engineering, The University of Tokyo, 7-3-1, Tokyo 113-8656, Japan; 2 Molecular Sciences Institute, School of Chemistry, University of the Witwatersrand, Johannesburg 2050, South Africa

**Keywords:** pnictogen bonding, nitrogen as pnictogen bond donor, intermolecular geometries and directionality, inorganic-organic hybrid halide perovskites, ICSD and CSD database analyses, MESP characterizations, sum of the van der Waals radii concept, IGM-δ*g* analysis

## Abstract

The pnictogen bond, a somewhat overlooked supramolecular chemical synthon known since the middle of the last century, is one of the promising types of non-covalent interactions yet to be fully understood by recognizing and exploiting its properties for the rational design of novel functional materials. Its bonding modes, energy profiles, vibrational structures and charge density topologies, among others, have yet to be comprehensively delineated, both theoretically and experimentally. In this overview, attention is largely centered on the nature of nitrogen-centered pnictogen bonds found in organic-inorganic hybrid metal halide perovskites and closely related structures deposited in the Cambridge Structural Database (CSD) and the Inorganic Chemistry Structural Database (ICSD). Focusing on well-characterized structures, it is shown that it is not merely charge-assisted hydrogen bonds that stabilize the inorganic frameworks, as widely assumed and well-documented, but simultaneously nitrogen-centered pnictogen bonding, and, depending on the atomic constituents of the organic cation, other non-covalent interactions such as halogen bonding and/or tetrel bonding, are also contributors to the stabilizing of a variety of materials in the solid state. We have shown that competition between pnictogen bonding and other interactions plays an important role in determining the tilting of the MX_6_ (X = a halogen) octahedra of metal halide perovskites in one, two and three-dimensions. The pnictogen interactions are identified to be directional even in zero-dimensional crystals, a structural feature in many engineered ordered materials; hence an interplay between them and other non-covalent interactions drives the structure and the functional properties of perovskite materials and enabling their application in, for example, photovoltaics and optoelectronics. We have demonstrated that nitrogen in ammonium and its derivatives in many chemical systems acts as a pnictogen bond donor and contributes to conferring stability, and hence functionality, to crystalline perovskite systems. The significance of these non-covalent interactions should not be overlooked, especially when the focus is centered on the rationale design and discovery of such highly-valued materials.

## 1. Introduction

**Scheme 1:**
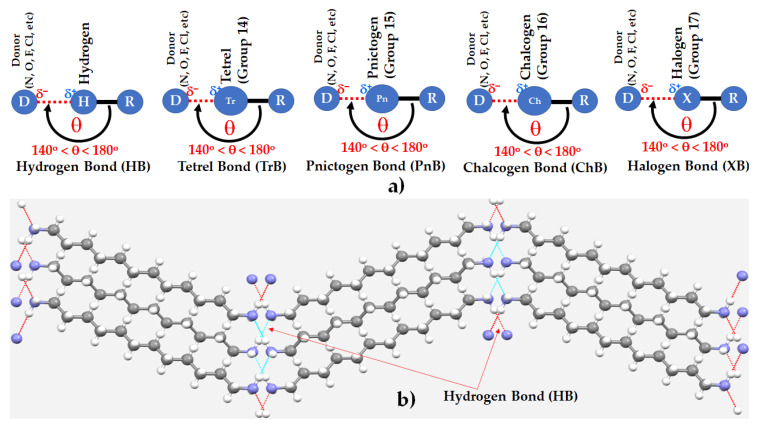
(**a**) Schematic representation of five different types of Type-II (140° < θ < 180°) non-covalent interactions formed by covalently bonded hydrogen, tetrel, pnictogen, chalcogen, and halogen atoms in molecular entities, where R is the remainder part of the molecular entity and θ is the angular approach of the electrophile centered on the H/Tr/Ch/Pn/X atoms. (**b**) A ball-and-stick model of dodecane-1,12-diamine (H_2_N-(CH_2_)_12_-NH_2_), in which the ammine moiety acts both as a hydrogen bonded acceptor and a hydrogen bond donor (CSD ref: UJONUD [11]). The H···N hydrogen bonds in b) are depicted as dotted lines in green and red, with the latter are hanging contacts. Color codes: N—blue; C—gray; H—white.

Atomic nitrogen, N, the first member of the pnictogen family, is one of the major components of Earth’s atmosphere. Its molecular analogue, N_2_, is a crucial constituent in feedstocks leading to the production of fertilizers and mining explosives, and high-density materials [1,2,3,4,5]. It is likely to play an ever more important role in the economy of the future as the era of the ammonia economy and “green ammonia” approaches [4,6,7,8,9]. Nitrogen in numerous molecules has been recognized widely as a Lewis base, and thus can serve as an electron density donor D for the formation of various types of intermolecular interactions, including hydrogen bonds (HBs), tetrel bonds (TrBs), pnictogen bonds (PnBs), chalcogen bonds (ChBs) and halogen bonds (XBs), among others (Scheme 1a).

In the crystalline phase, N_2_ molecules bond to each other through π···N(lone-pair) interactions, forming a variety of polymorphs referred to as the *α*-, *β*-, *γ*-, δ-, and *ε*-phases. The occurrence of these non-covalent interactions is unsurprising, given that the bonding region in N_2_ carries a positive potential, and the electron density accumulates along and around the extension of the N≡N triple bond [10].

N forms single, double and triple bonds, and, given its significant electronegativity (*χ*_Pauling_ = 3.04; *χ*_Allen_ = 3.066), often carries a negative charge and its ability to act as a Lewis base is well known. Ammines can act both as hydrogen bond donors and acceptors. Scheme 1b, for instance, shows the way the nitrogen in dodecane-1,12-diamine bonds non-covalently with its neighbors through hydrogen bonds (*r*(H···N) = 2.233 Å, ∠N–H···N = 170.9°), resulting in the formation of a crystal lattice [11]. N in NH_3_ and ammines is a ligand in thousands of transition metal complexes, and the coordination of NH_3_ and its derivatives by metal ions has been extensively studied for many years, and often used to explore trends in stability constants of the transition metal ions (for example [12,13,14,15,16,17,18,19,20,21]).

**Scheme 2:**
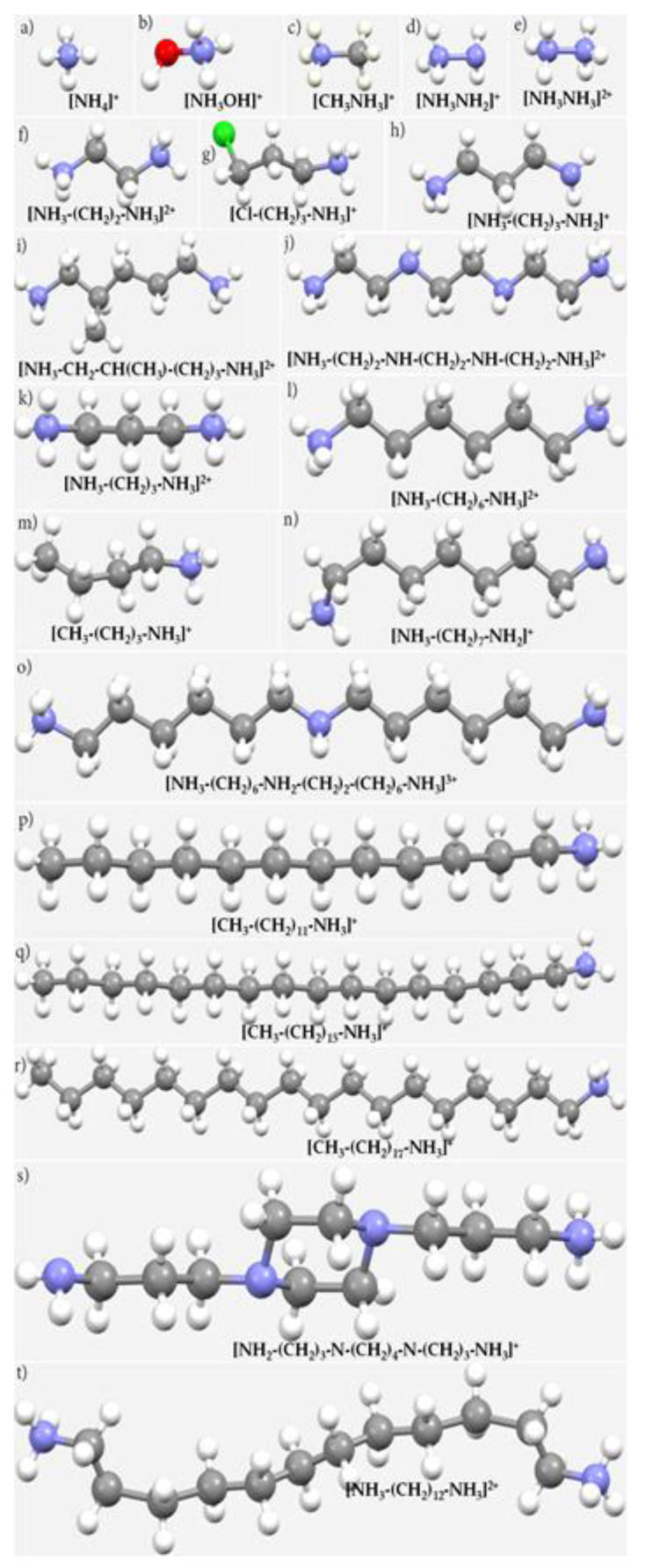
Some examples of ammines, diammines, and their derivatives for in crystal structures in the CSD. These include: (**a**) ammonium; (**b**) hydroxylammonium; (**c**) methylammonium, (**d**) hydrazinium; (**e**) diazanediium; (**f**) ethylenediammonium; (**g**) 3-chloropropan-1-aminium; (**h**) 3-aminopropan-1-aminium; (**i**) 2-methylpentane-1,5-bis(ammonium); (**j**) *N,N’*-bis(2-ammonioethyl)ethylene-1,2-diamine; (**k**) propane-1,3-diaminium; (**l**) 1,6-hexanediammonium; (**m**) n-butylammonium; (**n**) 7-aminoheptylazanium; (**o**) bis(hexamethylene)triammonium; (**p**) dodecan-1-aminium; (**q**) n-hexadecylammonium; (**r**) octadecan-1-aminium; (**s**) 3-(4-(3-aminopropyl)piperazin-1-yl)propan-1-aminium; (**t**) dodecane-1,12-diammonium.

There are many ammonium, diammonium and their derivatives found in solid state structures, including those that may be suitable in the development of photovoltaic and optoelectronic materials (Scheme 2). The presence of cations such as these, often referred to as *spacers*, or *additives*, stabilizes the inorganic frameworks, and hence plays a structure-determining role during the synthesis of organic-inorganic hybrid materials and is responsible for their functionality. Lower-dimensional organic-inorganic hybrid metal trihalide perovskite semiconductors are prominent examples. In these, bifunctional organoammonium cations X(CH_2_)_2_NH_3_^+^ (X = OH, Cl, Br, I, CN) control the in- and out-of-plane distortions of X(CH_2_)_2_NH_3_)_2_PbI_4_ perovskites [22]. The family of ⟨100⟩-oriented layered perovskites with general formula (RNH_3_)_2_A*_n–_*_1_B*_n_*X_3*n*+1_ are obtained by taking *n* layers along the ⟨100⟩ direction of the parent structure [22]. A search for the fragment “–CH_2_–CH_2_–CH_2_–NH_3_” in the Cambridge Structural Database (CSD [23,24]) led to 3656 hits; that for the fragment “–X–C–NH_3_” or “X–CH_2_–NH_3_” (X = any atom) led to 16,834 and 9509 hits, respectively, illustrating the wide occurrence of structures featuring an RNH_3_^+^ entity. Although a few studies have discussed the importance of pnictogen bonding in the metal trihalide perovskite semiconductors (for example [25,26]), in addition to other non-covalent interactions such as hydrogen bonding and tetrel bonding [27,28,29], our exploration of the geometry of a variety of crystal structures suggests that the occurrence of nitrogen-centered pnictogen bonding in these systems is very common and that it plays an important role in their structural integrity.

This overview is therefore focused on exploring representative crystal structures deposited in the CSD and the Inorganic Chemistry Structural Database (ICSD) [30,31] in which covalently bound positively-charged nitrogen contributes in part to their structural stability through inter- or intra-molecular non-covalent interactions. In many cases, we attribute the attraction between N and the negative site as a charge-assisted nitrogen-centered pnictogen bond (or simply, nitrogen bond [10]). This could be either a σ-hole centered or a π-hole centered nitrogen bonding interaction. A σ-hole on an atom A is recognized along the extension of the R–A covalent bond, and is deficient in electron density, where R is the remainder part of the molecular entity [32,33]. A π-hole can be recognized on an atom (for example, N in NO_3_^−^), or on a molecular fragment (for example, the central portion of the C≡C bond in acetylene), or at the center of a delocalized system (such as an arene moiety) that has the ability to accept electron density from a lone-pair on a Lewis base (such as O in H_2_O and N in NH_3_). Examples of this have been discussed elsewhere [10,34,35,36]. Our survey included only single crystals in the CSD and ICSD that were free of errors and distortions, and that had an *R*-factor ≤ 0.1. A statistical analysis was performed using the geometric data obtained from this survey, which is presented before the conclusions section.

The nitrogen bond, or a covalently bound nitrogen-centered pnictogen bond, in chemical systems occurs when there is evidence of a net attractive interaction between the electrophilic region associated with a covalently or coordinately bound nitrogen atom in a molecular entity and a nucleophile in another, or the same, molecular entity. It is the first member of the family of pnictogen bonds formed by the first atom of the pnictogen family, Group 15, of the periodic table, and is an inter- or intra-molecular non-covalent interaction [10]. The possible occurrence of pnictogen bonds in many crystal lattices, formed by covalently bound nitrogen [10,33], phosphorous [33,35], arsenic [33,34], antimony [36] and bismuth [37], has already been discussed recently. 

Four specific features were considered when identifying nitrogen bonding in the illustrative crystal systems chosen in this overview: (1) application of the concept of “less than the sum of the van der Waals (vdW) radii” [38,39,40,41]; (2) the directionality of the putative interaction [42,43]; (3) the positive nature of the electrostatic potential on the surface of N in molecular entities and its engagement with a negative site [10]; and (4) the existence of promolecular charge density-based isosurface volumes between the bonded atomic basins [44,45]. While our discussion is largely focused on the utilization of 1 and 2 in identifying pnictogen bonding in the illustrative crystal systems, the latter two properties were computed for some chosen systems to confirm the occurrence of such interactions between molecular entities that play any appreciable role in the overall stability of the crystal lattice, and hence in the functionality of these materials. Application of the four features above has been informative in rationalizing inter- and intra-molecular interactions of various kinds [10,34,35,36,38,42] in a variety of chemical systems, and hence further demonstration is unnecessary. We note that the appearance of N-centered pnictogen bonding in the illustrative crystals is usually accompanied by hydrogen bonds. In the majority of the crystals explored, we found that these are stronger than the nitrogen bonds; it is therefore reasonable to conclude that they are usually the cause, and that the emergence of nitrogen bonding is probably an effect.

This overview is structured as follows. Following the Computational Details section, we provide examples of illustrative crystal systems featuring the wide occurrence of N-centered pnictogen bonds. This section comprises subsections that highlight 0D, 1D, 2D and 3D crystals, the dimension of the inorganic framework in the organic-organic frameworks. Prior to the Conclusion section, we include a section that shows the frequency of the various N-center pnictogen binding interactions that occur in crystals. Electron density donors of different types are considered in this section.

## 2. Computational Details

Ammonium and its derivatives (see Scheme 2) are entirely positive. This means that both the H and N atoms in NH_4_^+^, as well as the –CH_2_– and –CH_3_ fragments in the derivatives of mono-, di-, tri- and multi-ammonium molecular entities are all positive. Depending on the nature of the (R–CH_2_–)_n_ fragment in [R–(CH_2_)_n_–NH_3_]^n+^ (R = H, X, NH_3_ and CH_3_, etc.), the potential on the electrostatic surface of each constituent species differs from other species. Therefore, it is not necessary to compute the electrostatic potential on the surface of the organic cation contributing to the structural integrity of the organic-inorganic crystal lattices that are shown in the following section. Even so, we have chosen a few organic cations to show that the electrostatic potential on their surfaces differs from one atomic domain to the another that constitute the molecular cation.

Ab initio calculations at the MP2(FC) level theory [46,47] were performed on a selected number of organic cations in combination with Dunning’s Aug-CC-pVTZ basis set system [48,49] to obtain their equilibrium geometries; FC refers to the frozen core approximation. Frequency calculations were also performed for each of them, and positive eigenvalues were found. Using fully optimized geometries, we used the resulting wavefunctions to calculate the electrostatic surface potential of the molecular entities. The Gaussian 16 suit of programs was used [50]. The 0.001 *a.u. (electrons* Bohr^−3^) isoelectron density envelope that arbitrarily defines the van der Waals surface of a molecular entity was used on which to calculate the potential. 

The details of the Molecular Electrostatic Surface Potential (MESP) have been exhaustively described [32,33,38,51,52,53,54,55] and will therefore not be repeated here. In essence, the MESP model features two extrema, *V_S_*_,*min*_ and *V_S_*_,*max*_, called the local most minimum and the local most maximum of potential, respectively, that can be found on the electrostatic surface of a molecular entity. The sign and magnitude enable us to gain insight into the electrophilic and nucleophilic character and strength of specific regions on the surface of a molecular entity, respectively [54,55]. In particular, when *V_S_*_,*min*_ or *V_S_*_,*max*_ is positive (i.e., *V_S_*_,*min*_ > 0 or *V_S_*_,*max*_ > 0), they each represent regions that are electrophilic [10,34,35,36,42]. Similarly, when both are negative (i.e., *V_S_*_,*min*_ < 0 or *V_S_*_,*max*_ < 0), they represent regions that are nucleophilic. When a positive *V_S_*_,*max*_ appears along the outer extension of an R–A covalent or coordinate bond, it is called a σ-hole (as on C–X bond extensions in C_6_X_6_ (X = F, Cl, Br, I)) [56,57,58,59]. A σ-hole on atom A can be positive, negative or neutral, and is an electron density deficient region, along the extension of an R–A covalent, or coordinate bond, that is more positive relative to the lateral portions of A [32,55]. However, when a *V_S_*_,*min*_ > 0 is found on an R–A covalent, or coordinate, bond extension, it may represent an electron deficient region that could be positive [34]; it would not be regarded as a π-hole since its location is not perpendicular to the bond axis. A *V_S_*_,*min*_ > 0 occurs, for instance, along the extension of the bond in a P≡P molecule, as well as in an As_2_, Sb_2_ or Bi_2_ molecule [34,35,36]. When a positive region with *V_S_*_,*min*_ < 0 or *V_S_*_,*max*_ < 0 is close to a region described by *V_S_*_,*min*_ > 0 or *V_S_*_,*max*_ > 0, the formation of a non-covalent interaction is likely to occur. There have been instances found in which interacting regions with negative *V_S_*_,*max*_ [60,61] (or positive *V_S_*_,*max*_ [62]) along bond extensions can engage in forming non-covalent interactions.

A π-hole is an electron density deficient region, which is generally associated with a region on the surface of a molecular entity that lies orthogonal to the bond axis (such as on the centroid region of a C_6_H_6_ or C_6_X_6_ molecule). 

In this overview, we are interested in the characterization of pnictogen bonds between interacting entities in some illustrative crystals. To this end, we selected monomeric entities in several crystals. We analyzed the promolecular charge density-based isosurface calculated by the Independent Gradient Model, IGM-δ*g* promolecular framework [44,45] to strengthen our identification of chemical bonded interactions as revealed using intermolecular geometry and the conceptual framework of the MESP model as described above. IGM-δ*g* has already been shown in several studies [10,35,36,42] to be useful in providing an insightful, local description of the source of the atomic or fragmentary domain that is involved in an interaction. Within the framework of IGM, the atomic electron densities of pro-molecules are summed, and the associated atomic gradients do not interfere. This is achieved by using absolute values for the sum of the atomic gradients and eliminating any electron gradient contragradience feature. The resulting total gradient |∇*ρ^I^*^GM^| is the upper limit of the true gradient, and the difference δ*g* quantifies the collapse of the net electron density gradient due to the interaction. In other words, the δ*g* descriptor identifies the existence of the opposite sign in the components of the total electron density gradient |∇*ρ*(r)| due to the interaction. IGM automatically separates intramolecular and inter-fragment interactions in a molecular entity, which can be plotted in two or three dimensions (isosurface volumes) to determine the presence of intramolecular or intramolecular interactions. From the shape of the isosurface volume, we can infer the localized or delocalized nature of the interactions. The colors of these volumes, blue and green, represent strong and weak attraction, respectively, and red represents a repulsive interaction. 

To clarify the nature of the interaction energy between the interacting units responsible for the crystal geometry, and which are the building blocks in the crystals, we have used two theoretical methods, MP2(FC) and *ω*B97X-D [63], in conjunction with the def2-TZVPPD pseudopotential basis set [64], to optimize the geometries of the ion-pairs [NH_3_NH_3_][Cl]_2_, [CH_3_NH_3_][I], [NH_4_][Cl], [NH_4_][Br] and [NH_4_][I]. The uncorrected and basis set superposition error (BSSE) corrected (complexation) interaction energies, *E_i_*_nt_ and *E*_int_(BSSE), of these ion-pairs were calculated using Equations (1) and (2), respectively. *E*_T_(ion-pair), *E*_T_(ion1), *E*_T_(ion2) and *E*(BSSE) are the electronic total energies of the ion-pair, ion1, ion2 and energy due to BSSE, respectively.

*E*_int_ = *E*_T_(ion-pair) − *E*_T_(ion1) − *E*_T_(ion2) (1)

*E*_int_(BSSE) = *E*_int_ + *E*(BSSE) (2)

The quantum theory of atoms in molecules (QTAIM) has been widely used to provide insight into the chemical interactions present in molecules, molecular complexes and crystals [65,66,67]. It relies on the zero-flux boundary condition for space partitioning of atomic basins in molecular entities. The bond path topology is the graphical representation of atomic domains bonded to each other. Properties such as the charge density (*ρ*_b_), the sign and magnitude of the Laplacian of the charge density (∇^2^*ρ*_b_), the total energy density (*H*_b_) the bond critical points have been shown to be valid indicators in the process of recognition of inter- and intra-molecular interactions. ∇^2^*ρ*_b_ < 0 or ∇^2^*ρ*_b_ > 0 gives an indication of whether an interaction between atomic basins has an open-shell (region with charge concentration) or a closed-shell (region with charge depletion) character [65], although ∇^2^*ρ*_b_ > 0 has been identified for polar covalent bonds [68]. We have applied QTAIM to a few cases such as those mentioned above to clarify the assumed (misleading) perception that pnictogen bonding in the building blocks responsible for crystals is negligible and hence they do not make a significant contribution to the overall stability. Since the interaction energy of ion-pairs is electrostatically dominant, we used the empirical relationship, *E*_b_ = ½*V*_b_, to calculate the energy of the pnictogen bond in the simplified ion-pairs used for illustrative purposes [69], where *V*_b_ is the potential energy density at the bond critical point (bcp); this relationship has been utilized in many studies to calculate the interaction energy of hydrogen bonds in chemical systems (for example [70,71]). 

Natural Bond Orbital (NBO)’s Second Order Perturbation Theory Analysis of the Fock Matrix in NBO Basis [72,73,74,75], which is valid within the Restricted Hartree-Fock (RHF) approximation, provides insight into the type of charge transfer delocalization energy between the donor NBO (*i*) and the acceptor NBO (*j*). We have used a few systems to estimate the second order stabilization energy *E*^(2)^ associated with delocalization *i* → *j* given by Equation (3).

*E*^(2)^ = *q_i_* [*F*^2^(*i,j*)/(*ε_j_* − *ε_i_*)] (3)

In Equation (3), *q_i_* is the donor orbital occupancy, *ε_i_* and *ε_j_* are diagonal elements (orbital energies) and *F*(*i,j*) is the off-diagonal NBO Fock matrix element. For this purpose, the Restricted Hartree–Fock (RHF) method in conjunction with the split valence 6-311G* basis set was used. The complexation energy, QTAIM-based interaction energy and second order charge transfer energy were evaluated on the MP2(FC), *ω*B97X-D and crystal geometries of the chosen ion-pairs to demonstrate the persistency of results and conclusions achieved. 

Analysis and drawing of geometries of various molecular entities and crystals were performed using the Mercury 4.0 [76] and VMD [77] suite of programs. AIMAll [78] and MultiWfn [79] codes were used for calculation and analysis of MESP and QTAIM graphs, and VMD [77] was used for drawing of IGM-δ*g* based isosurfaces. 

## 3. Illustrative Crystal Systems

In what follows, we examine the bonding in representative simple ammonium and dihydrazinium (diazanediium) crystal structures, and in organic-inorganic hybrid metal halide perovskites [80]. 

### 3.1. Diazanediium Dichloride

**Figure 1:**
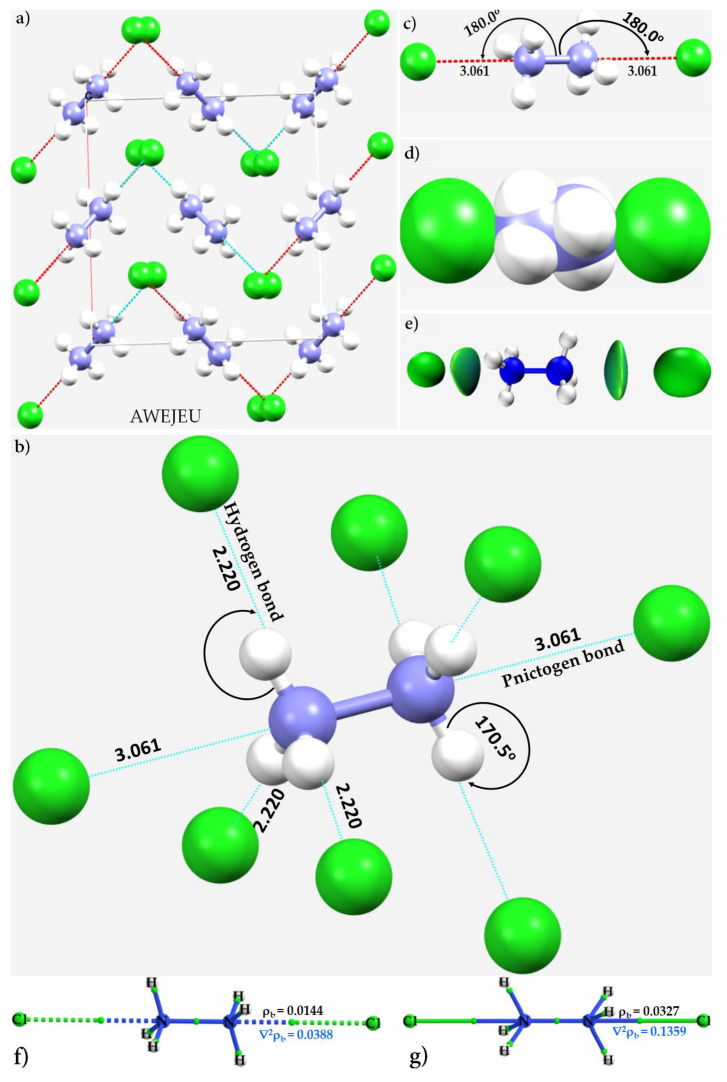
(**a**) The ball-and-stick model of the unit-cell of crystalline diazanediium dichloride, [NH_3_NH_3_][Cl]_2_; (**b**) Illustration of N···Cl and N–H···Cl pnictogen and hydrogen bonds between a single NH_3_NH_3_^2+^ dication and surrounding nearest-neighbor Cl^−^ ions in the [NH_3_NH_3_][Cl]_2_ crystal. (**c**) The local geometry of the interaction between two anions and a cation; (**d**) Space-filling model showing overlapping between N and Cl atomic basins in the geometry depicted in (**c**); and (**e**) IGM-δ*g* based isosurface volume (colored bluish-green) between N and Cl atomic basins between [NH_3_NH_3_]^2+^ and 2Cl^−^. (**f**,**g**) QTAIM-based molecular graphs for the [NH_3_NH_3_][Cl]_2_ ion-pair, obtained using the extracted geometry of the same ion-pair in the crystal (CSD ref: AWEJEU [81]) and the MP2(fc)/def2-TZVPPD optimized geometry, respectively. Values (in *a.u.*) marked in these molecular graphs represent the charge density (*ρ*_b_) and the Laplacian of the charge density (∇^2^*ρ*_b_) at N···Cl bond critical point. Bond paths are in atom color and bond critical points between atomic basins are shown as tiny spheres in green. Bond distances and bond angles are in Å and degrees, respectively. Intermolecular contacts in (**a**,**b**,**e**) are colored in cyan and hanging contacts colored in red. N–H···Cl hydrogen bonds are not shown between the interacting ions in the crystal in (**a**).

Diazanediium dichloride, [NH_3_NH_3_][Cl]_2_, Figure 1a, is probably one of the simplest chemical adducts known in the crystalline phase. It was deposited in the CSD by Bolte in 2021 [81]; it is a cubic crystal (space group *Pa*$\bar{3}$), with lattice constant *a* = 7.8436 Å and ***Z*** = 4.

There are two types of intermolecular interactions that occur in the crystal lattice of [NH_3_NH_3_][Cl]_2_: H···Cl quasi-linear hydrogen bonds (∠N–H···Cl = 170.5°) and linear N···Cl (∠N–N···Cl = 180°), Figure 1b. The H···Cl hydrogen bonds are likely to be stronger than the N···Cl pnictogen bonds based on their respective bond distances (*r*(H···Cl) = 2.220 Å; *r*(N···Cl) = 3.061 Å). In both cases the intermolecular distances are directional and less than the sum of the vdW radii of the respective atomic basins (*r_vdW_*(H) = 1.20 Å; *r_vdW_*(N) = 1.66 Å; *r_vdW_*(Cl) = 1.82 Å). All van der Waals (vdW) radii quoted in this work are from [41]). 

Shown in Figure 1c is the geometry of the pnictogen bonds between the ion pairs [NH_3_NH_3_]^2+^ and 2Cl^−^ in the crystal, and the space-filling model showing the overlap between the N and Cl atomic basins is depicted in Figure 1d. For clarity, the H···Cl hydrogen bonds between the triangular face of the NH_3_ fragments in [NH_3_NH_3_] and the two chloride ions are not shown. They are abundant, and the H···Cl hydrogen bonds are clearly the secondary interactions while N···Cl pnictogen bonds are primary interactions, especially when the ion pairs [NH_3_NH_3_]^2+^ and 2Cl^−^ are in a geometrical arrangement shown in Figure 1c. 

The nature of the pnictogen bonding in crystalline [NH_3_NH_3_][Cl]_2_ is similar to that in the metal-free monofluorophosphate [N_2_H_6_][HPO_3_F]_2_ (CSD ref: GECXUL [82]), reported recently. The system exhibits a large birefringence (cal. 0.077) and wide band gap (∼6.51 eV), and the N···F pnictogen bond (and ∠N–N···F) in the structure are 2.803 Å (and 176.8°), among other O–H···O and N–H···O hydrogen bonded interactions. 

**Figure 2:**
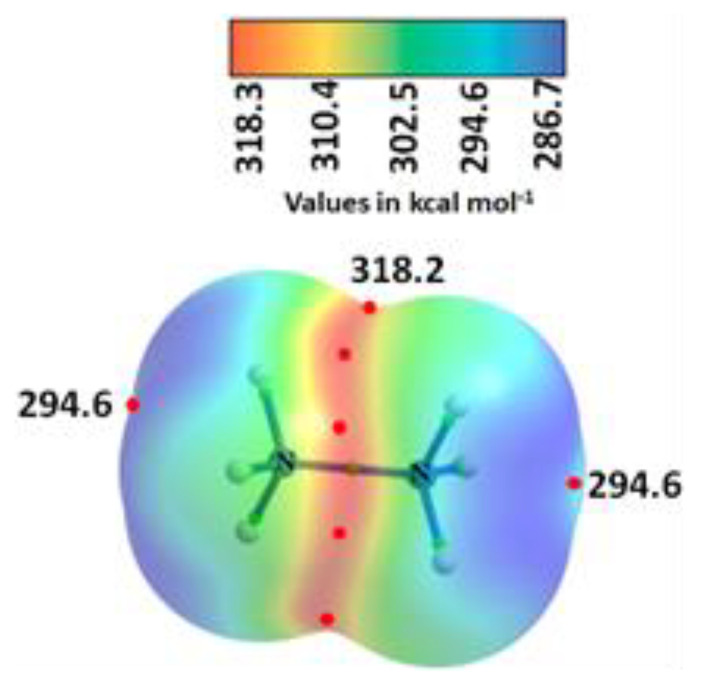
MP2/Aug-cc-pVTZ level 0.001 *a.u.* (electrons Bohr^−3^) isoelectron density envelope mapped potential on the electrostatic surface of [NH_3_NH_3_]^2+^. There is no local most minima (*V_S_*_,*min*_), but only local most maxima *V_S_*_,*max*_ (tiny red circles) identified on the surface of the two N atoms along N–N covalent bond extensions and around the central bonding region of the same bond.

The emergence of H···Cl hydrogen bonds and N···Cl pnictogen bonds in the [NH_3_NH_3_][Cl]_2_ crystal is not surprising since the electrostatic surfaces of both H and N atoms in [NH_3_NH_3_]^2+^ are strongly positive, where the former are more positive than the latter. This is clearly evident in the MESP model (Figure 2); the *V_S_*_,*max*_ associated with the two N atoms are equivalent due to symmetry (*V_S_*_,*max*_ = 294.6 kcal mol^−1^ each). 

Although the MESP model provides a useful guide to identify the two types of intermolecular bonding interactions between the interacting molecular entities of the ion-pair [NH_3_NH_3_][Cl]_2_, the results of the IGM-δ*g* analysis, presented in Figure 1e, strengthens the conclusion. As expected, there are two greenish isosurface volumes appearing between interacting units [NH_3_NH_3_]^2+^ and [Cl]^−^ that signify the possibility of pnictogen bonding; the flat nature of the isosurface volume between N and Cl indicates the delocalized nature of this non-covalent interaction and possible formation of weak H···Cl hydrogen bonds. However, the QTAIM-based molecular graphs presented in Figure 1f,g, which were obtained using the geometry of the ion-pair as in the crystal and that optimized at the MP2(FC)/def2-TZVPPD level, respectively, provide evidence of the formation of the charge assisted N···Cl pnictogen bonds in [NH_3_NH_3_][Cl]_2_. This means that the bond path and bcp topologies do not reveal the supposedly weak hydrogen-bonded interactions between the ion-pair. This scenario is very similar to that found for the [CD_3_ND_3_][I] and [CH_3_NH_3_][I] ion-pairs discussed below. 

The pnictogen bond present between [NH_3_NH_3_]^2+^ and [Cl]^−^ in the ion-pair in the crystal geometry is weaker than in the gas phase geometry where the intermolecular distance is markedly shorter. This explains why the bond path between N and C in Figure 1f is presented as a dotted line, and that in Figure 1g as a solid line, and the charge densities at the bcps of the corresponding bonds differ markedly from each other (*ρ*_b_ 0.0144, 0.0347 and 0.0327 *a.u.* on the crystal, MP2(FC)/def2-TZVPPD, and ωB97XD/def2-TZVPPD geometries of the ion-pair, respectively). Similarly, ∇^2^*ρ*_b_ at the N···Cl bcps of the corresponding geometries were 0.0388, 0.1422 and 0.1359 *a.u.*, respectively. Both properties indicate the closed-shell nature of the interaction, evidenced by ∇^2^*ρ*_b_ > 0 at the N···Cl bcp, and small value of *ρ*_b_ at the same bcp [65,67].

We have calculated the interaction energy between the ion pairs [NH_3_NH_3_]^2+^ and 2[Cl]^−^ using the MP2(FC) optimized geometry of [NH_3_NH_3_][Cl]_2_. We considered ([NH_3_NH_3_][Cl])^+^ as one fragment and [Cl]^−^ as another fragment to assess the interaction of a single charge assisted N···Cl pnictogen bond. The uncorrected and basis-set-corrected interaction energies, *E*_int_ and *E*_int_(BSSE), respectively, were calculated to be –145.64 and –144.10 kcal mol^−1^, respectively. The effect of BSSE on energy is marginal; the large interaction energy is largely due to cation–anion electrostatic interaction. This can be effectively rationalized based on the interaction energy of each N···Cl pnictogen bond in the ion-pair evaluated using the empirical relationship, *E*_b_ = ½*V*_b_, which was –11.45, –10.10 and –2.60 kcal mol^−1^ when the geometry of the ion-pair optimized with MP2(FC)/def2-TZVPPD, ωB97XD/def2-TZVPPD and that in the crystal was used, respectively. These energies correlate well with the N···Cl intermolecular bond distance of 2.572, 2.595 and 3.065 Å in the corresponding ion-pair geometry, respectively. 

Second order NBO analysis at RHF/6-311G* enabled us to demonstrate that the N···Cl pnictogen bond in NH_3_NH_3_Cl_2_ is indeed stronger than the N–H···Cl hydrogen bonds. Because the latter ones were very weak, they were unidentifiable via the bond path and bcp topologies of QTAIM (Figure 1f,g). There are two equivalent N···Cl pnictogen bonds formed by each end N site in NH_3_NH_3_^2+^ and the two interacting Cl ions situated along the extension of the N–N bond axis. The *ω*B97XD/def2-TZVPPD optimized gas phase geometry has resulted in hyperconjugations of two types, [LP(4)Cl → BD*(1) N–N] and [LP(4)Cl → RY*(1)N], that describe each of the two N···Cl pnictogen bonds in [NH_3_NH_3_][Cl]_2_, where LP and BD* refer to the lone-pair and anti-bonding orbitals, respectively. These two hyperconjugative interactions are accompanied with *E*^(2)^ of 10.95 and 6.16 kcal mol^−1^, respectively. Similarly, the hyperconjugations associated with two pairs of six N–H···Cl hydrogen bonds are described by [LP(4)Cl → BD*(1) N–H] and [LP(2)Cl → BD*(1) N–H] charge transfer delocalizations, with *E*^(2)^ of 1.24 and 1.28, respectively. The [LP(3)Cl → BD*(1) N–H] delocalization is also feasible in the system between the interacting units, with *E*^(2)^ of 0.43 kcal mol^−1^ for two and 1.71 kcal mol^−1^ for the remaining hydrogen bonds. When the crystal geometry of the ion-pair was used, the two types of non-covalent interactions remain identifiable. The only difference is that the magnitude of *E*^(2)^ is greatly reduced. This is understandable since the N···Cl intermolecular distance in the gas phase geometry is shorter than that in the crystal geometry. The *E*^(2)^ associated with [LP(4)Cl → BD*(1) N–N] and [LP(4)Cl → RY*(1)N], where RY refers to a Rydberg type orbital, which accompany each of the two pnictogen bonds, were 4.33 and 3.03 kcal mol^−1^, respectively. The charge transfer interactions associated with the N–H···Cl hydrogen bonds were about 0.1 kcal mol^−1^. These results show that the pnictogen bonds in the [NH_3_NH_3_][Cl]_2_ ion-pair are stronger than the hydrogen bonds. Since this study was primarily centered on pnictogen bonds, we did not explore the conformational details of hydrogen bonds, such as those shown in Figure 1b, that also play an important role in the effective stabilization of the crystal lattice. 

### 3.2. Methylammonium Lead Halide Perovskites 

Crystals containing the methylammonium action (MA, CH_3_–NH_3_^+^) have been extensively studied, especially in the field of metal halide perovskites for applications in optoelectronics [26,83,84]. Monovalent MA is entirely electrophilic. A simple CSD search for this organic cation yielded 931 hits, showing its prominent role in the design of materials. Along with its derivatives, it appears prominently in the lead trihalide perovskites, viz. CH_3_NH_3_MX_3_ (M = Pb, Sn, Ge; X = Cl, Br, I). These materials have a band gap in the range 1.2–3.5 eV [26,84] in the far infrared to visible region, making some of them suitable for application in solar cells. However, the actual role of the organic cation in CH_3_NH_3_MX_3_ perovskites is not yet fully understood [85], despite numerous experimental and computational studies on this topic that have been reported. 

It has been suggested that the stability of the crystal structure of CH_3_NH_3_MX_3_ single perovskites is driven by H···X hydrogen bonds formed between the ammonium guest and the halide of the MX_6_ octahedra [25,26]. However, we have recently argued [25,26] that this is incorrect for orthorhombic CH_3_NH_3_MX_3_ (X = Br, I), since the overall geometric stability of these crystals arises from an environment consisting of an interplay between hydrogen, carbon and pnictogen bonded interactions. These are collectively responsible for the octahedral tilt in the low-temperature *Pnma* orthorhombic (o) phase of the CH_3_NH_3_PbI_3_ system. We show below that this feature also holds for other members of the same family. 

**Figure 3:**
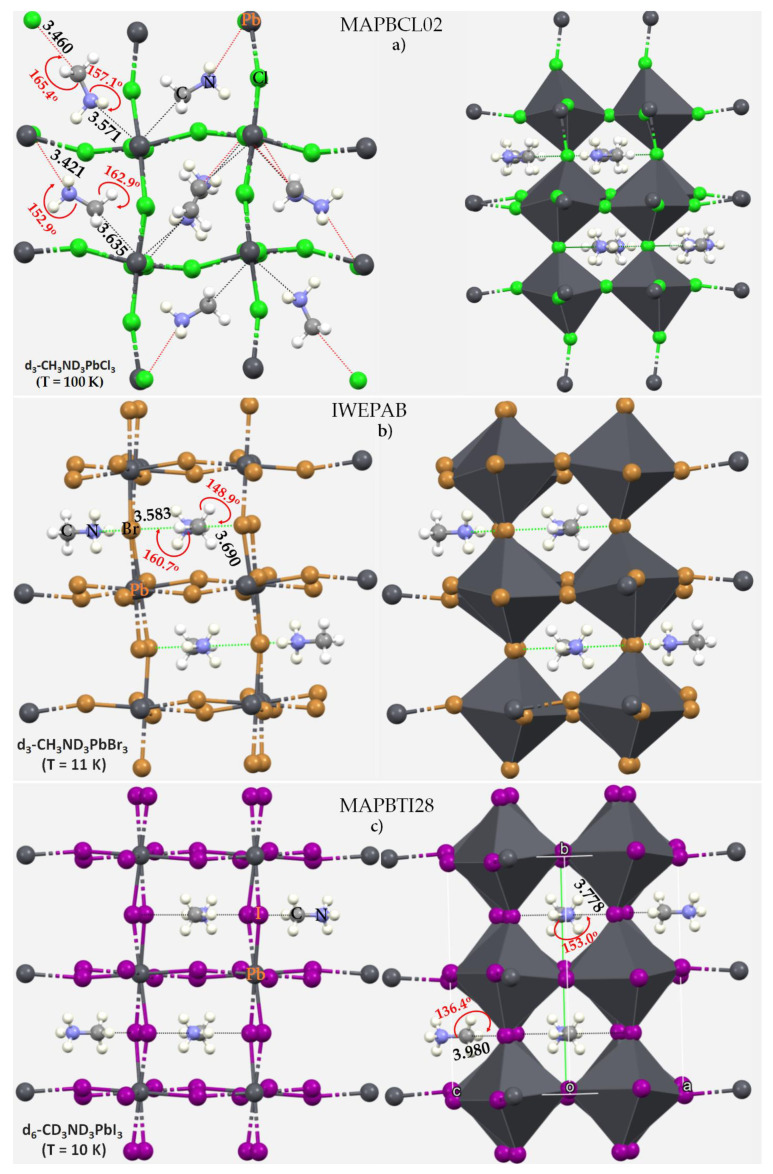
Ball-and-stick (**left**) and polyhedral (**right**) models of the low-temperature orthorhombic phase of organic-inorganic hybrid lead halide perovskites: (**a**) o-CH_3_ND_3_PbCl_3_ [86], (**b**) o-CH_3_ND_3_PbBr_3_ [87] and (**c**) o-CD_3_ND_3_PbI_3_ [88]. The bond distances, *r*(N···X) in Å, and bond angles, ∠R–N/C···X (R = C, N) in degrees, associated with the N···X and C···X (X = Cl, Br, I) pnictogen bonding and tetrel bonding interactions between the organic and inorganic frameworks are shown in each case. Dotted lines between atoms represent non-covalent interactions. Atom labeling is shown in each case, and H/D atoms are tiny spheres in white-gray. The N-H/N-D···X and C-H/N-D···X hydrogen bonds/deuterium bonds are not shown, and the CSD references are shown in capital letters. The orientation of the crystallographic axes in (**a**) is not the same as that in (**b**,**c**).

The structures of o-CH_3_ND_3_PbCl_3_, o-CH_3_ND_3_PbBr_3_, and o-CD_6_ND_6_PbCl_3_ are shown in Figure 3a–c. They are stable at low temperature and adopt an orthorhombic crystal symmetry with tilting of the PbX_6_ octahedra with the organic cation trapped inside the inorganic cage. There are a variety of N-H···X and C-H···X hydrogen bonds in each of these crystals; these are probably the primary interactions.

The primary intermolecular interactions in these crystals are charge-assisted, and strong. There are also secondary interactions that appear as pnictogen bonds and tetrel bonds (N···X and C···X (X = Cl, Br, I), respectively). These are somewhat longer, and therefore weaker, than the primary interactions. Based upon the bond distances, the stability of these interactions is in the order (N)D···X > (C)H/D···X > N···X > C···X (X = Br, I). For instance, in o-CD_3_ND_3_PbI_3_ [88] the shortest (N)D···I bonds (and (∠N–D···I angles) formed by each ammonium head are 2.672 (174.6°), 2.784 (147.3°) and 2.784 Å (147.3°); the three (C)D···I bonds (and (∠C–D···I angles) formed by the methyl head are 3.260 (177.1°), 3.175 (148.6°) and 3.175 Å (148.6°); and the N···I and C···I close contacts (∠C–N···I and ∠N–C···I angles) have distances (bond angles) of 3.778 (153.0°) and 3.980 Å (146.4°), respectively. Whereas the stability preference mentioned above is valid for o-CH_3_ND_3_PbBr_3_ [87] and o-CD_3_ND_3_PbI_3_ [88], this is not the case in o-CH_3_ND_3_PbCl_3_ [86] because the N···Cl and C···Cl pnictogen- and tetrel-bonds in the crystal appear alternately; hence the two orientations of the MA cation in the crystal have different types of N···Cl and C···Cl bond distances (cf. Figure 3a). Although the C···Cl tetrel bond in both the orientations are somewhat more directional (∠N–C···Cl = 165.4° or 162.9°) than the N···Cl pnictogen bonds (∠C–N···Cl = 157.1° or 152.9°), the N···Cl and C···Cl bond distances associated with one orientation are 3.460 and 3.571 Å, and those with the other orientation are 3.635 Å and 3.421 Å (Figure 3a). From this and, given that directional interactions are stronger than weakly directional interactions, it is difficult to determine whether the N···Cl pnictogen bonds are stronger than C···Cl tetrel bonds in o-CH_3_ND_3_PbCl_3_. 

Because the vdW radii of Cl (1.82 Å), Br (1.86 Å) and I (2.04 Å) are different, N···X and C···X bond distances follow the same order. They are shortest (and strongest) in the order: o-CH_3_ND_3_PbCl_3_ > o-CH_3_ND_3_PbBr_3_ > o-CD_3_ND_3_PbI_3_ (cf. Figure 3).

**Figure 4:**
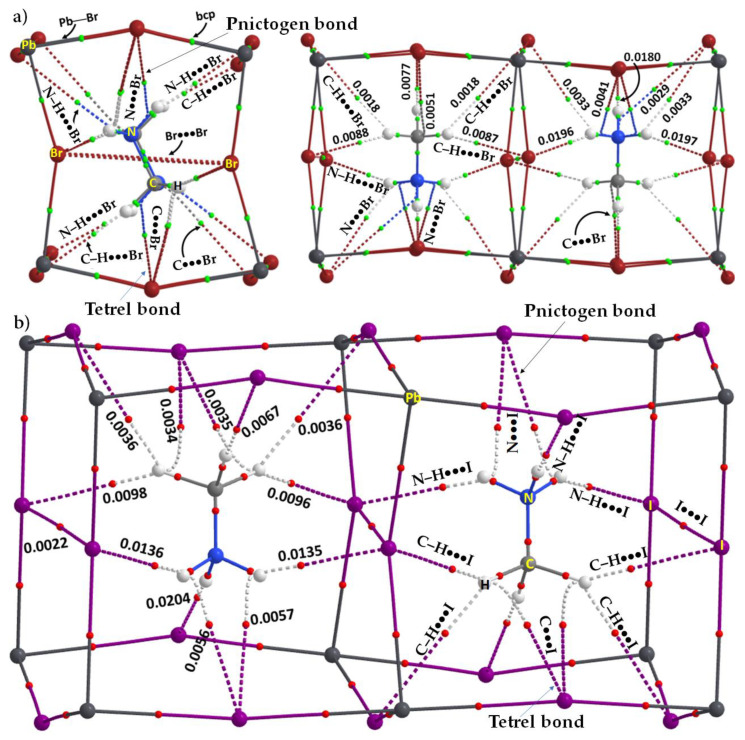
(**a**) QTAIM-based molecular graph and (3, –1) bond critical point charge density topologies (*ρ*_b_) for (**a**) o-CH_3_NH_3_PbBr_3_ and (**b**) o-CH_3_NH_3_PbI_3_, obtained with PBE/DZP, very similar to what reported by us elsewhere [25,26]. The PBE relaxed geometry for o-CH_3_NH_3_PbBr_3_ and the neutron diffraction geometry (T ≈ 100 K [89]) for o-CH_3_NH_3_PbI_3_ were used. Atoms: iodine—purple, carbon—dark-grey, nitrogen—deep-blue, hydrogen—white-grey; bond paths are shown as solid and dotted lines in atom color; (3, –1) bond critical points are shown as tiny blue and red spheres between bonded atomic basins. Dotted lines (bond paths) indicate the presence of non-covalent interactions between atoms that are liked with each other. Values of *ρ*_b_ are in *a.u.*

The presence of hydrogen, carbon and pnictogen bonding interactions in crystalline o-CH_3_NH_3_PbX_3_ has been confirmed by QTAIM and RDG analyses reported elsewhere [25,26]. The results are shown in Figure 4 for o-CH_3_NH_3_PbBr_3_ and o-CH_3_NH_3_PbI_3_. Although the principal bonding features are evident from an analysis of the intermolecular geometry alone, the bond path and bcp, as well as the isosurface topologies of QTAIM and RDG suggested that each H atom in the ammonium/methyl head in MA could be involved with one/two Br atoms to form one/two (N)H···Br/(C)H···Br hydrogen bonds in o-CH_3_NH_3_PbBr_3_ described by the bcps (tiny spheres in green) between the bonded atomic basins. Bond paths between N and C in MA and the Br sites are also apparent, indicating the presence of N···Br and C···Br pnictogen- and tetrel-bonds in the crystal. 

Because the (N)H···I and (C)H···I hydrogen bonds in o-CH_3_NH_3_PbI_3_ are longer than those in o-CH_3_NH_3_PbBr_3_, the QTAIM results suggest the formation of a single (N)H···I hydrogen bond by each H atom of the –NH_3_ group and at least two (C)H···I bonds formed by each of the H atoms of –CH_3_ group of MA (Figure 4b). The inward curvature of the bond paths between N or C in MA and the nearest I sites indicates that the N···I and C···I pnictogen- and tetrel-bonds in o-CH_3_NH_3_PbI_3_ are dispersive and weak. The nature of the bond path topologies associated with these interactions is very similar to that found for o-CH_3_NH_3_PbBr_3_ (see Figure 4a,b). The charge density values depicted in Figure 4 suggest that they are very small at the bcps and are typical of non-covalent interactions (*ρ*_b_ < 0.05 au). It is also apparent that *ρ*_b_ at the N···I and C···I bcps are smaller than those at the (N)H···I and (C)H···I bcps, confirming that first two are weaker than the last two. These analyses highlight the co-existence of several types of non-covalent interactions in these halide perovskite structures, including I···I (Figure 4a) and Br···Br (Figure 4a) halogen-halogen interactions. 

### 3.3. Methylammonium and Deutero-Methylammonium Halides

**Figure 5:**
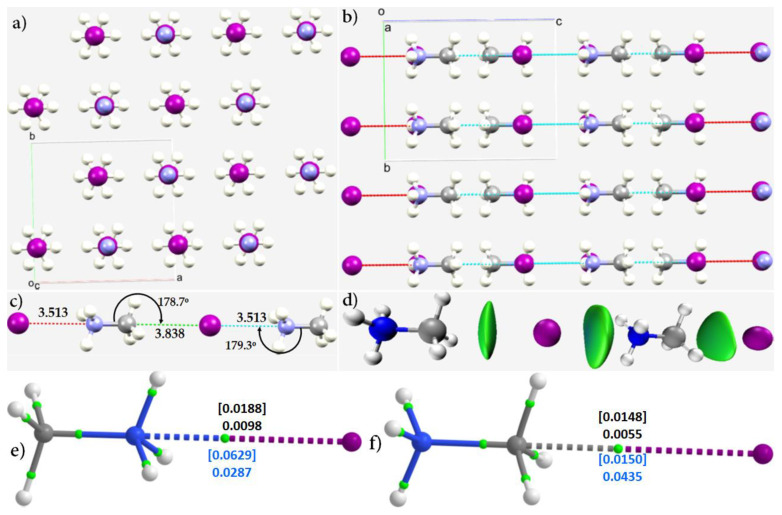
(**a**,**b**) Two different views of the ball-and-stick model of the 2 × 2 × 2 supercell structure of the crystal of perdeuteromethylammonium iodide, [CD_3_ND_3_][I] (CSD ref: KUBNOK), with the unit-cell, together with the three crystallographic axes, shown for each case; (**c**) the nature of intermolecular bond distances and intermolecular bond angles between CD_3_ND_3_^+^ and I^−^; and (**d**) IGM-δ*g* based 0.004 *a.u.* isosurface volume (colored bluish-green) between N/C and I atomic basins of interacting units CD_3_ND_3_^+^ and I^−^ responsible for the structure of the crystal. (**e**,**f**) QTAIM based molecular graphs for H_3_C-H_3_N···I and H_3_N-H_3_C···I, respectively, obtained using *ω*B97X-D/def2-TZVPPD with the geometries of the same ion-pairs as in the crystal (CSD ref. KUBNOK02). Values (in *a.u.*) in black and blue in (**c**,**d**) represent the charge density (*ρ*_b_) and the Laplacian of the charge density (∇^2^*ρ*_b_) at N···I (and C···I) bond critical point(s), respectively, obtained using the geometry of the adduct as in the crystal. The corresponding values calculated on the *ω*B97X-D/def2-TZVPPD fully relaxed geometries of H_3_C-H_3_N···I and H_3_N-H_3_C···I are shown in square brackets, respectively. Bond paths are in atom color and bond critical points between atomic basins are shown as tiny spheres in green. Bond distances and bond angles are in Å and degrees, respectively. Intermolecular contacts are colored in cyan/green and hanging contacts are colored in red. Atoms shown as balls are colored iodine—purple; nitrogen—blue; carbon—gray; hydrogen—white-gray.

Two different views of the crystal structure of perdeuteromethylammonium iodide, [CD_3_ND_3_][I] (CSD ref. KUBNOK [90]), reported using neutron diffraction measurements on a powder sample, are shown in Figure 5a,b. It crystallizes in the *Pbma* space group with *Z* = 4. The *P_4_/nmm* tetragonal crystal of the same compound is known, with Z = 2, but the D atom positions were not accurately determined (CSD ref. KUBNOK01 [90]). The hydrogen analogue, [CH_3_NH_3_][I], has been reported (CSD ref. KUBNOK02 [91], Z = 2), but the H atom positions were not accurately determined. As discussed above for the [NH_3_NH_3_][Cl]_2_ crystal (Figure 1), it appears that the D···I and H···I deuterium and hydrogen bonds, N···I pnictogen bonds and C···I tetrel bonds play a structure-determining role in [CD_3_ND_3_][I] and [CH_3_NH_3_][I]. The dotted lines in cyan between atomic basins in Figure 5b,c indicate the presence of the latter interactions in [CD_3_ND_3_][I], and are substantially longer than those found in [NH_3_NH_3_][Cl]_2_; this is expected since the sum of the vdW radii of C and I, as well as that of N and I, is greater than that of N and Cl (*r_vdW_*(N) = 1.66 Å; *r_vdW_*(C) = 1.77 Å; *r_vdW_*(Cl) = 1.82 Å; *r_vdW_*(I) = 2.04 Å).

From Figure 5c, it is apparent that the N···I pnictogen bond and the C···I tetrel bond in the perdeuteromethylammonium iodide crystal (CSD ref: KUBNOK, space group *Pbma*) are near-linear interactions, with ∠C–N···I = 179.3° and ∠N–C···I = 178.7°. The former are stronger than the latter (*r*(N···I) = 3.513 Å vs. *r*(C···I) = 3.838 Å), a bond distance which is smaller than the sum of the vdW radii N and I, 3.70 Å; the value of *r*(C···I) is marginally longer than the sum of the vdW radii of C and I, 3.81 Å, which suggests they are dispersive in nature. By contrast, the (N)D···I and (C)D···I deuterium bonds are inequivalent because the –ND_3_ group is more acidic than the –CD_3_ group in d_6_-MA. For instance, the bond distance (N)D···I in the crystal appears in three different flavors, with (*r*(N)D···I)) (∠N–D···I) 2.640 Å (154.5°), 2.715 Å (141.0°) and 3.257 Å (100.9°). Similarly, the bond distance of (C)D···I close contacts appears in two different flavors, with (*r*((C)D···I)) (∠C–D···I) 3.210 Å (125.4°) and 3.472 Å (107.9°). 

In the case of the tetragonal [CD_3_ND_3_][I] crystal (CSD ref: KUBNOK01, space group *P4/nmm*), ∠C–N···I = 180.0° and ∠N–C···I = 180.0°. These correspond to *r*(N···I) of 3.543 Å and *r*(C···I) of 4.046 Å, respectively. For tetragonal [CH_3_NH_3_][I] (CSD ref: KUBNOK02, space group *P4/nmm*), ∠C–N···I = 180.0° and ∠N–C···I = 180.0°; they correspond to *r*(N···I) of 3.588 Å and *r*(C···I) of 3.999 Å, respectively. 

**Figure 6:**
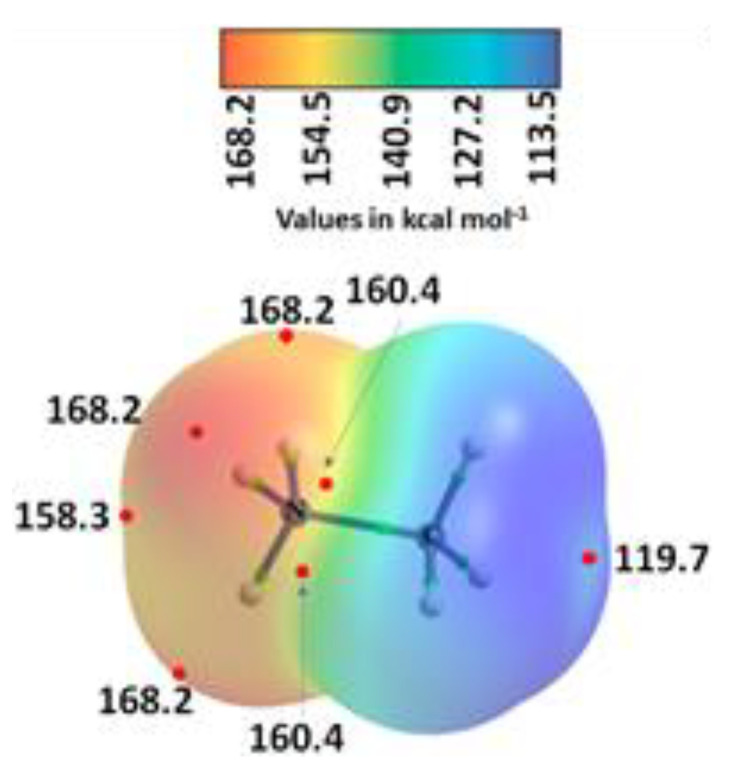
MP2/Aug-cc-pVTZ level 0.001 *a.u.* isoelectron density-envelope-mapped potential on the electrostatic surface of methylammonium, CH_3_NH_3_^+^). There are no local most potential minima (*V_S_*_,*min*_), but only local most maxima *V_S_*_,*max*_ (tiny red circles) identified on the surfaces of the two N atoms along the N–N bond extensions, and around the central region of the N–N covalent bond.

The attractive interactions between CH_3_NH_3_^+^ and I^−^ accord with the results of the MESP model, Figure 6. N is more electropositive than C in CH_3_NH_3_^+^ (*V_S_*_,*max*_ = 158.3 kcal mol^−1^ for N and 119.7 kcal mol^−1^ for C), and N interacts more strongly than C with I^−^. Both N···I and C···I are directional interactions. Their presence is evident in the IGM-δ*g* plot (Figure 5d), in which the isosurfaces are thicker and wider between N and I than that between C and I. 

Our QTAIM calculations provide further evidence of the presence of attractive interactions between the bonded atomic basins in [CH_3_NH_3_][I], Figure 5e,f. There is a bcp between N and I (Figure 5e) and between C and I (Figure 5f). They are present regardless of whether the geometry of the ion-pair was extracted from the crystal, or from the fully relaxed structure using MP2(FC) and *ω*B97X-D geometries evaluated in conjunction with def2-TZVPPD. The charge density (*ρ*_b_) values at the N···I (or C···I) bcps shown in Figure 5e (or Figure 5f) for [CH_3_NH_3_][I] are different since the intermolecular N···I (or C···I) distance in the relaxed gas phase geometry of [CH_3_NH_3_][I] was appreciably shorter than that in the crystal. However, they are indeed less than the typical value of *ρ*_b_ (*ρ*_b_ < 0.05 au) often reported for non-covalent interactions [92,93]. The Laplacian of the charge density (∇^2^*ρ*_b_) values at the N···I (or C···I) bcp are also unequal and are positive (∇^2^*ρ*_b_ > 0), and hence attest to the closed-shell nature of these interactions [65,66,67]. Although the IGM-δ*g* plot indicated N···I and C···I pnictogen and tetrel bonding interactions (as well as (N)D···I and (C)D···I deuterium bonds) between the interacting monomers in the ion-pair [CD_3_ND_3_][I], the (N)D···I and (C)D···I deuterium bonds (or (N)H···I and (C)H···I hydrogen bonds) were not identified in [CD_3_ND_3_][I] (or [CH_3_NH_3_][I]) using the bond path and bcp topologies of QTAIM when the interacting ions are in a geometric arrangement shown in Figure 5e,f.

We have calculated the interaction energy of the two aforementioned ion-pairs of [CH_3_NH_3_][I], Figure 5e,f using the crystal, as well as using their MP2(FC) and *ω*B97X-D/def2-TZVPPD, geometries. The results are summarized in Table 1. Clearly, the interaction energy is very large regardless of the geometry of the ion-pair used, and that the ion-pair with the longer intermolecular distances is predicted to have the smaller interaction energy, as expected. Specifically, the interaction energy of the pnictogen bonded ion-pair H_3_C-H_3_N···I was found be larger than that of the carbon bonded ion-pair H_3_N-H_3_C···I present in [CH_3_NH_3_][I], and is typical of a Mulliken inner complex [94]. The strength of the interaction energy found for these systems is comparable to that in analogous systems reported in previous studies [85]. 

The strength of the carbon bond in the conformer shown in Figure 5f based on *E*_b_ = ½*V*_b_ gave values of −2.79 and −0.75 kcal mol^−1^ when evaluated on the *ω*B97X-D and crystal geometries of the ion-pair, respectively. Similarly, the strength of the pnictogen bond in the conformer shown in Figure 5e was −4.64 and −1.79 kcal mol^−1^ when evaluated on the *ω*B97X-D and crystal geometries of the ion-pair, respectively. Since there was no bond path between the H atoms of –NH_3_ and –CH_3_ fragments of the CH_3_NH_3_ cation and the interacting iodide anion, the strength of hydrogen bonds between them could not be evaluated. These results suggest that pnictogen bond is the strongest of all interactions investigated.

Zeroth-order symmetry-adapted perturbation theory (SPAT0 [95]) calculations were performed for the [CH_3_NH_3_][I] adduct, on the structure extracted from the crystal and on the *ω*B97X-D and MP2(FC) optimized geometry, in conjunction with the def2-TZVPPD-RI basis set. This approach dissects the interaction energy into four major components arising from electrostatics (*E*_els_), exchange-repulsion (*E*_exch_), induction (*E*_ind_) and dispersion (*E*_disp_). The results are summarized in Table 2. As can be seen, the total interaction energies *E*_int_(SAPT0) obtained using SAPT0 are in reasonable agreement with those computed using the counterpoise method of the SCF (Self-Consistent Field) approaches (*ω*B97X-D and MP2(FC)). Inspection of the dissected components suggests that the energy due to electrostatics is significant as expected given that [CH_3_NH_3_][I] is an ion-pair and hence coulombic attraction plays a very crucial role in stabilizing the intermolecular interaction. Since the intermolecular distances in the adducts in the crystal geometry are longer than those in the *ω*B97X-D and MP2(FC) optimized geometries, the interaction between the monomers becomes stronger in the latter case than in the former. Concomitant with this is the increase in each of the dissected components (see Table 2).

The collective contribution due to induction and dispersion cannot be overlooked since they contribute appreciably to the net interaction energy *E*(SAPT0). Whereas *E*_exch_ is repulsive, and its magnitude is comparable with the induction energy (viz. 4.4 kcal mol^−1^ for H_3_N-H_3_C···I) when the crystal geometries of the H_3_C-H_3_N···I and H_3_N-H_3_C···I ion-pairs were used, the net interaction energy *E*(SAPT0) is then due to energies arising from electrostatics and dispersion. However, when the *ω*B97X-D and MP2(FC) geometries of the H-containing ion-pairs were used, each of the four components responsible for *E*_int_(SAPT0) are significantly affected. This is not very surprising since the intermolecular distances in H_3_C-H_3_N···I and H_3_N-H_3_C···I were significantly reduced in the gas phase structure. This has caused an increase in *E*_els_, *E*_ind_ and *E*_disp_, with concomitant increase in *E*_exch_. Even so, the *E*_int_(SAPT0) value calculated on the *ω*B97X-D and MP2(FC) geometries of the two ion-pairs are in reasonable agreement with the corresponding *E*_int_(*ω*B97X-D) and *E*_int_([MP2(FC)]), respectively (see Table 2). 

The strengths of the pnictogen bond in H_3_C-H_3_N···I and the tetrel bond in H_3_N-H_3_C···I are significant when compared to the N-H···I and C-H···I hydrogen bonds in the corresponding ion-pairs. This is also evidenced by our second order analysis of the charge transfer delocalization energy [72,73,74,75,96]. This was carried out at the RHF/6-311G* level using the MP2(FC) and *ω*B97X-D optimized geometries. Our results are summarized in Table 3. The data show that for the formation of the N···I pnictogen bond and N-H···I hydrogen bonds, the lone-pair(4) on the iodide anion, LP(4)I, interacts attractively via a charge transfer process with the σ* type C–N and N–H anti-bonding orbitals BD* of the organic cation NH_3_CH_3_^+^, respectively. The magnitude of second order energy *E*^(2)^ associated with the two types of charge transfer delocalizations is 4.51 and 1.14 kcal mol^−1^, respectively. In addition, we have also identified that the same lone-pair on I is involved in a charge transfer delocalization with a Rydberg type orbital RY*(1) on N with an *E*^(2)^ of 2.86 kcal mol^−1^. There are, in fact, three weak N-H···I hydrogen bonds, in which LP(2), LP(3) and LP(4) are simultaneously involved in hyperconjugative charge transfer interactions with the three BD*(N–H) orbitals. 

The nature of the hyperconjugative charge transfer delocalization is very similar between the interacting units CH_3_NH_3_^+^ and I^−^, leading to the formation of the ion-pair H_3_N-H_3_C···I. However, in this case, the three C-H···I hydrogen bonds are markedly weaker (compared to the N-H···I hydrogen bonds), with *E*^(2)^ energies close to 0.12 kcal mol^−1^. By contrast, the charge transfer delocalization is very pronounced between LP(4)I and BD*(1)C–N anti-bonding orbital of CH_3_NH_3_^+^, with *E*^(2)^ [LP(4)I → BD*(C–N)] = 10.22 kcal mol^−1^. Other than this, there are LP(4)I → RY*(C) and LP(1)I → BD*(C–N) delocalizations with *E*^(2)^ of 2.58 and 0.56 kcal mol^−1^, respectively. 

The results given above were obtained on the MP2(FC) geometries; they are also evident when the *ω*B97X-D geometries of the ion-pair was used, and *E*^(2)^ values are slightly smaller, in accordance with the longer intermolecular distance between CH_3_NH_3_^+^ and I^−^. These results suggest that the triangular face in –NH_3_ or –CH_3_ is simultaneously engaged with the iodide anion, leading to the stability of the H_3_C-H_3_N···I and H_3_N-H_3_C···I ion-pairs, respectively. While we did not explore the conformational space, it is no doubt that the crystal also contains a significant number of C-H···I and N-H···I hydrogen bonds of another variety. 

### 3.4. Ammonium Cyanate

The transformation of ammonium cyanate into urea was first studied over 170 years ago by Wöhler and Liebig [97]. Knowledge of the crystal structure of ammonium cyanate is a prerequisite for a basic understanding of the nature of its solid-state reaction. The neutron powder diffraction data have enabled MacLean et al. [97] to demonstrate the formation of N−H···N hydrogen bonds between an NH_4_^+^ ion to four cyanate N atoms at alternate corners of a distorted cube, overturning their initial own view of the presence of N−H···O hydrogen bonds to cyanate O atoms at the other four corners.

**Figure 7:**
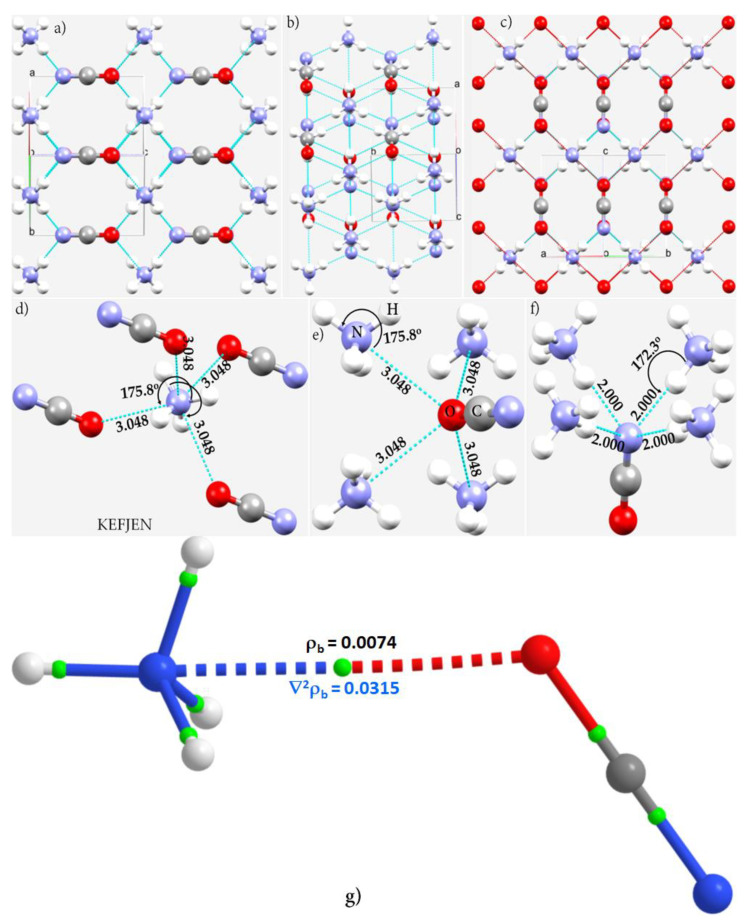
(**a**–**c**) Three different views of the ball-and-stick model of the 2 × 2 × 2 supercell structure of the crystal of ammonium cyanate, NH_4_CNO (CSD ref. KEFJEN); (**b**–**f**) The local nature of intermolecular bond distances and intermolecular bond angles between NH_4_^+^ and CNO^−^, showing the coordination environment around (**d**) N in NH_4_^+^, (**e**) O in CNO^−^, and (**f**) N in CNO^−^. Bond distances and bond angles are in Å and degrees, respectively. Intermolecular contacts are colored in cyan and hanging contacts in **c** are colored in red. (**g**) MP2(FC)/def2-TZVPPD level QTAIM-based molecular graph for the ion-pair NH_4_CNO, obtained on its geometry as in the crystal; it shows the bond paths as sticks in atom color (N—blue; C—grey; O—red; H—white grey), bond critical points as tiny spheres between atomic basins in green, and values (in *a.u.*) of charge density ρ_b_ and the Laplacian of the charge density ∇^2^ρ_b_ at the N···O bond critical point.

**Figure 8:**
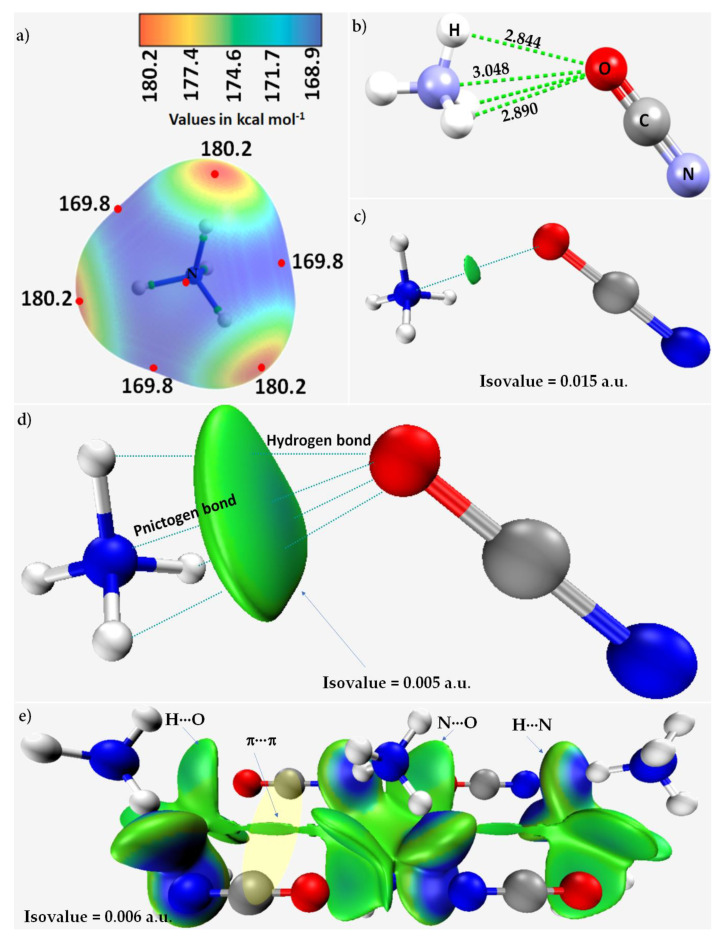
(**a**) The 0.001 *a.u.* isoelectron density mapped potential on the electrostatic surface of the NH_4_^+^ cation, obtained using MP2/Aug-cc-pVTZ; the tiny circles in red represent the local most maxima of electrostatic potential, *V_S_*_,*max*_. (**b**) The local nature of N–H···O hydrogen bonding and H–N···O pnictogen bonding interactions between the NH_4_^+^ and CNO^−^ ions in NH_4_CNO. (**c**,**d**) IGM-δ*g* based isosurface volumes (colored bluish-green) between NH_4_^+^ and CNO^−^ ions, with two different isovalues of IGM. (**e**) IGM-δ*g* based isosurface volume (colored bluish-green) between NH_4_^+^ and CNO^−^ ions as in a 2 × 2 × 2 supercell structure of the system, showing different interaction types between the interacting moieties. Bond distances and bond angles are in Å and degrees, respectively. Intermolecular contacts in (**b**–**d**) are colored in cyan/green.

We investigated the intermolecular geometry of the NH_4_CNO crystal with MESP and IGM-δ*g* calculations. The results we obtained support both proposals of the authors [97]. Three different views of the 2 × 2 × 2 supercell crystal structure of tetragonal ammonium cyanate (space group *P_4_/nmm*) are shown in Figure 7a–c. The local non-covalent interaction topologies between interacting atoms of the participating molecular entities are shown in Figure 7d–f. The MESP model description is given in Figure 8a; it is clear that N in NH_4_^+^ features four σ-holes (*V_S_*_,*max*_ 158.3 kcal mol^−1^ each), each along the outer extension of an H–N bond. There are also four σ-holes on four H atoms along the four N–H bond extensions that are somewhat stronger (*V_S_*_,*max*_ 180.2 kcal mol^−1^). N in NH_4_^+^ is engaged attractively with four nearest-neighbor O sites of four CNO^−^ anions, Figure 7d, thus forming four charge-assisted H−N···O pnictogen bonds that are equivalent (*r*(N···O) = 3.048 Å and ∠H–N···O = 175.8°). From Figure 7e, it is clear that each O site in a CNO^−^ anion acts as a Lewis base and is in attractive engagement with the N atoms of four nearest-neighbor NH_4_^+^ cations, forming four equivalent H−N···O bonds. These pnictogen bonds are all quasi-linear because of the competition with N−H···O hydrogen bonds that are simultaneously formed, since each NH_4_^+^ cation donates H atoms to cyanate O. Two of these non-linear hydrogen bonds are equivalent (*r*(H···O) = 2.890 Å and ∠N–H···O = 88.9°) and the other is slightly shorter (*r*(H···O) = 2.844 Å and ∠N–H···O = 91.5°); see Figure 8b). On the other hand, and from Figure 7f, one can conclude that the N of the CNO^−^ anion acts as a site to accepts four strong quasi-linear hydrogen bonds, each with *r*(H···N) = 2.000 Å and ∠N–H···O = 172.3°. From these results it is clear that NH_4_^+^ is a versatile hydrogen- and pnictogen bond donor, that O in CNO^−^ is a versatile pnictogen bond acceptor and that N in CNO^−^ is a versatile hydrogen bond acceptor. In all these interactions the intermolecular distance associated with the close contacts H···O, N···O or H···N are less than the sum of the vdW radii of the relevant atomic basins.

Shown in Figure 8c,d are the IGM-δ*g* based promolecular charge density plots between NH_4_^+^ and CNO^−^ ions, obtained using a geometry extracted from the crystal. From the size of the IGM-δ*g* volumes obtained using two different isovalues (0.015 and 0.005 *a.u.*), it is clear that the charge-assisted N···O pnictogen bond is moderately strong given its presence even at the higher isovalue of 0.015 *a.u.* The isosurface corresponding to the H···O close contacts that appears at low isovalues indicates that the H···O hydrogen bonds are weaker than the N···O pnictogen bonds (see Figure 8c,d). On the other hand, the isosurfaces between N and H are bluish as a result of more electron density concentration in the bonding region (cf. Figure 8e). The presence of (C)π···π(C) interactions in the crystal is also confirmed by the IGM-δ*g* analysis, with a contact distance of 3.644 Å. However, these interactions are probably the weakest of all the interactions identified in the crystal, as evidenced by the flat greenish isosurface between a pair of CNO^−^ ions (see Figure 8e).

To further clarify the presence of pnictogen bonding in NH_4_CNO, we have used the geometry of the ion-pair found in the crystal and performed QTAIM calculations on it at the MP2(FC)/def2-TZVPPD level. Similar to other ion-pairs discussed above and below, QTAIM did not indicate the formation of hydrogen bonding in the conformation shown in Figure 7g. However, it indeed revealed the presence of an N···O pnictogen bonding between the N and O atomic basins of the two interacting ion pairs. There is a bond path, and bond critical point between the bonded atomic basins, shown as dotted line in atom color between N and O. The *ρ*_b_ and ∇^2^*ρ*_b_ at the N···O bcp is as small as 0.0074 and 0.0315 au. The empirical expression, *E*_b_ = ½*V*_b_, gave values of −1.51 kcal mol^−1^ in the corresponding geometries, respectively, suggesting the possibility of a weak interaction between N and O in NH_4_CNO. Of course, this interaction energy is substantially smaller than the complexation energy found at the MP2(fc)/def2-TZVPPD level: the uncorrected and BSSE corrected energies of −88.16 and −87.80 kcal mol^−1^, respectively, arising primarily from the electrostatic interaction between two ionic moieties that cause the two ionic domains to come into close proximity. NBO’s second order analysis with RHF/6-311G* gave an indication of the occurrence of a LP(3)O → BD(1)* N–H hyperconjugative interaction, with an *E*^(2)^ of 0.56 kcal mol^−1^; however, there was no such appreciable hyperconjugative interaction revealed that could represent hydrogen bonding between NH_4_^+^ and CNO^−^ for the conformer shown in Figure 7g.

### 3.5. The Ammonium Halides

Referring to their 2007 work [98], Nelyubina et al. have shown in a subsequent 2014 study [99] that N···Cl close contacts could be present in crystalline NH_4_Cl. Experimental electron density data derived from X-ray diffraction measurements enabled them to provide an accurate description of the interionic bonding in NH_4_Cl, and gave results that were consistent with quantum chemistry based-calculations [98].

**Figure 9:**
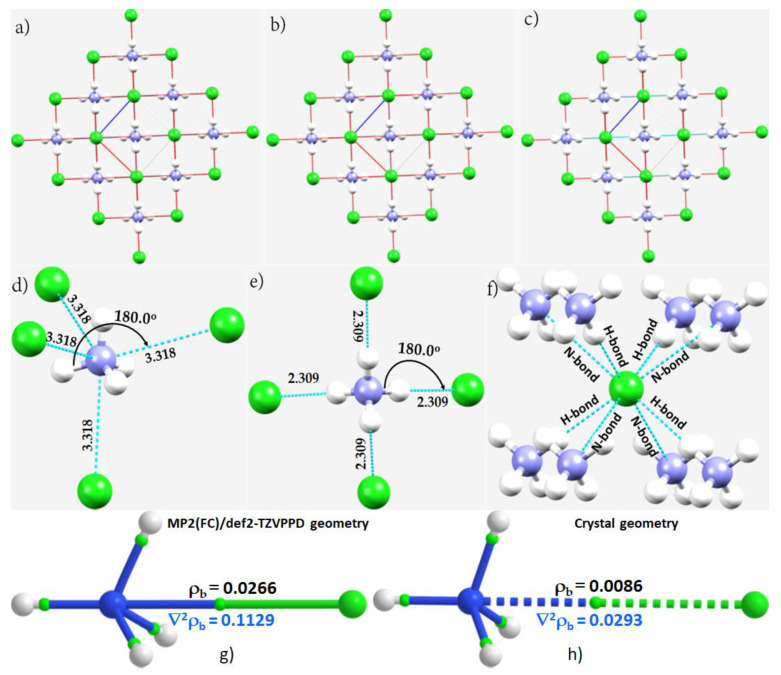
Three views of the crystal of NH_4_Cl (ICSD ref. 428519 [99]) showing (**a**) combined H···Cl hydrogen bonds and N···Cl pnictogen bonds, (**b**) N···Cl pnictogen bonds, and (**c**) H···Cl hydrogen bonds. Illustration of the local nature of multi-furcated charge-assisted intermolecular bonding topologies: (**d**) NH_4_^+^ as four pnictogen bond donors; (**e**) NH_4_^+^ as four H bond donors; and (**f**) Cl^−^ as four H-bond and four N-bond acceptors. Bond lengths and bond angles are in Å and degrees, respectively. The dotted lines in cyan/red represent inter-ionic interactions. (**g**,**h**) MP2(FC)/def2-TZVPPD level QTAIM-based molecular graph for the ion-pair NH_4_Cl, obtained on its MP2(FC)/def2-TZVPPD gas-phase geometry and as in the crystal, respectively; both these graphs show bond paths as sticks and dotted lines in atom color (N—blue; Cl—green; H—white grey), bond critical points as tiny spheres between atomic basins in green, and values (in *a.u.*) of charge density ρ_b_ and the Laplacian of the charge density ∇^2^ρ_b_ at the N···Cl bond critical point.

The crystal structure of NH_4_Cl is shown in Figure 9a–c, and features H···Cl hydrogen bonding and N···Cl pnictogen bonding. The first of these interactions is the stronger, as revealed from the bond distances (*r*(H···Cl) = 2.309 Å and *r*(N···Cl) = 3.318 Å), and both are linear (∠N–H···Cl = 180.0° and ∠H–N···Cl = 180.0°). From the local geometries shown in Figure 9d,e, it is clear that N is a donor of four σ-hole-centered pnictogen bonds, and simultaneously four hydrogen bond donors; this means that each NH_4_^+^ unit is surrounded by eight nearest-neighbor Cl^−^ anions in the crystal lattice. The former are engaged attractively with four nearest-neighbor chloride ions in forming four σ-hole centered charge-assisted H···Cl hydrogen bonds. Similarly, the four σ-holes on N in NH_4_^+^ are linked with four nearest-neighbor Cl ions, thereby forming four σ-hole-centered pnictogen bonds. This shows that the Cl^−^ ion is the versatile acceptor of eight non-covalent bonds (four H-bonds and four N-bonds), Figure 9f.

Nelyubina et al. [99] have estimated the energy of the pnictogen bonds to vary between 1.2 and 1.5 kcal mol^−1^, so each of them accounts for 20 to 25% of the interaction energy per NH_4_^+^ moiety in NH_4_Cl. As mentioned above, our view is consistent with that of these authors: the hydrogen bonds in the crystal are the primary interactions. They are the cause of the occurrence of the pnictogen bonds with the ammonium cation. Clearly, there is a straightforward interplay between these two types of bonding interactions that not only provides stability to the crystal structure, but also assists in the determination of phase transition behavior, ferroelastoelectric and other physical properties.

QTAIM calculations for the NH_4_Cl ion-pair were performed for both the crystal geometry and the MP2(FC)/def2-TZVPPD and *ω*B97X-D/def2-TZVPPD optimized geometries. As for the CH_3_NH_3_I case, we also found that the intermolecular distance between N and Cl in NH_4_Cl in the MP2(FC) gas phase, 2.701 Å, is significantly shorter than that of 3.318 Å in the same ion-pair in the crystal. Despite this difference, the topology of bond paths and bcp between the N and Cl atomic basins in the ion-pair is preserved: the *ρ*_b_ (and ∇^2^*ρ*_b_) values for the N···Cl bcp are 0.0266 (+0.1129), 0.0253 (+0.1083) and 0.0086 (+0.0293) *a.u.* with MP2(FC), ωB97X-D and crystal geometries, respectively, suggesting a closed-shell interaction. The empirical expression, *E*_b_ = ½*V*_b_, gave values of −8.35, −7.53 and −1.66 kcal mol^−1^ as the strength of the ion-pair in the MP2(FC)/def2-TZVPPD, *ω*B97X-D/def2-TZVPPD and crystal geometries of the ion pair, respectively, indicating that the bond between the N and Cl atomic basins of NH_4_Cl in the crystal geometry is quite weak; this result is in good agreement with that of Nelyubina et al. [99] (see above). However, the uncorrected (BSSE corrected) complex formation energies for the ion-pair calculated using MP2(FC) (*ω*B97XD/def2-TZVPPD) [crystal] geometries found to be −119.11 (−118.03) [−118.07] and −102.63 (−117.92) [−102.15] kcal mol^−1^, respectively, suggests BSSE has a negligible effect on the complexation energy, and the large complexation energy is of coulombic origin, as expected for ionic interactions.

Our RHF/6-311G* level second order NBO analysis suggested that delocalization of charge transfer promotes attraction between the interacting units. In particular, this analysis suggests two possible interactions, H···Cl and N···Cl, that stabilize the ion-pair configuration shown in Figure 9g. There are three equivalent close contacts of the former type, each with an *E^(^*^2)^ of [LP(4)Cl → BD*(1) H–N] = 0.73 kcal mol^−1^; similarly, the [LP(2)Cl → BD*(1) H–N] delocalization associated with the three H-bonded interactions were 0.74, 0.75 and 0.99 kcal mol^−1^. The nitrogen bonding in the ion-pair is described by hyperconjugative interactions [LP(4)Cl → BD*(1) N–H], [LP(1)Cl → BD*(1) N–H], and [LP(4)Cl → RY*(1)N] that are associated with *E*^(2)^ of 4.88, 0.30 and 2.66 kcal mol^−1^, respectively. These insights collected using the MP2/def2-TZVPPD gas phase optimized geometry are consistent with that gained when the ion-pair geometry of the crystal was used. In this case, the strength of the hyperconjugative interaction is reduced because the interaction between the ions is longer ranged. Therefore, the strength of the hydrogen bond interactions was predicted to be of the van der Waals type, with an *E*^(2)^ for the delocalization [LP(Cl) → BD*(1)(H–N)] close to 0.1 kcal mol^−1^. By contrast, [LP(4)Cl → BD*(1) N–H], and [LP(4)Cl → RY*(1)N] were associated with an *E*^(2)^ of 0.70 and 1.07 kcal mol^−1^, respectively. These results unequivocally suggest that a single pnictogen bond is stronger than several hydrogen bonds formed between the ionic species Cl^−^ and NH_4_^+^, causing the formation of the ion pair.

**Figure 10:**
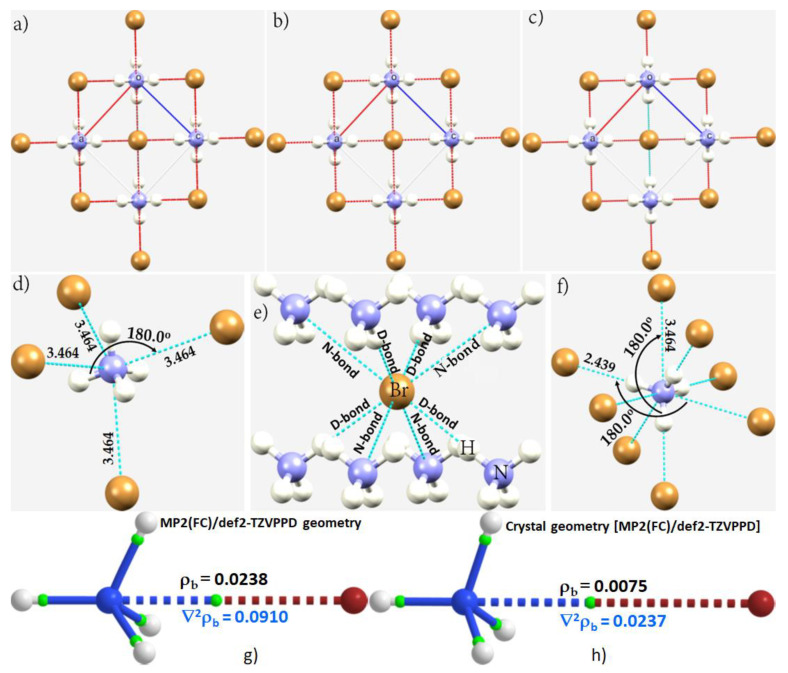
Three views of the crystal of tetradeuterioammonium bromide IV-LT, ND_4_Br (space group: *P*$\bar{4}$*3m*, ICSD ref. 26577 [103]) showing (**a**) combined D···Br deuterium bonds and N···Br pnictogen bonds, (**b**) N···Br pnictogen bonds, and (**c**) D···Br deuterium bonds. Illustration of the nature of the local nature of multi-furcated charge-assisted intermolecular bonding topologies: (**d**) ND_4_^+^ as four pnictogen bond donors; (**e**) Br^−^ as four D-bond and four N-bond acceptors; and (**f**) illustration of the nature of directionality for each of the four D and four N bond donors. Bond lengths and bond angles are in Å and degrees, respectively. The dotted lines represent inter-ionic interactions. Atom type is shown in (**e**). (**g**,**h**) MP2(FC)/def2-TZVPPD level QTAIM-based molecular graph for the ion-pair NH_4_Br, obtained on its MP2(FC)/def2-TZVPPD gas-phase geometry and as in the crystal, respectively; both these graphs show bond paths as sticks and dotted lines in atom color (N—blue; Br—dark-red; H—white grey), bond critical points as tiny spheres between atomic basins in green, and values (in *a.u*.) of charge density ρ_b_ and the Laplacian of the charge density ∇^2^ρ_b_ at the N···Br bond critical point.

The crystal structures of NH_4_Br and ND_4_Br (ICSD ref: 26577) [100] were reported some time ago. They occur as cubic and tetragonal crystals in various space groups, viz., *P*$\bar{4}$*3m*, *Pm*$\bar{3}$*m*, *Fm*$\bar{3}$*m* and *P_4_/nmm*, suggesting that these are phase change materials. The *P*$\bar{4}$*3m* crystal of ND_4_Br (ICSD ref: 26577 [100]) and the phase-I and -III (space groups *P*$\bar{4}$*3m* and *P_4_/nmm*, respectively) crystals of NH_4_Br (ICSD ref: 43300 [101] and 27724 [102], respectively) contain accurate information on the D/H atom positions. The low temperature cubic geometry of the crystal of ND_4_Br (ICSD ref: 26577) is shown in Figure 10a–c, illustrating the occurrence of charge-assisted D···Br deuterium bonding and N···Br pnictogen bonding interactions between the interacting ionic species. The local nature of the interaction between the ions, Figure 10d–f, is very similar to that observed for NH_4_Cl (Figure 9), although the D- and N-bond distances are slightly longer, as expected since the vdW radius of Br is larger than that of Cl (*r_vdW_*(Cl) = 1.82 Å and *r_vdW_*(Br) = 1.86 Å). For the phase-I low-temperature cubic crystal of NH_4_Br (ICSD ref. 43300 [101]), the corresponding H- and N-bonds are slightly longer (*r*(H···Br) = 2.518 Å and *r*(N···Br) = 3.516 Å) [102]. While the local topological pattern of H- and N-bonding interactions around the N atomic basin in NH_4_^+^ of the *P_4_/nmm* structure (ICSD ref: 27724) is very similar to that of the *P*$\bar{4}$*3m* structure (*r*(H···Br) = 2.509 Å and *r*(N···Br) = 3.482 Å) [102], the interacting angles deviate appreciably from linearity (∠N–H···Br = 167.6° and ∠H–N···Br = 175.3°). This suggests that both the hydrogen- and pnictogen-bonds in the tetragonal structure are distorted compared to those found in the cubic structure.

The gas-phase optimized N···Br inter-ionic distances of NH_4_Br were 2.881 and 2.851 Å with *ω*B97X-D/def2-TZVPPD and MP2(FC)/def2-TZVPPD, respectively, and were linear. These are substantially shorter than the corresponding distance of 3.516 Å found in the crystal geometry of the ion-pair (ICSD ref. 43300 [101]). The *ω*B97X-D/def2-TZVPPD, MP2(FC)/def2-TZVPPD and crystal geometries of the NH_4_^+^Br^−^ ion-pair were then separately used to calculate the complexation energy. The uncorrected [and BSSE corrected] complexation energy was −112.74 [−112.62], −114.65 [−113.06] and −98.45 [−97.76] kcal mol^−1^ for the corresponding geometry of the ion-pair, respectively; the latter value was calculated at MP2(FC)/def2-TZVPPD using the crystal geometry of the ion-pair. *E*_b_ = ½*V*_b_ gave values of −6.15, −6.96 and −1.35 kcal mol^−1^ as strength of the pnictogen bond in the *ω*B97X-D/def2-TZVPPD, MP2(FC)/def2-TZVPPD and crystal geometries of the ion pair, respectively; the latter calculated with MP2(FC)/def2-TZVPPD. QTAIM calculation gave *ρ*_b_ (and ∇^2^*ρ*_b_) values of 0.0224 (+0.0851), 0.0238 (+0.0910) and 0.0075 (+0.0237) *a.u.* at the corresponding geometries (and levels of theory), respectively. Figure 10g,h show that whereas the N···Br distance of separation in the gas phase and crystal geometries of the ion-pair differs substantially from each other, QTAIM identifies the pnictogen bonded interaction regardless of the nature of the geometry involved.

Similarly, as found for NH_4_Cl (see above), the RH/6-311G* level NBO’s second order analysis performed on the *ω*B97X-D/def2-TZVPPD level fully-relaxed geometry of the ion-pair NH_4_Br suggested the possible occurrence of both N···Br pnictogen bonding and H···Br hydrogen bonding. It provided evidence of three equivalent hydrogen bonds formed between the three H atom on the triangular face of NH_4_^+^ and the Br anion (see arrangement in Figure 10g,h). The N···Br pnictogen bond was the result of charge transfer delocalization between a lone-pair orbital on Br^−^ and the σ* anti-bonding orbital BD*(H–N). As such, *E*^(2)^ for delocalizations, LP(4)Br → BD*(1) N–H], and [LP(4)I → RY*(1)N], associated with the N···Br pnictogen bonding were 4.19 and 2.19 kcal mol^−1^, respectively. Similarly, each of three [LP(4)Br → BD*(1) H–N] charge transfer delocalizations associated with each of the three equivalent H···Br hydrogen bonds had *E*^(2)^ = 0.87 kcal mol^−1^. The delocalizations, LP(4)Br → BD*(1) N–H], and [LP(4)I → RY*(1)N], representing the pnictogen bond, were evident when the crystal geometry of the ion-pair was used, with *E*^(2)^ of 0.90 and 0.91 kcal mol^−1^, respectively, yet in this case the charge transfer energy of the H-bonded interactions was marginal.

**Figure 11:**
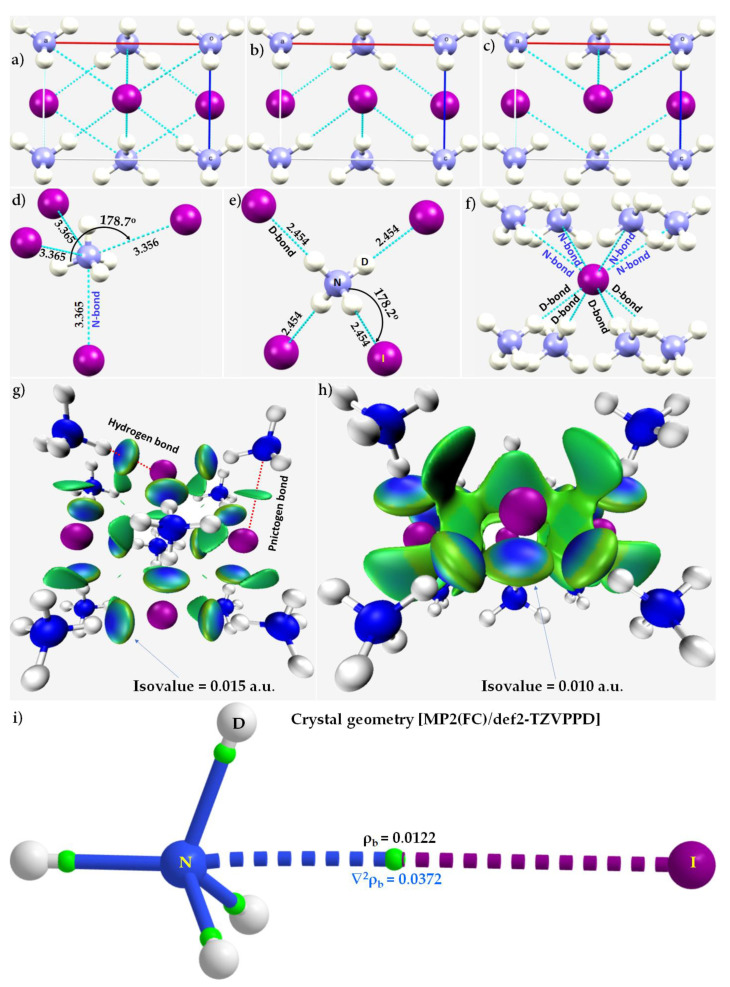
Three views of the crystal of ND_4_I (ICSD ref. 89094 [103]), showing (**a**) combined D···I deuterium bonds and N···I pnictogen bonds, (**b**) D···I deuterium bonds, and (**c**) N···I pnictogen bonds. Illustration of the nature of local nature of multi-furcated charge-assisted intermolecular bonding topologies: (**d**) N in ND_4_^+^ as four pnictogen bond donors; (**e**) ND_4_^+^ as four D bond donors; (**f**) I^−^ as four D-bond and four N-bond acceptors; (**g**,**h**) IGM-based isosurface plots between the interacting units as in the unit-cell of the crystal, with isovalues of 0.015 and 0.010 au, respectively. Selected bond lengths and bond angles are in Å and degrees, respectively. The dotted lines represent inter-ionic interactions. Atom type is shown in (**e**). (**i**) MP2(FC)/def2-TZVPPD level QTAIM-based molecular graph for the ion-pair ND_4_I, obtained on its geometry as in the crystal; it shows the bond paths as sticks and dotted lines in atom color, bond critical points as tiny spheres between atomic basins in green, and values (in *a.u.*) of charge density ρ_b_ and the Laplacian of the charge density ∇^2^ρ_b_ at the N···I bond critical point.

A number of structures of the H- and D-derivatives of ammonium iodide NH_4_I (*P*$\bar{4}$*3m*, *Pm*$\bar{3}$*m*, *Fm*$\bar{3}$*m* and *P_4_/nmm*), have also been deposited in the ICSD. Ammonium iodide undergoes a I–II–IV–V sequence of phase transitions at room temperature under pressure. The I–II phase transition occurs at *P_I–II_* = 0.5 kbar [104], while there is no reliable information on the II–IV transition pressure; it was speculated that *P_II–IV_* = 27 kbar on the basis of a subtle change observed in Raman spectra. Phase transition into the phase V was observed at *P* = 5 4 kbar, which was also observed in the Raman spectra [103]. The tetragonal crystal HP-IV (high pressure phase, 8600 MPa) contains accurate information on the D atom positions (ICSD ref. 89094) [103]. Balagurov et al. found that ND_4_I(V) has the same structure as the low-temperature phase ND_4_I(III)—a tetragonal structure with antiparallel ordering of ammonium ions, space group *P4/nmm* [100]. As in the *P_4_/nmm* structure of ND_4_Br, we found that the angles of interaction in the crystal structure of polymorphic phase IV of NH_4_I, Figure 11a–c, are slightly distorted from linearity in (∠N–D···I = 178.2° and ∠D–N···I = 178.7°). The local topological pattern of D- and N-bonding interactions around the ND_4_^+^ unit is shown in Figure 11d–f, indicating the presence of four equivalent deuterium bonds (as well as four equivalent pnictogen bonds), with *r*(D···I) = 2.454 Å and *r*(N···I) = 3.356 Å.

We note the feasibility of I···I contacts (*r*(I···I) = 3.964 Å) between the iodide anions in crystalline ND_4_I (ICSD ref: 89094). The feature is reminiscent of the Cl···Cl contacts (*r*(Cl···Cl) = 3.831 Å) observed in crystalline NH_4_Cl crystal (ICSD ref: 428519) [99].

The high pressure (9600 MPa) crystal of NH_4_I (space group *P4/nmm*; ICSD ref: 257453 [105]) shows that it is an effect of both hydrogen bonding and pnictogen bonding. The latter appears along the H–N bond extensions, and the former along N–H bond extensions. The iodide anion in the crystal lies approximately midway between two N atoms of two NH_4_^+^ moieties. For instance, *r*(H_3_N–H···I) = 3.352 Å and *r*(H_4_N···I) = 3.374 Å. The former is less directional than the latter (∠N–H···I = 179.6° and ∠H–N···I = 179.73°). The directional feature, together with the short intermolecular distance of separation between the atomic sites of opposite polarity, provides direct evidence of the formation of both hydrogen bonds and pnictogen bonds in the crystal. The I···I contact (*r*(I···I) = 3.912 Å) between the nearest iodide anions in crystalline NH_4_I is slightly shorter than those found in the ND_4_I. crystal (ICSD ref: 89094).

In the case of the crystal structures of ND_4_Br [100] and ND_4_Br [101], the Br···Br contacts were 4.000 and 4.118 Å, respectively. These are tertiary interactions, a forced consequence of the primary (H/D-bond) and secondary (N-bond) interactions in the ammonium halide crystals, and are weak and dispersion driven. This accords with the conclusions reached by Nelyubina et al. [99], who found the charge density at the H···Cl, N···Cl and Cl···Cl bcps were 0.13–0.19, 0.05–0.06 and 0.02–0.04 e Å^−3^, respectively, indicating that the stability of these bonding interactions in in the order H···Cl > N···Cl > Cl···Cl.

The IGM-δ*g* plots shown in Figure 11g,h confirm the presence of all these interactions. The presence of the H···I hydrogen bonds in NH_4_I is indicated by the thick circular volumes in greenish-blue, whereas the N···I pnictogen bonds manifest as triangle-like isosurface volumes in deep-green; both appear at isovalues of 0.010 and 0.015 *a.u.* The flat isosurface volumes in green corresponding to the I···I contacts were obtained with low isovalues because they are weak (Figure 11h). That N and I atomic basins in NH_4_I are bonded can be inferred from the MESP model of NH_4_^+^ (cf. Figure 8a).

The gas-phase relaxation of the geometry of the NH_4_^+^I^−^ ion-pair gave N···I inter-ionic distances of 3.103 and 3.055 Å with *ω*B97X-D/def2-TZVPPD and MP2(FC)/def2-TZVPPD, respectively. Indeed, these are very close to what was found in the geometry of the same ion-pair in the crystal [105] (*r*(H_4_N···I) = 3.374 Å). However, the gas-phase geometry was linear, whereas that in the crystal is quasi-linear. This is not very surprising since the H···I hydrogen bonds compete with pnictogen bonds in the crystal, and each ammonium cation is involved in forming eight non-bonded interactions with eight nearest-neighbor iodide ions (see Figure 11f for ND_4_^+^I^−^). The uncorrected [and BSSE corrected] complexation energy of the ion pair is calculated to be as large as −106.47 [−106.45], −109.17 [−107.33] and −101.72 [−100.46] kcal mol^−1^ with *ω*B97X-D/def2-TZVPPD and MP2(FC)/def2-TZVPPD and crystal geometries, respectively, the latter calculated on the ND_4_^+^I^−^ geometry at MP2(FC)/def2-TZVPPD. Our QTAIM calculation provides information about the N···I pnictogen bond in NH_4_^+^I^−^/ND_4_^+^I^−^, and the *ρ*_b_ (and ∇^2^*ρ*_b_) values were found to be 0.0191 (+0.0635), 0.0208 (+0.0699) and 0.0122 (+0.0372) *a.u.* at the corresponding geometries (and levels of theory), respectively, the latter obtained on the ND_4_^+^I^−^ geometry with MP2(FC)/def2-TZVPPD. Figure 11i shows the QTAIM-based molecular graph that provides evidence of the N···I pnictogen bonded interaction in ND_4_^+^I^−^, obtained using the crystal geometry of the ion-pair at MP2(FC)/def2-TZVPPD, and is reminiscent of the gas-phase NH_4_^+^I^−^ geometries as well (not shown). *E*_b_ = ½*V*_b_ gave values of −4.71, −5.52 and −2.57 kcal mol^−1^ as the strength of the N···I pnictogen bond in the *ω*B97X-D/def2-TZVPPD, MP2(FC)/def2-TZVPPD and crystal geometries of the ion pair, respectively, the latter calculated on the ND_4_^+^I^−^ geometry with MP2(FC)/def2-TZVPPD.

NBO’s second order analysis performed using the *ω*B97X-D/def2-TZVPPD geometry of the NH_4_^+^I^−^ ion pair suggested the occurrence of both H···I hydrogen bonding and N···I pnictogen bonding interactions between the interacting ions in NH_4_^+^I^−^. The former is several times weaker than the latter. This conclusion is based on the charge transfer delocalization energy; *E*^(2)^ corresponding to the delocalizations, LP(4)I → BD*(1) N–H], and [LP(4)I → RY*(1)N], associated with the N···I pnictogen bonding were 3.73 and 2.44 kcal mol^−1^, respectively. There were three delocalizations of the type, LP(4)I → BD*(1) H–N], associated with three equivalent H···I hydrogen bonds, each with an *E*^(2)^ of 1.1 kcal mol^−1^. Other lone-pairs on I are also involved in hyperconjugative interactions, explaining both the interaction types noted above, but *E*^(2)^ for them were < 0.75 kcal mol^−1^. When the crystal geometry of the ion-pair was used, *E*^(2)^ for two types of charge transfer delocalizations associated N···I pnictogen bonding were 2.20 and 1.52 kcal mol^−1^, respectively, and that associated with each of the three hydrogen bonds was 0.13 kcal mol^−1^.

### 3.6. The Crystal Structure of ([I_44_Pb_18_][CH_3_NH_3_]

Fateev and coworkers [106] reported that solvents such as DMSO, DMF and *γ*-butyrolactone (GBL) strongly influence the process of metal halide perovskite crystallization because of the formation of intermediate adducts with different structures and morphologies. Based on these observations, they were able to synthesize new adducts of lead-based halide perovskites and GBL with either an unusual cluster structure, [(MA)_8_(GBL)_x_][Pb_18_I_44_], or an adduct, [(MA)_2_(GBL)_2_][Pb_3_I_8_], similar to structures observed with DMF and DMSO.

**Figure 12:**
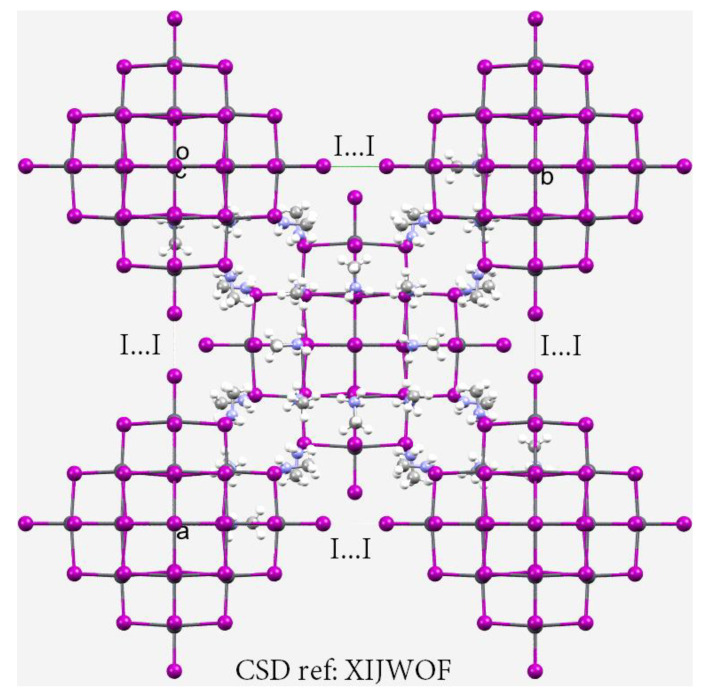
The ball-and-stick model of the unit-cell of the crystal structure of [I_44_Pb_18_]^8−^[CH_3_NH_3_]_8_ Atoms are shown as balls and are color coded: Iodine—purple; Carbon—gray; N—blue; H—white. The CSD ref. code is shown.

The structure of the [I_44_Pb_18_][CH_3_NH_3_]_8_ (CSD ref: XIJWOF [106], tetragonal crystal structure, space group *I_4_/m*) in shown in Figure 12. The size of the cluster anion [Pb_18_I_44_]^8−^ is 1−2 nm. The distance between the opposite vertices of [Pb_18_I_44_]^8−^ is 1.9 nm; the distance between the parallel planes is 1.1 nm [106]. The cluster [Pb_18_I_44_]^8−^ consists of 19 PbI_6_ edge-shared octahedra with a rock salt structure that arises from a transformation of the PbI_2_ lattice by an insertion of lead ions between the adjacent slabs.

Another iodoplumbate crystal structure, that of [I_44_Pb_88_][C_16_H_36_N]_8_, with tert-n-butylammonium cations (CSD ref: ZETLIT [107]), which contains the same inorganic framework, was reported in 1995. However, there is significant distortion in the organic cations, and there is uncertainty in their specific locations within the crystal structure.

**Figure 13:**
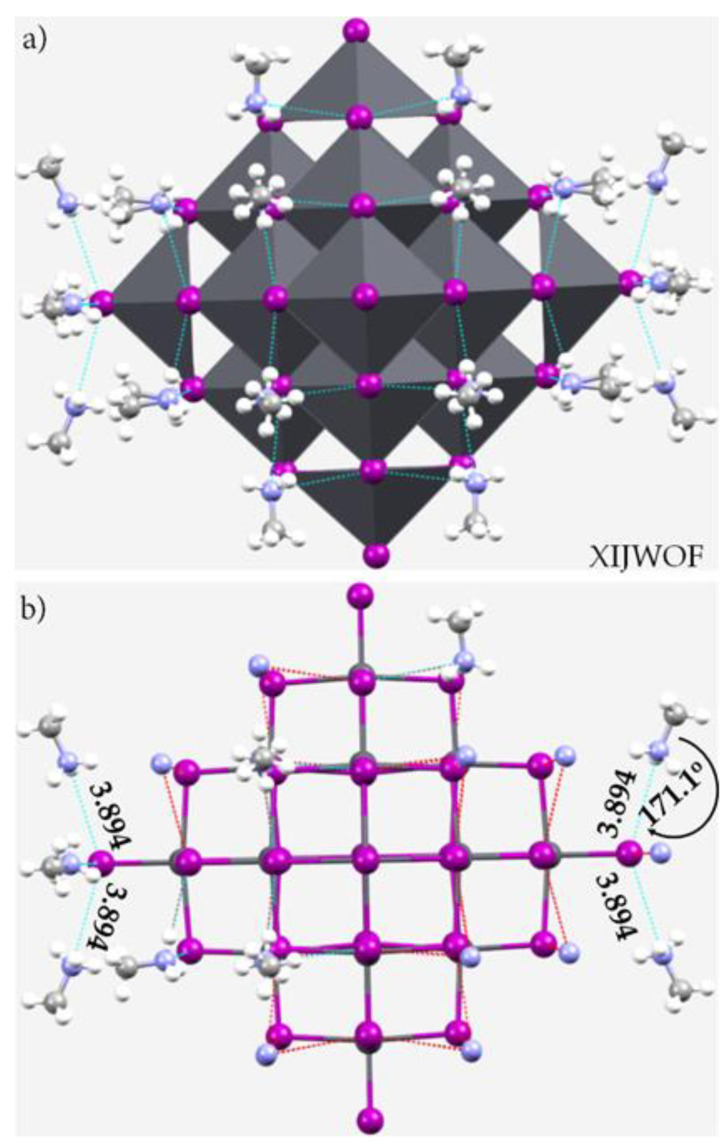
The spatial arrangement between the organic cations, [CH_3_NH_3_]^+^, and inorganic framework, [I_44_Pb_18_]^8−^, in the crystal structure of [I_44_Pb_18_]^8−^[CH_3_NH_3_]_8_, showing a number of quasi-linear N···I Type-IIa pnictogen bonds between the interacting units (dotted lines in red/cyan): (**a**) Polyhedral model and (**b**) ball-and-stick model of the inorganic framework. Selected bond lengths and bond angles are in Å and degrees, respectively.

Our analysis suggests that inorganic framework, [I_44_Pb_18_]^8−^, in [I_44_Pb_18_][CH_3_NH_3_] is stabilized by a number of intermolecular interactions: H···I hydrogen bonds, N···I pnictogen bonds, and I···I dihalogen bonds. The latter occur between the terminal I atoms of the [I_44_Pb_18_]^8−^ frameworks along the crystallographic *a* and *b* directions. The intermolecular distance between a pair of I atoms separating the inorganic frameworks is 4.081 Å and the interactions are linear (∠Pb–I···I = 180.0°). These I···I contacts are probably tertiary interactions, and are close to twice the sum of the vdW radius of the atoms, 4.08 Å. They arise as the consequence of primary (H···I hydrogen bonds) and secondary (N···I pnictogen bonds) interactions in the crystal lattice (Figure 13a,b).

Depending on the orientation of the organic cation, a metal-coordinated iodide can act as a receptor of at least three pnictogen bonds, with *r*(N···I) = 3.894 Å and ∠C–N···I = 171.1°. They are quasi-linear Type-IIa interactions since primary N–H···I hydrogen bonds formed by the ammonium fragment of the organic cation are simultaneously engaged attractively with the nearest-neighbor I^−^ sites of the inorganic cage structure. They appear in different flavors, with intermolecular bond distances varying between 2.80 and 3.50 Å.

### 3.7. Pnictogen Bond in 2D Functional Crystals of Metal Halide Perovskites: Derivatives of Ammonium as Pnictogen Bond Donors

Billing and Lemmerer [108] reported a series of layered crystal structures of the inorganic–organic perovskite-type hybrids, stable at room temperature, with general formula [(C*_n_*H_2*n*+1_NH_3_)_2_PbI_4_] (*n* = 12, 14, 16 and 18). Each of them displays two reversible phase transitions above room temperature, with phases labelled III, II and I. The transition from phase III to phase II was first-order and was related to a change in the conformation of the alkylammonium chains and a shift of the inorganic layers relative to each other.

**Figure 14:**
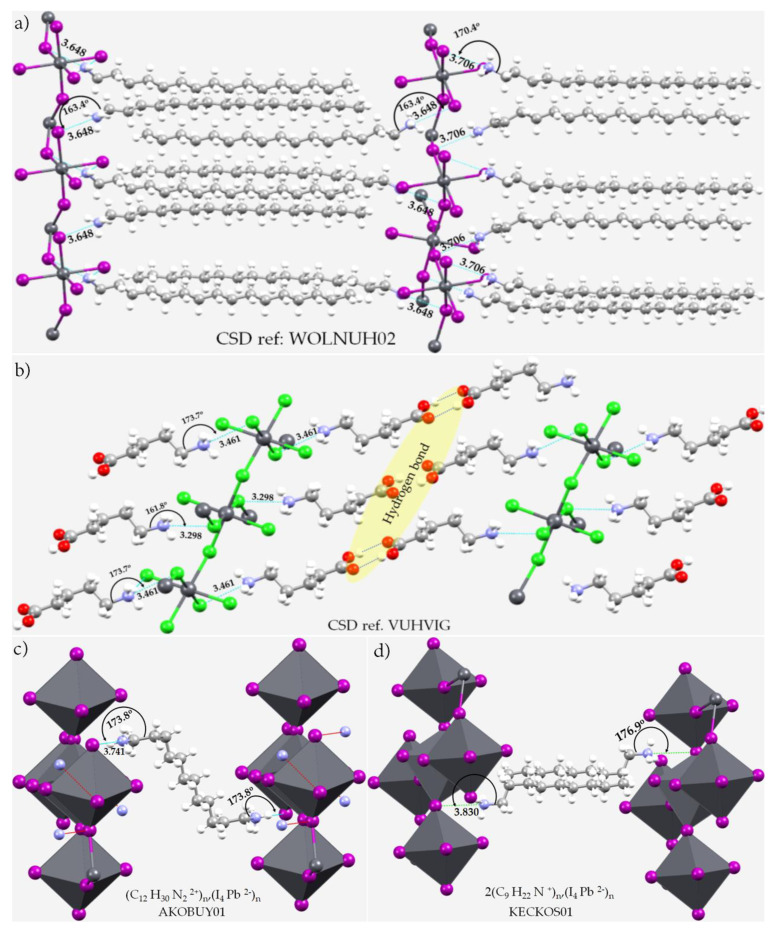
Mixed polyhedral (inorganic) and ball-and-stick (organic) models of the crystals of (**a**) [(CH_3_(CH_2_)_15_NH_3_)PbI_4_] [108]; (**b**) [C_5_H_12_NO_2_^+^]_4_[Pb_2_Cl_8_]_n;_ [109]; (**c**) [(H_3_N(CH_2_)_12_NH_3_)PbI_4_] [110]; and (**d**) [(CH_3_(CH_2_)_8_NH_3_)PbI_4_] [111]. Bond lengths and bond angles associated with selected intermolecular interactions are in Å and degrees, respectively. Space groups and CSD ref codes are shown for each case.

The crystal structure of one of these perovskites, with *n* = 16, is shown in Figure 14a. The organic cation chain works as an additive, or spacer or linker, connecting the PbI_4_^2−^ moieties through a number of N–H···I and C–H···I hydrogen bonds (*r*((N)H···I) = 2.818/2.766 Å and ∠N–H···I = 142.5°/165°; *r*((C)H···I) = 3.510 Å and ∠C–H···I = 139.5°). Because the terminal ammonium head is more acidic than the methyl head of the chain, it is engaged in a greater number of non-covalent interactions with a PbI_4_^−^ face compared to the methyl group which attracts a single iodide site of a given PbI_4_^2−^ layer. Other than these interactions, the N site in the ammonium terminal of the organic cation is also involved in the formation of charge-assisted N···I pnictogen bonding with an I site of the same PbI_4_^−^ face. It is longer, yet more directional than the N–H···I hydrogen bonds. They appear in two alternate flavors based on the nature of their spatial arrangement between the inorganic PbI_4_^2−^ layers. The intermolecular geometries associated with the contacts have *r*(N···I) ([∠C–N···I]) = 3.706 Å [170.4°] and 3.648 Å [163.4°] (cf. Figure 14a), featuring a Type-IIa topology of bonding. Because the pnictogen bond distances are either less than or close to the sum of vdW radii of N and I atomic basins, 3.70 Å, they could be categorized as secondary interactions, with the N–H···I hydrogen bonds the primary interactions. Nevertheless, the interactions are jointly responsible for stabilizing the crystal structure.

The pattern of intermolecular interactions in the system referred to above is similar to that in the crystal structure with odd *n*, as in [(C*_n_*H_2*n*+1_NH_3_)_2_PbI_4_] (*n* = 9), Figure 14d. In this case the N···I nitrogen bond formed by the terminal N atom of the ammonium moiety of CH_3_(CH_2_)_8_NH_3_^+^ is long-ranged and directional (Type-IIa), with *r*(N···I) and ∠C–N···I = 3.830 Å and 176.9°, respectively.

The crystal structure of a 2D metal bromide perovskite system, catena-(tetrakis(4-carboxybutylammonium) tetrakis(m-chloro)-tetrachloro-di-lead), [C_5_H_12_NO_2_^+^]_4_(Pb_2_Cl_8_]*_n_*, is shown in Figure 14b. The two mono-cationic linkers connecting the two neighboring inorganic layers are chain-like organic dications. Each dication, [(HOOC(CH_2_)_4_NH_3_)]^2+^, is linked to another dication through two non-equivalent C–H···O hydrogen bonds (*r*(H···O) 1.909 and 1.950 Å) (see the central region marked yellow in Figure 14b). The –NH_3_^+^ fragment of the linker is bonded to the Cl atoms of a face of a PbCl_6_ octahedron and involves N–H···Cl hydrogen bonds and C–N···Cl pnictogen bonds. The two types of pnictogen bonds have *r*(N···Cl) of 3.461 and 3.298 Å and are both quasi-linear Type-IIa interactions, with ∠C–N···Cl = 173.7° and 161.8°, respectively. The –NH_3_^+^ head is also involved in the formation of three or four non-equivalent *r*(H···Cl) hydrogen bonds. The shorter H-bond distances of three of them are at 2.582, 2.474 and 2.322 Å, with angles of approach of the electrophile of 138.6°, 152.9° and 175.7°, respectively. The first follows a Type-IIb pattern and the latter two follow a Type-IIa pattern of bonding. Similarly, the H sites of the other ammonium fragment involved in the other pnictogen bond (*r*(N···Cl) = 3.298 Å) is also involved in forming hydrogen bonds with distances of 2.474, 2.327 and 2.322 Å, corresponding to the angles of approach of the electrophile of 152.9°, 170.7° and 175.7°, respectively. Clearly, these attractive interactions interplay with each other to determine octahedral tilting of the PbCl_6_ octahedra of the inorganic frameworks of the metal halide perovskite. The octahedral tilting is a factor influencing the band gap and effective masses of charge carriers, a necessary perquisite for the functioning of perovskite compounds in photovoltaics and optoelectronics [26,112], and is driven by the competition between the N and H sites of the ammonium head in forming pnictogen and hydrogen bonding interactions.

**Figure 15:**
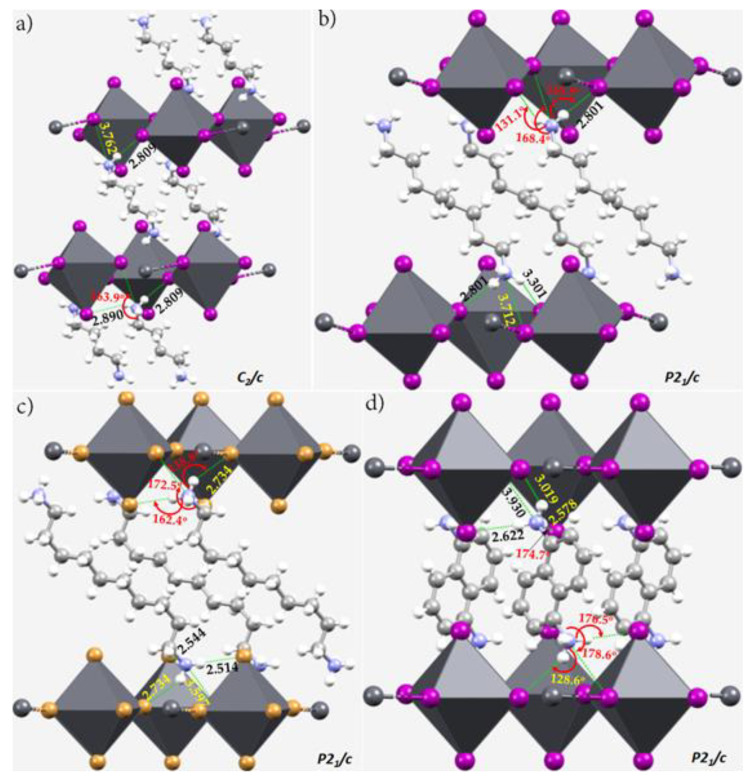
Mixed polyhedral (inorganic) and ball-and-stick (organic) models of the crystals of (**a**) (H_3_N(CH_2_)_4_NH_3_)PbI_4_] (**b**) (H_3_N(CH_2_)_8_NH_3_)PbI_4_] (**c**) (H_3_N(CH_2_)_10_NH_3_)PbBr_4_] and (**d**) (H_3_NC_10_H_6_NH_3_)PbI_4_, reported by Lemmerer and Billing [110]. Bond lengths and bond angles associated with selected intermolecular interactions are in Å and degrees, respectively. Space groups are shown for each case. Intermolecular interactions are marked as dotted lines in green.

Diammonium cations with the generic formula ^+^H_3_N–R–NH_3_^+^, where R group may be a linear-chain alkyl group, or an even-membered derivative of ethane, or a fused aromatic ring such as naphthalene, are well known. As spacers, they have been used as template moieties to stabilize the inorganic framework of a large number of inorganic-organic hybrid nanocomposites (cf. Figure 14c and Figure 15). Many two-dimensional layered perovskites with a RbAlF_4_ type structure and with linear alkyl chains between inorganic layers have been reported. They include, for example, [(H_3_N(CH_2_)_4_NH_3_)PbBr_4_], [(H_3_N(CH_2_)_4_NH_3_)PbI_4_], [(H_3_N(CH_2_)_8_NH_3_)PbI_4_], [(H_3_N(CH_2_)_10_NH_3_)PbBr_4_] and [(H_3_N(CH_2_)_12_NH_3_)PbI_4_]. Those where the R group contains a fused aromatic ring such as naphthalene, [(H_3_NC_10_H_6_NH_3_)PbI_4_], are also known. When the alkyl chain contains an odd number of –CH_2_ fragments, a 0D inorganic motif consisting of face-sharing PbI_6_ octahedra and isolated iodide anions have observed, as in [(H_3_N(CH_2_)_7_NH_3_)_4_Pb_3_I_12_]^2+^·2I^−^. 1D motifs such as [(H_3_NC_2_H_4_NH_3_)_4_PbBr_4_] and [(H_3_NC_6_H_4_CH_2_C_6_H_4_NH_3_)PbI_6_], which have edge-sharing twin-anionic chains of corner-sharing ribbons, have also been reported.

The structure of (NH_3_(CH_2_)_12_NH_3_)*_n_* PbI_4_·*n*(PbI_4_) (Figure 14c) has been reported by two different groups [110,113]. Both N–H···I hydrogen bonding and C–N···I pnictogen bonding are evident. The C–N···I pnictogen bonds are quasilinear (∠C–N···I = 173.8°), with *r*(N···I) of 3.741 Å. The bonding features in this system are similar to members of this family of compounds with smaller diamine cations; representative structures are shown in Figure 15a–c ((H_3_N(CH_2_)_4_NH_3_)PbI_4_, (H_3_N(CH_2_)_8_NH_3_)PbI_4_ and (H_3_N(CH_2_)_10_NH_3_)PbBr_4_, respectively). The pnictogen bond in the first two of these systems is far from linear. The *r*(N···Br) in (H_3_N(CH_2_)_10_NH_3_)PbBr_4_ is 3.597 Å, which is less than the sum of the vdW radii of Br and N, 3.52 Å. This is a notable feature of all the 2D iodide-based perovskites (cf. Figure 14 and Figure 15).

In cases where the templating agent is an are aromatic moiety, as in (H_3_NC_10_H_6_NH_3_)PbI_4_, shown in Figure 15d, the C–N···I pnictogen bonds are somewhat longer and linear. Both the ammonium fragments form equivalent pnictogen bonds, with ∠C–N···I = 178.6° and *r*(N···I) = 3.930 Å. The three shortest N–H···I hydrogen bonds identified for all cases are also illustrated in Figure 15d. They are formed by the H atoms of the ammonium fragment; two of them followed a Type-IIa pattern and the other a Type-IIb pattern.

**Figure 16:**
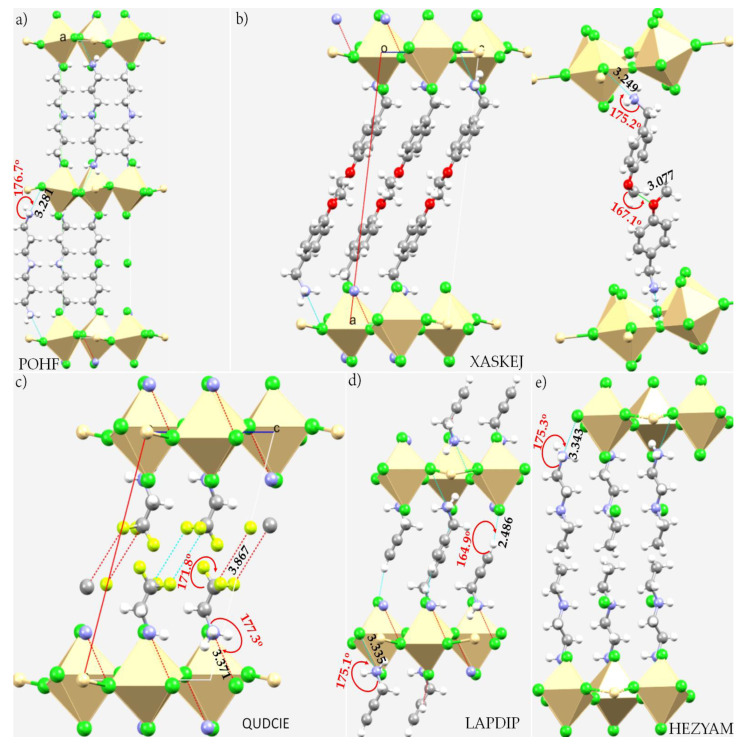
Polyhedral and ball-and-stick representations of (**a**) (C_6_H_20_N_3_)_2_[Cd_2_Cl_10_] (CSD ref. POHFEA [114]); (**b**) (OMeC_6_H_4_CH_2_NH_3_)_2n_·*n*(CdCl_4_) (CSD ref. XASKEJ [115]); (**c**) 2[CH_2_FCH_2_NH_3_]·[CdCl_4_] (CSD ref. QUDCIE [116]); (**d**) (CHCCH_2_NH_3_)_2*n*_·*n*(CdCl_4_) (CSD ref. LAPDIP [117]; and (**e**) (CH_3_CH_2_NH_2_CH_2_CH_2_NH_3_)_2*n*_·*n*(CdCl_4_),2*n*Cl (CSD ref. HEZYAM [118]). Intermolecular interactions are marked as dotted lines in cyan, the hanging contacts are marked as dotted lines in red. Selected bond distances and bond angles are in Å and degrees, respectively.

A similar system is [(NH_3_(CH_2_)_3_NH_2_(CH_2_)_3_NH_3_)_2_][Cd_2_Cl_10_], in which the organic cation carries a formal charge of +3 (Figure 16a). The 2D framework manifests through H···Cl hydrogen bonds and N···Cl pnictogen bonds; the former are formed by the terminal H atoms of the two ammonium groups and chloride ions. The latter are directional, with ∠C–N···Cl = 176.7° and *r*(N···Cl) = 3.281 Å. The bond lengths are markedly shorter than the sum of vdW radii, 3.48 Å, of Cl and N. The crystal consists of [CdCl_6_]^2−^ repeating units, with octahedrally coordinated Cd(III) ions linked bridging ligand Cl. This results in a 2D polymeric structure that runs along the *ac* crystallographic plane; Cl^−^ ions provide charge balance.

The crystal structures shown in Figure 16b–e are also two-dimensional polymeric structures, with different organic linkers. Whereas the H and N atoms of the ammonium fragment in these linkers are involved both in the formation of H···Cl hydrogen bonds and N···Cl pnictogen bonds, the chain-length of the organic spacers between the inorganic frameworks in Figure 16b,c,e is reinforced by attractive intermolecular interactions between a pair of two organic cations. The reinforcement is driven predominantly by charge-assisted C···O tetrel bonds and H···O hydrogen bonds between the two 4-(methoxyphenyl)methanaminium cations in Figure 16b, by C···F tetrel bonds between the 2,2,2-trifluoroethan-1-aminium cations in Figure 16c and by C···C/C···H interactions between the N-ethylethylenediammonium cations in Figure 16e. In the case of the crystal of (CH_3_CH_2_NH_2_CH_2_CH_2_NH_3_)_2*n*_·*n*(CdCl_4_), Figure 16e, the H site of the acetylenic fragment of the cation forms a strong H···Cl hydrogen bond with the inorganic framework and is strong enough to compete with the H···Cl hydrogen bonds and N···Cl pnictogen bonds formed by its other end. As noted above, perovskite-type layer compounds with general formula (R-NH_3_)_2_[MX_4_] or (NH_3_-(CH_2_)_n_-NH_3_)[MX_4_], (M = Cd, Mn, Cu, Pb …; X = Cl, Br, I; and R = a saturated or an unsaturated chain) have received considerable attention for some time as their crystal structures exhibit a quasi-two-dimensional arrangement with alternating inorganic (MX_6_)-organic (alkyl or alkylene chains) layers and undergo numerous structural phase transitions under temperature and/or pressure effects [119,120,121,122,123,124,125,126,127,128,129,130,131].

**Figure 17:**
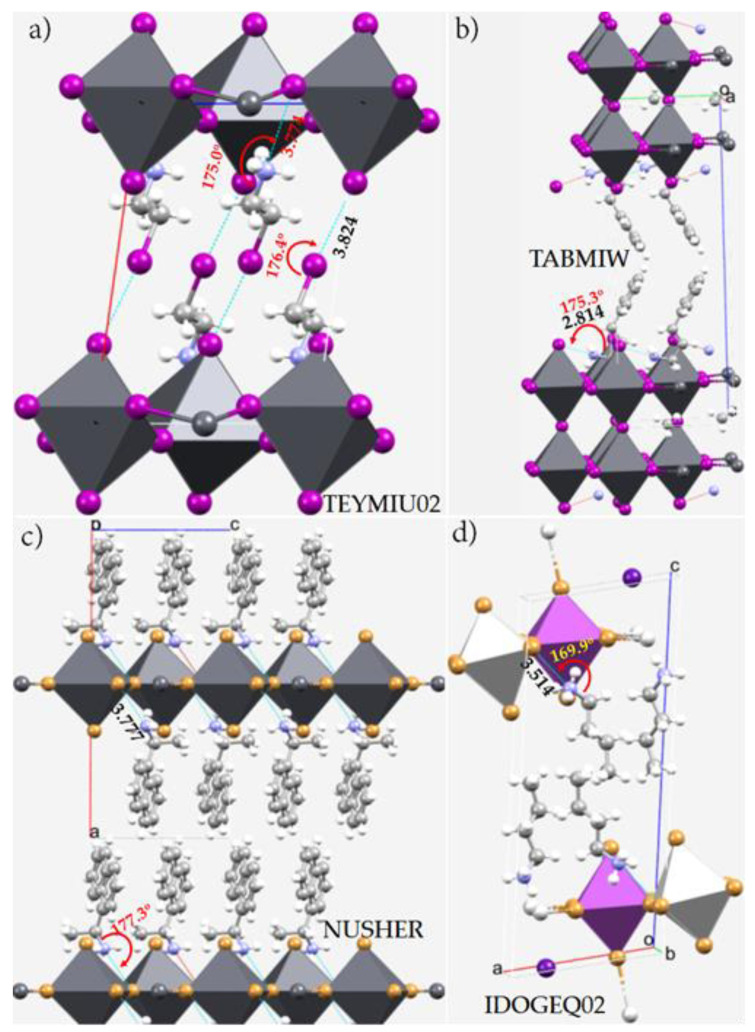
Polyhedral and ball-and-stick models of some crystals featuring highly directional N···X (X = Br, I) pnictogen bonding interactions. (**a**) (I−(CH_2_)_2_−NH_3_)_2_PbI_4_ [136]; (**b**) 2(C_9_H_12_N),0.5(C_2_H_6_),0.5(Pb_4_I_14_) [137]; (**c**) 2(C_12_H_14_N)(PbBr_4_)*_n_* [138]; and (**d**) (BA)_2_CsAgBiBr_7_ (BA = CH_3_(CH_2_)_3_NH_3_^+^) [139]. Intermolecular interactions are marked as dotted lines in cyan. Selected bond distances and bond angles are in Å and degrees, respectively. CSD references are shown in upper-case letters. The gray-white, purple and gray polyhedra represent the octahedral nature of Ag, Bi and Pb cations, respectively.

Another set of 2D metal halide perovskite systems in which H···N hydrogen bonds and directional pnictogen bonds formed between the organic and inorganic octahedra frameworks reinforce the layered structure, but in which they are not solely responsible for the crystalline structure, is shown in Figure 17. The pnictogen bonds appear as N···I contacts in Figure 17a,b, and as N···Br contacts in Figure 17c,d. The N···I and N···Br contacts, respectively, in Figure 17a,c,d are longer than the N···I contacts in Figure 17b, which are exceptionally short, suggesting a significant degree of covalency [132,133,134,135].

**Figure 18:**
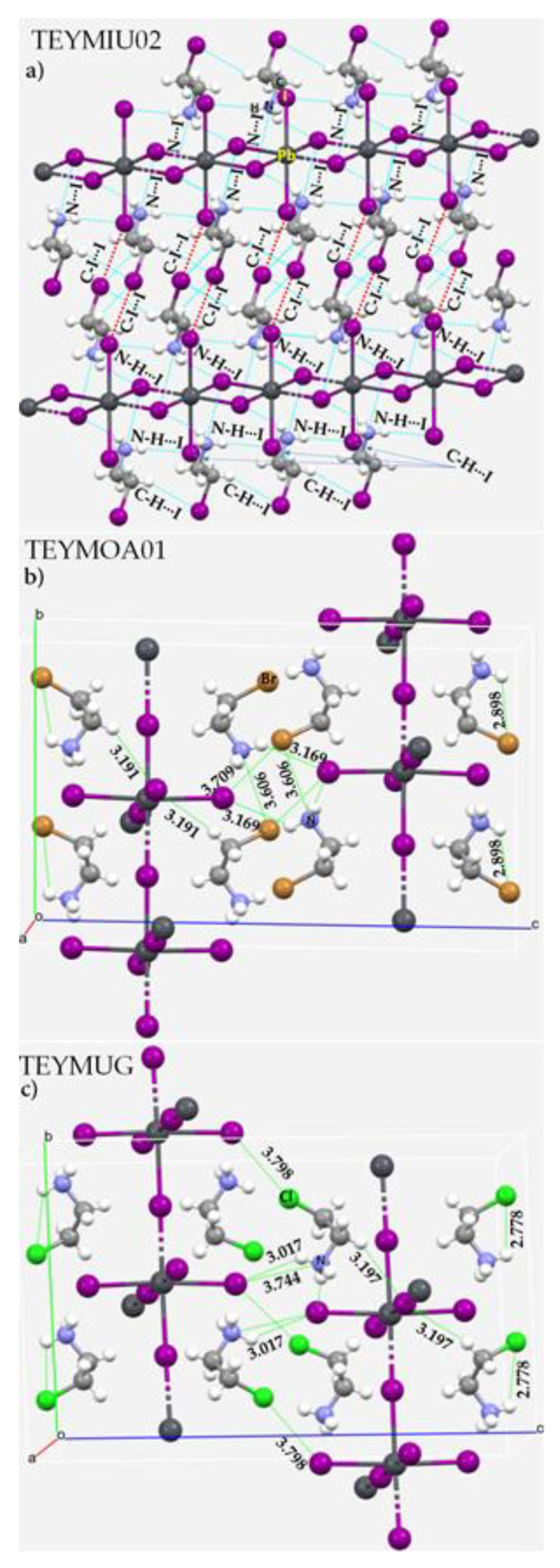
(**a**) Illustration of the nature of C-I···I halogen bonding, N···I pnictogen bonding, N–H···I and C–H···I hydrogen bonding interactions in the extended and unit-cell crystals of (I−(CH_2_)_2_−NH_3_)_2_PbI_4_. Shown in (**b**,**c**) are an illustration of the nature of Br···I and Cl···I halogen bonding, and N–H···I and C–H···I hydrogen bonding interactions in the unit-cell crystals of (Br−(CH_2_)_2_−NH_3_)_2_PbI_4_ and (Cl−(CH_2_)_2_−NH_3_)_2_PbI_4_, respectively. The intramolecular N–H···X′ hydrogen bonding interaction in the cis conformation of X′−(CH_2_)_2_−NH_3_^+^ (X′ = Br, Cl) is marked in (**b**,**c**). Labeling of selected atoms, as well as CSD reference codes in upper-case letters, is shown in all cases. Selected bond distances are in Å.

Other than the two types of interactions formed by the ammonium head mentioned above, we found that part of the stability of the system is driven by the attractive interaction of iodine in I−(CH_2_)_2_−NH_3_^+^ and the inorganic framework. As shown in Figure 17a and Figure 18a, these are nothing other than charge assisted C–I···I halogen bonds and C–H···I hydrogen bonds. The first are stronger than the second, inferred from the intermolecular distances and directionality features. In a study of some related structures, viz. (Cl-(CH_2_)_2_-NH_3_)_2_PbI_4_ (Figure 18b) and (Br-(CH_2_)_2_-NH_3_)_2_PbI_4_ (Figure 18c)) [140], it was suggested that the structural features of these systems are the result of halogen and hydrogen bonding at the organic−inorganic interface; the hydrogen bonds between equatorial iodine atoms of the perovskite layer and ammonium parts are absent.

Although the characterization of intermolecular bonding interactions in these systems was based purely on geometric factors, our close inspection not only revealed various other interactions, as mentioned above, but the conclusions of the authors may be incorrect since the equatorial iodine atoms of the perovskite layer and ammonium fragments are not free from hydrogen bonding (cf. Figure 18a–c). It was further argued that the red salts, such as (Cl−(CH_2_)_2_−NH_3_)_2_PbI_4_, display a reduced band gap (experimental: 2.2 eV; calculation: 1.9 eV) compared to (I−(CH_2_)_2_−NH_3_)_2_PbI_4_ in which tilting of the PbI_6_ octahedra and a larger bandgap (experimental: 2.45 eV; calculation: 2.3 eV) occurs [140]. This may not be correct since it is not a straightforward matter to determine whether the reduced band gap for the red-salt perovskites of the series has emerged from hydrogen bonding or halogen bonding interactions, or a combination of the two, at the organic−inorganic interface.

We did not find such directional N···I pnictogen bonds between the organic and inorganic counterparts in the undistorted lead iodide perovskite systems with X′−(CH_2_)_2_−NH_3_^+^ (X′ = Br, Cl) as spacer cations. It could be due to the organic spacer adopting a *cis* configuration as a result of its small size and reactivity of the lighter halogens. The geometry of these cations is accompanied by a charge-assisted intramolecular N–H···X′ hydrogen bonding interaction (*r*(H···X′) 2.898 Å in Br−(CH_2_)_2_−NH_3_^+^ and 2.778 Å in Cl−(CH_2_)_2_−NH_3_^+^), thus providing additional stability to the structure (cf. Figure 18b,c, respectively). It should be appreciated that the (Br−(CH_2_)_2_−NH_3_)_2_PbI_4_ and (Cl−(CH_2_)_2_−NH_3_)_2_PbI_4_ crystals are not free from C–H···I and N–H···I hydrogen bonds, as well as charge-assisted X′···I (X′ = Cl, Br) halogen bonds. The marked differences in intermolecular bonding features, as well as the inflexible nature of the conformational degree of freedom of the three bifunctional organic cations (adopts to a *trans* configuration in Figure 18a and to a *cis* configuration in Figure 18b,c), between the three crystal systems may explain why the degree of distortion of the perovskite layers, in terms of the degree of tilting of the octahedral related to the mean equatorial PbI_2_ plane, occurs in (I−(CH_2_)_2_−NH_3_)_2_PbI_4_, but not in (Cl−(CH_2_)_2_−NH_3_)_2_PbI_4_ and (Br−(CH_2_)_2_−NH_3_)_2_PbI_4_. From these structural features, it is clear that the bandgap reduction in the red-salts is a joint effect of the conformational freedom of the organic cation, the extent of various intermolecular interaction and the tilting of the octahedra.

**Figure 19:**
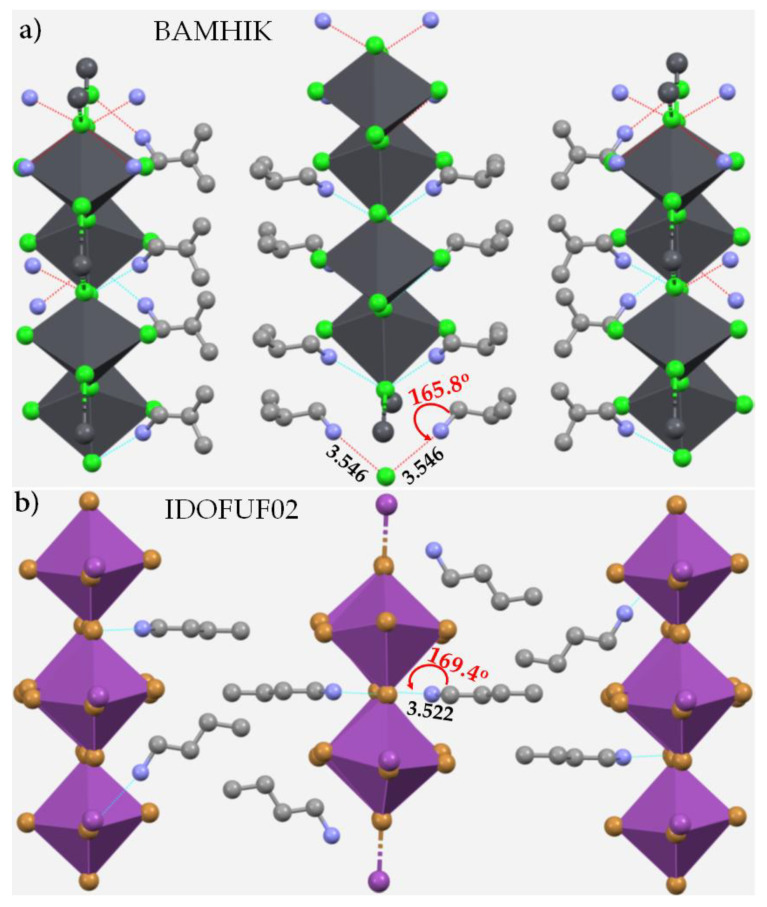
Polyhedral and ball-and-stick models of (**a**) [(CH_3_)_2_CHCH_2_NH_3_]_2_PbCl_4_ and (**b**) BA_2_Ag_0.5_Bi_0.5_Br_4_ (BA = CH_3_(CH_2_)_3_NH_3_^+^), displaying the nature of pnictogen-bonding interactions in the crystal. H-atoms of the organic cation are missing in the crystal structure shown in (**b**) and are not shown in (**a**) for clarity. The geometric aspects of the N···X (X = Cl, Br) pnictogen bonds are shown. The dark-gray and purple octahedra in (**a**,**b**) correspond to PbCl_6_ and BiBr_6_, respectively. Selected bond distances and bond angles are in Å and degrees, respectively. The CSD references are shown in each case. Intermolecular interactions are depicted as dotted lines in cyan and hanging contacts as dotted lines in red. The crystal structure of BA_2_Ag_0.5_Bi_0.5_Br_4_ deposited in the CSD does have a significant uncertainty in the position of Bi/Ag, which is not apparent in the polyhedral model.

The N···I pnictogen bonds in the crystal [2(C_9_H_12_N),0.5(C_2_H_6_),0.5(Pb_4_I_14_)] (referred to by the authors as (PPA)_2_(MA_0.5_FA_0.5_)Pb_2_I_7_ (PPA = 3-phenyl-2-propenammonium; MA = methylammonium; FA = formamidinium [137]), Figure 17b and Figure 19a, are short and directional, *r*(N···I) = 2.814 Å and ∠C–N···I = 175.3°, and are accompanied by non-linear N–H···I hydrogen bonds (*r*(H···I) = 2.6–2.7 Å; ∠N–H···I values in the range 89–120.0°).

**Figure 20:**
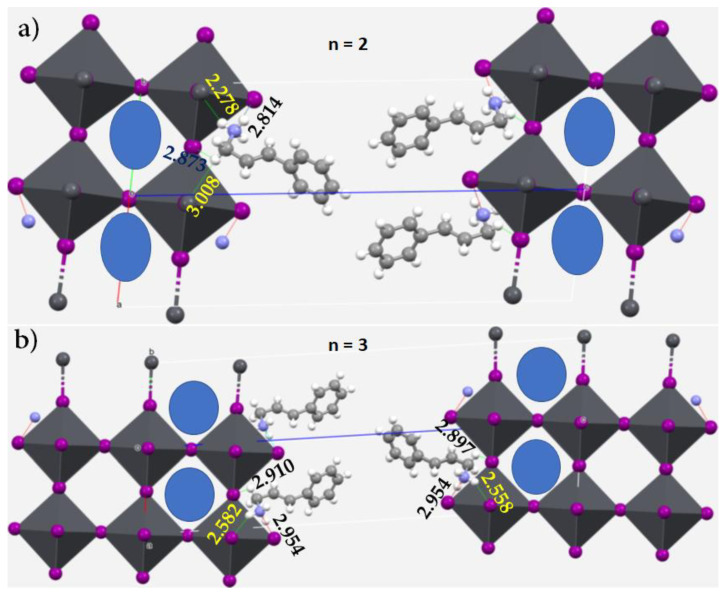
Polyhedral and ball-and-stick models of the crystal structure of (PPA)_2_(FA_0.5_MA_0.5_)*_n_*_−1_Pb*_n_*I_3*n*+1_ materials in 2D/3D: (**a**) *n* = 2 and (**b**) *n* = 3. Neither of these structures, as deposited in the CSD, show the correct orientation of the organic cations within the 3D perovskite cages, as provided in the supplementary material to the study [137], and hence we replaced them here by blue ellipses. The pnictogen bonded contacts are shown as dotted lines in cyan and the hanging contacts are in red. Selected intermolecular N···I, C–H···I and N–H···I bond distances and bond angles in Å and degrees, respectively.

In the case of (PPA)_2_(MA_0.5_FA_0.5_)_2_Pb_3_I_10_ (Figure 20b), the pnictogen bond is also short but less directional (∠C–N···I = 167.7° and (*r*(N···I) = 2.954 Å). The change in the geometric property of the pnictogen bond is not unexpected since the conjugated counter-cation spacer adjusts its position to maximize its attractive interaction with the iodides of the PbI_6_ octahedra of the inorganic frameworks. Both these crystals also show –HC–H···I type hydrogen bonds within a bond length range of 2.8–3.3 Å, which are significantly non-linear (∠C–H···I = 110–145°); they are weaker than the N–H···I hydrogen bonds.

Both crystals referred to above adopt a Ruddlesden–Popper (RP) type structure characterized by the general formula (A′)_2_(A)*_n_*_−1_Pb*_n_*X_3*n*+1_ (A′ = spacer cation, A = cage cation, and X = halide anion). In the reported crystal systems, 3-phenyl-2-propenammonium (PPA) with conjugated backbone plays the role of the spacer cation between the inorganic counterparts, and FA and MA as the inorganic cage cations (shown as blue ellipses in Figure 20), at low-temperature. This arrangement between the organic and inorganic moieties has led to the assembly of more efficient solar cells. The structure consists of 2D inorganic layers of corner sharing [PbI_6_]^4^^−^ octahedra which are separated by two layers of organic phenylpropylene ammonium cations (PPA^+^), and adjacent inorganic layers are shifted by ½ unit-cell length. The recorded trends in the absorption spectra suggested a progressively reduced bandgap as *n* increases because of less quantum and dielectric confinement, with optical bandgaps of approximately 1.94 and 1.75 eV for (PPA)_2_(FA_0.5_MA_0.5_)*_n_*_−1_Pb*_n_*I_3*n*+1_ when *n* = 2 and 3, respectively.

**Figure 21:**
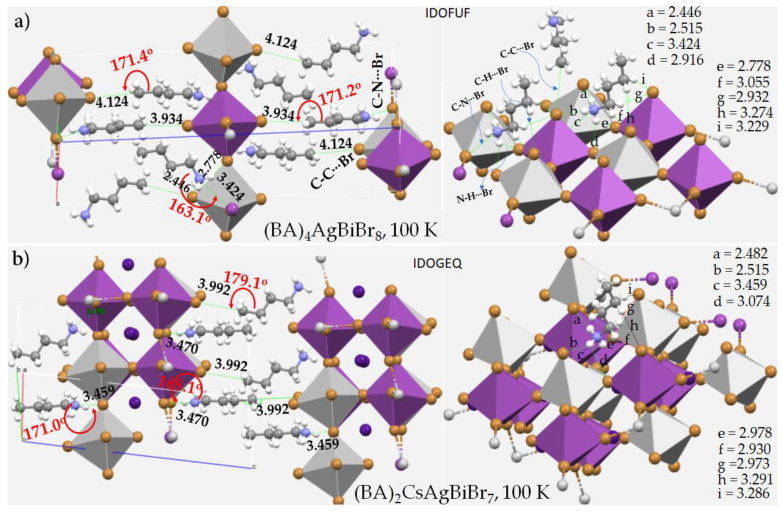
Polyhedral and ball-and-stick models of (**a**) (BA)_4_AgBiBr_8_ and (**b**) (BA)_2_CsAgBiBr_7_ (BA = CH_3_(CH_2_)_3_NH_3_^+^), displaying the nature of pnictogen- and tetrel-bonding interactions in the crystal. Shown on the right of (**a**,**b**) are the illustration of local modes of various interlayer intermolecular interactions (viz. hydrogen bonds, tetral bonds, and pnictogen bonds) formed between the inorganic and organic frameworks. Selected bond distances and bond angles are in Å and degrees, respectively. Some of the organic cations in (**b**) have been deleted for clarity. The CSD references are shown in each case in uppercase letters. The purple and white-gray octahedra correspond to BiBr_6_ and AgBr_6_ octahedra, respectively, whereas the dark-purple balls inside the cage in (**b**) represent the cesium cations.

The yellow plate-like crystals (BA)_4_AgBiBr_8_ and (BA)_2_CsAgBiBr_7_ (BA = *n*-butylammonium = CH_3_(CH_2_)_3_NH_3_^+^) are also members of the family A′_2_A*_n_*_−1_B*_n_*X_3*n*+1_ (*n* = 1 and 2). The structure of these organic-inorganic halide (001) perovskites were reported at 100 and 298 K [141]. However, the atomic positions of the organic cation, determined at the higher temperature, overlapped; hence this was not considered in this study. Although the overall geometry of each crystal lattices determined at the two different temperatures look similar, the local bonding environment is very different (Figure 21a,b). In the low temperature phase, the ammonium head of the organic cation is involved both in the formation of pnictogen bonding and hydrogen bonding, whereas the methyl head is involved in tetrel bonding. The pnictogen bond follows a Type-IIa pattern.

The non-covalent links formed by the nitrogen head in (BA)_2_CsAgBiBr_7_, 100 K, are not equivalent. For instance, the C–N···Br pnictogen bonds in the crystal have *r*(N···Br) = 3.459 Å (∠C–N···Br = 171.0°) and *r*(N···Br) = 3.470 Å (∠C–N···Br = 168.1°); the shortest N–H···Br hydrogen bonds accompanying the latter pnictogen bond are shown in Figure 21b (right). The shortest C–H···Br hydrogen bonds formed by the two methyl groups of the organic cation chain are longer and not equivalent (see Figure 21b, right).

In the case of (BA)_4_AgBiBr_8_, all three types of non-covalent links persist, yet the N···Br pnictogen bonds are equivalent and are shorter than that found in (BA)_2_CsAgBiBr_7_. By contrast, the C–C···Br tetrel bonds formed by the terminal methyl carbon of the organic cation repeat in an alternate fashion in (BA)_4_AgBiBr_8_, with *r*(C···Br) = 4.414 Å (∠C–C···Br = 171.4°) and *r*(C···Br) = 3.934 Å (∠C–C···Br = 171.2°) (Figure 21a, right); however, they are equivalent and linear in (BA)_2_CsAgBiBr_7_, with *r*(C···Br) = 3.992 Å (and ∠C–C···Br = 179.1°) (Figure 21b, right). Clearly, the competition between N···Br pnictogen bonding, C···Br carbon bonding, and N–H···Br and –(H)C–H···Br hydrogen bonding interactions in the crystal causes tilting of the corner-shared PbB_6_/AgBr_6_ octahedra in both the crystals. Because of the observed differences in the intermolecular bonding properties, the two perovskite systems in their powder and thin film forms display an onset of optical absorption at energies close to 2.7 eV ((BA)_2_CsAgBiBr_7_) and 2.77 eV ((BA)_4_AgBiBr_8_), highlighting the semiconducting nature of these systems in the solid state [141].

A very similar multiaxial layered halide double perovskite system, determined at 200, 298 and 400 K, was recently reported [139] (Figure 17d). It exhibits a fascinating ferroelectric feature with a Curie temperature of ~273 K and unique semiconducting nature. In the low temperature phase of the system the N···Br pnictogen bond is more directional (*r*(N···Br) = 3.514 Å; ∠C–N···Br = 169.9°) and is accompanied by N–H···Br and –(H)C–H···Br hydrogen bonding interactions. As above, the [(CH_3_)_2_CHCH_2_NH_3_]_2_PbCl_4_ lead halide 2D perovskite, Figure 19a, features an exceptionally large electrocaloric effect near room temperature [142]. It also exhibits a sharp first-order phase transition at 302 K, a superior spontaneous polarization > 4.8 *μ*C cm^−2^ and relatively small coercive field (<15 kV cm^−1^). The low temperature monoclinic phase (285 K) of the crystal is a result of a marriage between the [(CH_3_)_2_CHCH_2_NH_3_]_2_ and PbCl_4_ ions, accompanied by hydrogen and pnictogen bonding interactions. The latter interactions, N···Cl, are shown in Figure 19a. They follow a quasi-linear Type-IIa bonding topology, and the N–H···Cl hydrogen bonds are in the range 2.35–2.90 Å.

McClure et al. [143] have reported the synthesis, structure, and bandgaps of four layered halide double perovskites, BA_2_Cu_0.5_In_0.5_Cl_4_, BA_2_Ag_0.5_In_0.5_Cl_4_, BA_2_Ag_0.5_Sb_0.5_Cl_4_ and BA_2_Ag_0.5_Sb_0.5_Br_4_ (BA = CH_3_(CH_2_)_3_NH_3_^+^). They have an RP structure with *n* = 1. They revisited the crystal structure of BA_2_Ag_0.5_Bi_0.5_Br_4_ (Figure 19b) reported by Corner et al. [141] and also reported the crystal structure of BA_2_PbCl_4_. All these systems exhibited bandgaps in the range 2.65 eV (BA_2_Ag_0.5_Bi_0.5_Br_4_) to 4.27 eV (for BA_2_Ag_0.5_In_0.5_Cl_4_), with the bromide compounds featuring smaller bandgaps than the chloride compounds; this is expected since halide perovskites with heavier halogens generally exhibit smaller bandgaps [84]. The bandgaps of the bromide-containing RP phases, BA_2_Ag_0.5_M′_0.5_Br_4_ (M′ = Bi^3+^ and Sb^3+^), fall in the visible range, whereas those with chloride phases are wide bandgap materials (bandgap values between 3.2 and 4.3 eV); the former are shown to be suitable for visible light absorption. Similarly, the bandgaps of layered structures with composition BA_2_M_0.5_M′_0.5_X_4_ were observed to be between 0.5–0.8 eV larger than the analogous 3D Cs_2_MM′X_6_ cubic double perovskites, a consequence of dimensional reduction (3D → 2D), accompanied by distortions of the octahedral environment around the M/M′ ions, and octahedral tilting. In the 2D systems, the octahedral tilting is driven by various non-covalent interactions between the inorganic and organic layers, including pnictogen bonding, as noted in several crystal systems discussed above. For example, BA_2_Ag_0.5_Bi_0.5_Br_4_, Figure 19b, has equivalent N···Br pnictogen bonds, with *r*(N···Br) = 3.522 Å and ∠C–N···Br = 169.4°. They are directional, and together with hydrogen bonds, are clearly responsible for the rotation and tilting of the SbBr_6_ and AgBr_6_ octahedra in the structure.

Mei et al. [144] were probably the first to show that the protonated 5-amino valeric acid cation, HOOC(CH_2_)_4_NH_3_^+^, (5-AVA), can be used as an organic additive for the development of long-term stability of 2D perovskite solar cells. The cation has the advantage that it is not as toxic as organic diammonium cations, which are potentially deleterious to human health [109]. Subsequently, many such systems were reported, including, for instance, the quaternary representative 5-AVA_2_MA*_n_*_–1_Pb*_n_*Br_3*n*–1_ series of 2D bromide perovskite compounds [145] where *n* = 1–3. The value of *n* determines whether the compound comprises one, two or three layers. Similar 2D perovskite compounds containing chloride and iodide have also appeared. In the case of the iodide derivatives, *n* can reach values up to 6 or 7 [146]. However, the authors of this study were unable to synthesize pure samples of 5-AVA_2_MAPb_2_Br_7_ and 5-AVA_2_MA_2_Pb_3_Br_10_ [145]. The crystal structures of these 2D compounds demonstrate that they share some aspects of the structure of the cubic 3D perovskites with layers of corner-sharing PbBr_6_ octahedrons in (100) orientation. The structural stability of these crystals, such as other perovskite systems discussed above, arises from a significant number of hydrogen bonding interactions between the inorganic and organic components of the structure.

The PbX_6_ octahedra are rotated in these systems, and of course, the extent of this depends on the nature of halides in the inorganic frameworks and the intermolecular interactions involved. There is a monotonic decrease of the band gap from 3.02 eV through 2.80 eV to 2.70 eV as *n* increases from 1 to 2 and 3 in 5-AVA_2_MA*_n_*_–1_Pb*_n_*Br_3*n*–1_. The 5-AVA_2_PbBr_4_ perovskite system also displayed strong blue fluorescence (413 nm) with a remarkably small Stokes shift (47 nm). However, the stability of the systems with *n* = 2 and *n* = 3 was reduced compared to the simpler systems 5-AVA_2_PbBr_4_ and MAPbBr_3_–a result that was not in line with what was reported for the corresponding systems featuring iodide in the inorganic layer. This may be a consequence of the intermolecular chemical bonding between the organic and inorganic moieties and merits a detailed study.

**Figure 22:**
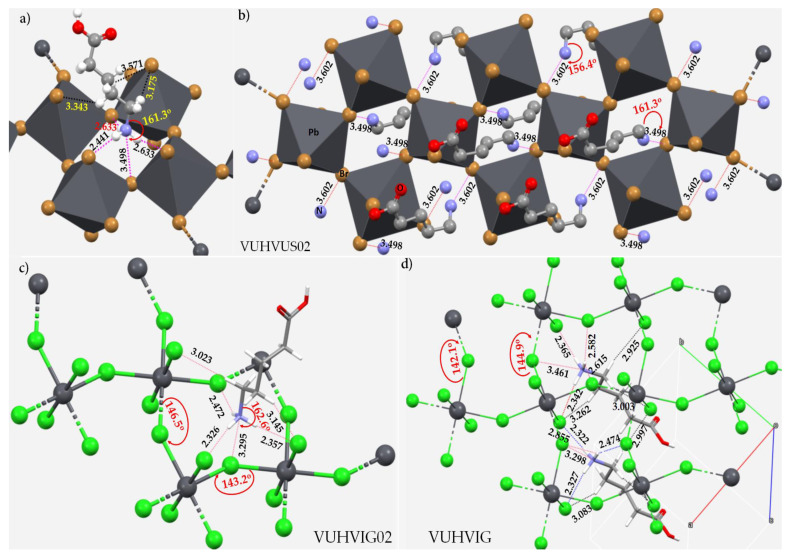
(**a**,**b**) The polyhedral ball-and-stick models of [HOOC(CH_2_)_4_NH_3_]_2_PbBr_4_ [147] showing (**a**) the nature of N–H···Br and C–H···Br hydrogen bonding and N···Br pnictogen bonding interactions, and (**b**) N···Br pnictogen bonding interactions (H atoms omitted), formed at the organic and inorganic interface. (**c**,**d**) The crystal structure of [HOOC(CH_2_)_4_NH_3_]_2_PbCl_4_ (CSD refs. VUHVIG02 [109] and VUHVIG [148]) showing the nature of N–H···Cl and C–H···Cl hydrogen bonding and N···Cl pnictogen bonding interactions formed at the organic and inorganic interface. Selected bond distances and bond angles are in Å and degrees, respectively.

The crystal structures of [HOOC(CH_2_)_4_NH_3_]_2_PbBr_4_ and [HOOC(CH_2_)_4_NH_3_]_2_PbCl_4_, showing the local nature of hydrogen- and pnictogen bonding interactions formed by the halides and the ammonium head of the cation, as well as between the halides and the two methyl groups the organic cation, are shown in Figure 22 [147]. The N–H···Br hydrogen bonds formed by ammonium moiety are significantly stronger than the –(H)C–H···Br hydrogen bonds formed by the methyl moieties (Figure 22a). There is a network of pnictogen bonds in the crystal that appear in two varieties, with *r*(N···Br) of 3.602 and 3.498 Å; the first is less directional than the second (∠C–N···Br = 156.4° vs. 161.3°, Figure 22b). The large deviation from linearity is because there is competition between all three H atoms of the ammonium moiety for formation of hydrogen bonds of appreciable strengths (based on the H···Br bond distances). The structure of [HOOC(CH_2_)_4_NH_3_]_2_PbCl_4_ features virtually similar intermolecular bonding at the interface region between the halides and the ammonium head (Figure 22c,d). The N–H···Cl and C–H···Cl hydrogen bonding and N···Cl pnictogen bonding interactions reported by Hillebrecht [148] are slightly different to those reported by Yang and coworkers [109]. Nevertheless, the overall nature of the local topology of non-covalent interactions is very such similar.

It was argued [109] that the intermolecular hydrogen bonding in AVA_2_PbCl_4_ causes a larger cation penetration depth that enables larger structural deformation than that observed in the reference system PDAPbCl_4_ (PDA = pentamethylenediammonium). The deformation of the PbCl_6_ octahedra leads to ultrabroadband emission in AVA_2_PbCl_4_, which has an enhanced photoluminescence quantum yield (2.83%) compared to PDAPbCl_4_ (0.4%). This led to the suggestion that the role played by intermolecular interactions results in the modulation of the photophysical properties of 2D perovskites, and this will be beneficial for the design of green perovskites for optoelectronic applications.

## 4. Pnictogen Bonding in 1D (One-Dimensional) Perovskite Systems

**Figure 23:**
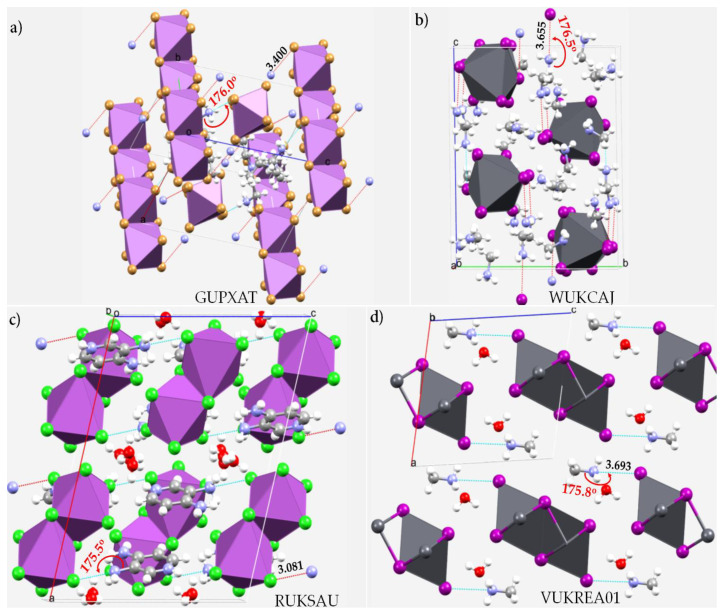
Ball-and-stick and polyhedral models of selected 1D structure. (**a**) bis(N^1^,N^1^-diethylethane-1,2-bis(aminium)) bis(μ-bromo)-octabromo-di-antimony (C_6_H_18_N_2_^2+^)_2_(Br_10_Sb_24_^−^) [154]; (**b**) pentakis(methylammonium) tris(μ-iodo)-hexakis(iodo)-di-lead bis(methylamine) (CH_6_N^+^)_5_(I_9_Pb_2_^5−^),2(CH_5_N) [155]; (**c**) bis(3-ammoniumyl-4-amino-pyridin-1-ium) bis(μ-chloro)-octachloro-di-bismuth dehydrate (C_5_H_9_N_3_^2+^)_2_(Bi_2_Cl_10_^4−^)2(H_2_O) [156,157]; (**d**) catena-[methylammonium bis(μ-iodo)-iodo-lead monohydrate (CH_6_N^+^)(I_3_Pb^−^)_n_,(H_2_O) [158]. Intermolecular contacts between bonded atomic basins are illustrated as dotted lines in cyan and hanging contacts in red. Selected bond distances and bond angles associated with the nitrogen-centered pnictogen bonds are in Å and degrees, respectively.

Many low-dimensional halide-based organic-inorganic hybrid perovskite compounds are known [149,150,151,152]. They sometimes adopt a step-like (SL-type) structure which is effectively a 1D quantum wire with chains of corner-sharing octahedra “insulated” by blocks of face-sharing octahedra [150]. For instance, among others, Hoffman et al. [150] have reported a new series of 1D SL-type structures with the general formula (PA)_2*m*+4_(MA)*_m_*_−2_Pb_2*m*+1_I_7*m*+4_ (*m* = 2, 3, 4), where the PbI_6_ octahedra connect in a corner- and face-sharing motif that exhibit resistivity trends that are dominated by ionic transport and no photoresponse. Yuan and coworkers [153] have reported the synthesis, crystal structure and photophysical properties of a 1D organic lead bromide perovskite, C_4_N_2_H_14_PbBr_4_. It consists of edge-sharing octahedral lead bromide chains [PbBr_4_^2−^]*_n_* that are surrounded by the C_4_N_2_H_14_^2+^ organic cations to form the bulk assembly of core-shell quantum wires. The 1D structure has enabled the authors to demonstrate the presence of strong quantum confinement with the formation of self-trapped excited states that give efficient bluish white-light emissions with photoluminescence quantum efficiencies of approximately 20% for the bulk single crystals and 12% for the microscale crystals. Shown in Figure 23 are a few illustrative 1D crystal structures that are stabilized by the joint involvement of hydrogen- and pnictogen-bonding interactions between the organic and inorganic frameworks.

The monoclinic hybrid compound [C_6_H_18_N_2_]_2_Sb_2_Br_10_ is monoclinically stable at room temperature, Figure 23a [154]. The photoluminescence measurements showed a strong emission line at 3.64 eV. The unaided-eye detectable blue luminescence emission came from the excitonic transition in the Sb_2_Br_10_ anions. The stability of the crystal structure arises from the intermolecular interaction between the bioctahedral [Sb_2_Br_10_]^4−^ dimers composed of two equivalent distorted octahedrons sharing one edge and the organic *N*,*N*-diethylethylendiammonium cation. Although the N–H⋯Br hydrogen bonds were shown to be the sole synthon for holding the crystal network, we found that C–N⋯Br pnictogen bonds in the crystal should not be overlooked. The bond distance associated with the latter are less than the sum of the vdW radii of Br and N and are directional (*r*(N···Br) = 3.400 Å; ∠C–N···Br = 176.0°).

The crystal structure of CH_3_NH_2_–CH_3_NH_3_PbI_3_, Figure 23b, was also considered to involve H···N hydrogen bonds between the organic moieties CH_3_NH_2_ and CH_3_NH_3_^+^) [155]. The CH_3_NH_2_···CH_3_NH_3_^+^ dimers in the CH_3_NH_2_–CH_3_NH_3_PbI_3_ intermediates were accompanied by 1D-PbI_3_^−^ chains (δ-phase). The weakly hydrogen-bonded CH_3_NH_2_ molecules could be easily released from the CH_3_NH_2_–CH_3_NH_3_PbI_3_ intermediates, probably causing rapid, spontaneous phase transition from 1D-PbI_3_^−^ (δ-phase) to 3D-PbI_3_^−^ (α-phase). From the crystal structure shown in Figure 23b we found that, in addition to H···N hydrogen bonds formed by the methylamine and methylammonium moieties, there are also weak C–N⋯I pnictogen bonds in the crystal that are directional. The weak aspect of these bonds is evident from the N···I intermolecular distances (*r*(N···I) = 3.655 Å; ∠C–N···I = 176.5°) that are less than the vdW radii sum of N and I atoms, 3.70 Å, and are longer than N···Br pnictogen bonds found in the [C_6_H_18_N_2_]_2_Sb_2_Br_10_ crystal (Figure 23a).

Senior and coworkers [157] have reported the crystal structures of six halobismuth(III) salts of a variety of substituted aminopyridinium cations. These crystals feature discrete mononuclear [BiCl_6_]^3−^ and binuclear [Bi_2_*X*_10_]^4−^ anions (X = Cl or Br), and polymeric *cis*-double-halo-bridged [Bi*_n_*X_4*n*_]*^n^*^−^ anionic chains (X = Br or I). One of these, bis(4-amino-3-ammoniopyridinium) di-μ-chlorido-bis[tetrachloridobismuth(III)] dihydrate, (C_5_H_9_N_3_)_2_[Bi_2_Cl_10_]·2H_2_O, incorporates discrete [Bi_2_Cl_10_]^4−^ anions. The Bi(III) centers in this and other crystals adopted a slightly distorted octahedral geometry and there was a correlation between the Bi—X bond lengths and the number of classic N—H···X hydrogen bonds that the X ligand accepts, with a greater number of interactions corresponding with slightly longer Bi—*X* distances. The supramolecular networks formed by classic N—H···X hydrogen bonds include ladders, bilayers and three-dimensional frameworks. As shown in Figure 23c, the geometric stability of (C_5_H_9_N_3_)_2_[Bi_2_Cl_10_]·2H_2_O not only emerges from the hydrogen bonds, but also C–N···Cl pnictogen bonds that are somewhat stronger than the C–N···Br and C–N···I found in the crystals shown Figure 23a,b, respectively.

The structure of catena-[methylammonium bis(μ-iodo)-iodo-lead monohydrate (CH_6_N^+^)(I_3_Pb^−^)_n_,(H_2_O) [158] is shown in Figure 23d. The crystal is a needle-shaped solvation intermediate similar to CH_3_NH3PbI_3_·DMF that has been recognized as the main cause for the incomplete coverage of the resultant thin film. By avoiding these intermediates, the films crystallized at the gas–solid interface offer several beneficial features for device performance including high surface coverage, small surface roughness, as well as controllable grain size. The stability of the intermediate is organized by N—H···I and N—H···O hydrogen bonds, and directional C—N···I pnictogen bonds.

**Figure 24:**
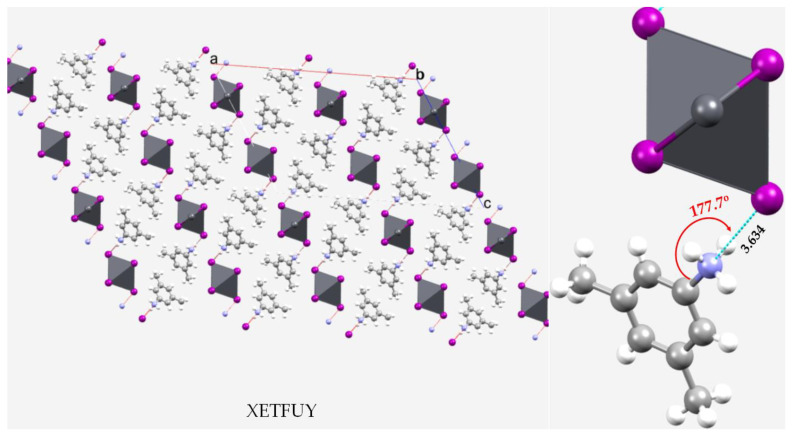
Ball-and-stick and polyhedral model of the 2 × 2 × 2 cell of the crystal of catena-(bis(3,5-dimethylanilinium) bis(μ_2_-iodo)-diiodo-lead(II)) [(C_8_H_12_N^+^)_2*n*_, (I_4_Pb^2−^)] [159]. Intermolecular contacts between bonded atomic basins are illustrated as dotted lines in cyan and hanging contacts in red. Selected bond distance and bond angle associated with the nitrogen-centered pnictogen bond is in Å and degrees, respectively. The CSD reference is shown in uppercase letters.

Another illustrative 1D perovskite system in 1D, [(C_8_H_12_N)_2_(PbI_4_)]*_n_*, that crystallizes as an organic-inorganic hybrid is shown in Figure 24 [159]. The structure consists of chains of [PbI_2_]^−^ units extending along the crystallographic *b* axis. In the asymmetric unit Pb is on a twofold axis and the two I atoms are in general positions. Each Pb atom is octahedrally coordinated to six I atoms, arranged as chains of edge-sharing octahedra. The N—H···I hydrogen-bonding interactions between the three H atoms of the ammonium group of the organic cation and the metal coordinate I atoms (*r*(H···I) 2.566, 2.835 and 3.147 Å); these are stronger than the C—H···I formed by a methyl group of the same cation and the coordinate I atoms (viz. *r*(H···I) 3.312 and 3.393 Å). There are also C—H···π and N—H···π hydrogen bonds between the ammonium and methyl groups of one organic cation and the arene ring system of another cation, as well as the formation of arene C– H···I hydrogen bonds and weakly bound Type-I I···I close contacts. Apart from these, the N site of the ammonium group is involved in forming pnictogen bonds with the nearest I sites of the inorganic framework, Figure 24 (right). These pnictogen bonds are quasi-linear, with (*r*(N···I) = 3.634 Å; ∠C–N···I = 177.7°.

## 5. Pnictogen Bonding in Zero-Dimensional (0D) Crystal Systems

**Figure 25:**
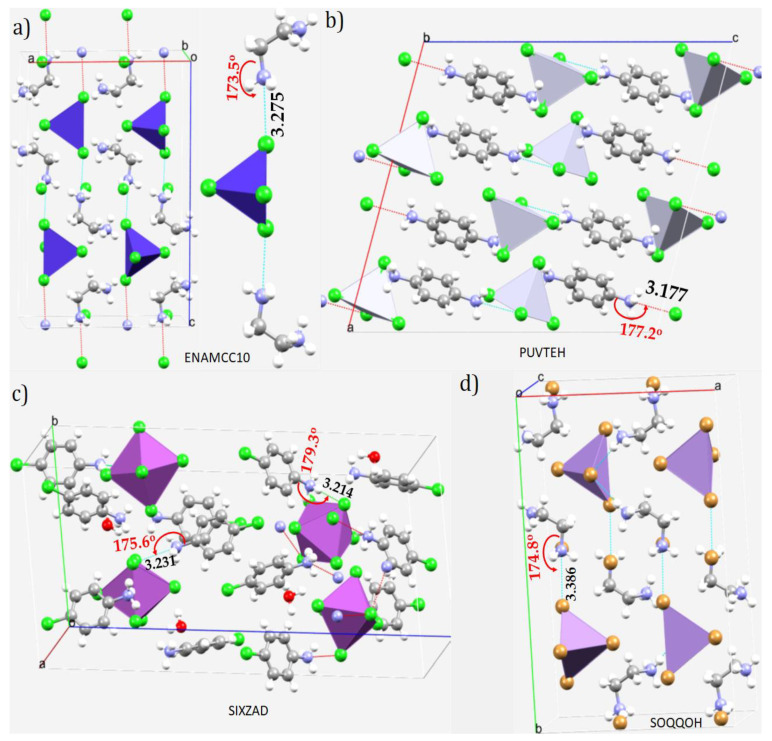
Ball-and-stick and polyhedral models of some selected 0D crystal structures. (**a**) bis(ethylenediammonium) tetrachloro-cobalt(II) bis(chloride), [CoCl_2_^2−^][C_2_H_10_N_2_^2+^_2_][Cl^−^]_2_ [163]; (**b**) 1,4-phenylenediammonium tetrachloro-mercury(II) (C_6_H_10_N_2_)^2+^(HgCl_4_^2−^) [164]; (**c**) tris(4-chloroanilinium) hexachloro-bismuth monohydrate [3(C_6_H_7_ClN^+^)_3_(BiCl_6_^3−^)(H_2_O) [165]; (**d**) bis(ethane-1,2-diaminium) bis(bromide) tetrabromo-manganese (C_2_H_10_N_2_^2+^)_2_,(MnBr^2−^)(Br^−^)_2_ [166]. Intermolecular contacts between bonded atomic basins are illustrated as dotted lines in cyan and hanging contacts in red. Selected bond distances and bond angles associated with the nitrogen-centered pnictogen bonds are in Å and degrees, respectively.

Several zero-dimensional (0D) crystal systems have been reported, wherein pnictogen bonding seemingly plays an important role in determining the stability of the crystals. For instance, (C_3_H_12_N_2_^2+^)(Cl^−^)_4_ (CSD ref. TAPSUY [160]), [2(C_2_H_10_N_2_^2+^)_2*n*_,(Cl_4_HgCl_4_^2−^)_n_,2(Cl^−^)_n_] (CSD ref: VUWVIS [161]) and [2(C_2_H_10_N_2_^2+^),2(Cl^−^),(HgCl_4_^2−^] (CSD ref: VUWVIS01 [162]) are examples of 0D crystal systems that involve nitrogen centered pnictogen bonding, among other non-covalent interactions. Figure 25 presents a few 0D crystal systems. These include [2(C_2_H_10_N_2_^2+^), Cl_4_ Co^2−^,2(Cl^−^)] (Figure 25a), [C_6_H_10_N_2_^2+^,Cl_4_Hg^2−^] (Figure 25b); [3(C_6_H_7_ClN^+^),BiCl_6_^3−^,H_2_O] (Figure 25c); and [2(C_2_H_10_N_2_^2+^),Br_4_Mn^2−^,2(Br^−^)] (Figure 25d). The presence of different organic cations are contributing to the the pentagonal pyramidal, tetrahedral, octahedral and tetrahedral geometries of the inorganic metal halides in these four systems, respectively.

The tetrahedra hybrid structure of (C_6_H_10_N_2_)^2+^(HgCl_4_^2−^), Figure 25b [164], shows two dimensional interwoven inorganic-organic layers bound through N-H···Cl hydrogen bonding interactions. Organic layers are accommodated between Cl···Cl halogen bonded inorganic layers with Cl···Cl distance = 3.628(3) Å and ∠Hg-Cl···Cl=157.8(9)°. The antiparallel zig-zag bilayer inorganic-organic pattern structures the material into a two-dimensional pattern in the *ac*-plane. The nearest centroid-centroid distance (3.86(4) Å) is indicative of π···π interactions and the nearest hydrogen to centroid interactions (2.82(3) Å) contribute to the stability of crystal structure through C-H···π interactions. The study did not consider the possibility of C–N···Cl pnictogen bonding in the crystal, which we identified to be directional and strong compared to that found in the crystal of [CoCl_2_^2−^][C_2_H_10_N_2_^2+^]_2_[Cl^−^]_2_, Figure 25a. For instance, the pnictogen bond in the crystal in Figure 25a (*r*(N···Cl) = 3.275 Å and ∠C–N···Cl = 173.5^o^) is weaker and less directional than that in Figure 25b (*r*(N···Cl) = 3.177 Å and ∠C–N···Cl = 177.2^o^).

(C_6_H_7_NCl)_3_(BiCl_6_)·H_2_O, Figure 25c [165], is a non-centrosymmetric organic-inorganic metallate complex and synthesized at room temperature. The cohesion between the organic and inorganic entities was suggested to be driven by N–H···Cl, N–H···O, C–H···O, C–H···Cl and O–H···Cl hydrogen bonding interactions between the 4-dichloroanilinium cations, the [BiCl_6_]^3−^ anions and water molecules, thus forming a pseudo-3D network. Our investigation of the crystal, however, revealed that pnictogen bonds also contribute to the cohesion of the system; this was overlooked in the original study. The pnictogen bonds appear at different intermolecular distances, Figure 25c; one of them has *r*(N···Cl) = 3.231 Å and ∠C–N···Cl = 175.6°, and the other has *r*(N···Cl) = 3.214 Å and ∠C–N···Cl = 179.3°.

The orthorhombic organic-inorganic compound (C_2_H_10_N_2_^2+^)_2_,(MnBr^2−^)(Br^−^)_2_, Figure 25d [166], is a 0D structure. The authors examined intermolecular interactions using single crystal X-ray diffraction and Hirshfeld surface analyses. It was found that this system exhibits an optical bandgap of 2.61 eV, a strong photoluminescence excitation line at 362 nm and a strong fluorescence at 537 nm and that the hybrid material features good thermal stability (decomposition temperature: 288–660 °C). Their structural analysis suggested that the inorganic anions are stabilized by [NH_3_CH_2_CH_2_NH_3_]^2+^ dications via an extensive number of H···Br hydrogen bonds. However, we also found the presence of N···Br pnictogen bonds in the crystal. These are formed between N of ammonium moiety and Br of four-coordinate MnBr_4_^2−^; they are quasi-linear (∠C–N···Br = 174.8°) and *r*(N···Br) = 3.386 Å).

**Figure 26:**
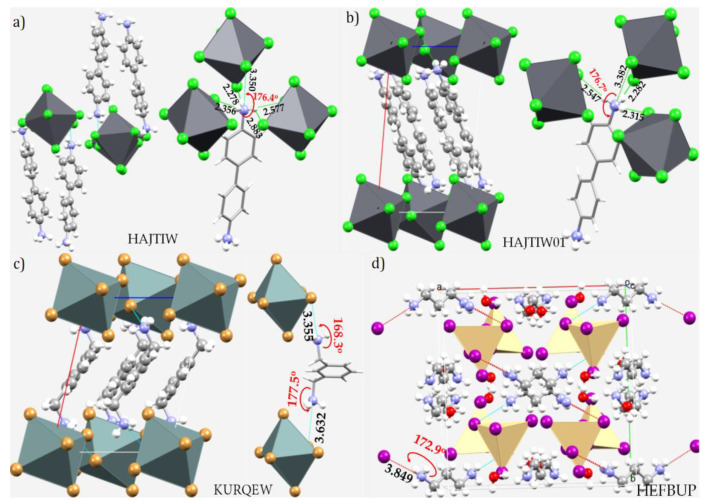
Ball-and-stick and polyhedral models of selected organic-inorganic hybrid metal halide perovskite and non-perovskite 0D structures. (**a**) bis(1,4-phenylenediammonium) hexachloro-lead(II) (C_12_H_14_N_2_^2+^)_2_(PbCl_6_^4−^) [167]; (**b**) the same compound the structure of which was reported by a different group [168]; (**c**) bis[(1,3-phenylene)dimethanaminium] hexabromo-tin (C_8_H_14_N_2_^2+^)_2_(SnBr_6_^4−^) [169]; (**d**) 1,3-propanediammonium tetraiodo-cadmium(II) dihydrate (C_3_H_12_N_2_^2+^)(CdI_4_^2−^)·2(H_2_O) [170]. Intermolecular contacts between bonded atomic basins are illustrated as dotted lines in cyan and hanging contacts in red. Selected bond distances and bond angles associated with the nitrogen-centered pnictogen bonds are in Å and degrees, respectively.

Shown in Figure 26a,b are the crystal structures of bis(1,4-phenylenediammonium) hexachloro-lead(II) [2(C_12_H_14_N_2_^2+^)_2_(PbCl_6_^4−^) reported by two different groups [167,168]. Whilst the nature of intermolecular interactions between the interacting organic and inorganic moieties are virtually similar, they differ in the intermolecular bond distances and bond angles. The PbCl_6_^4−^ octahedra are not linked to each other; this is therefore not a 1D crystal structure. The three H atoms of the ammonium head of the diammonium cation are involved in four N–H···Cl hydrogen bonds with bond distances of 2.278, 2.255, 2.356 and 2.883 Å (Figure 26a) and 2.282, 2.315, 2.547 and 2.885 Å (Figure 26b). The first three short bonds are Type-II interactions and the fourth is bent. The H atoms of the arene moiety placed close to the PbCl_6_^4−^ octahedra are also hydrogen bonded, with C–H···Cl in the range 2.8–3.0 Å. Apart from these, the N site in the ammonium heads of the diammonium cation are pnictogen bonded, with *r*(N···Cl) = 3.550 Å in Figure 26a and 3.382 Å in Figure 26b. These interactions are more directional (∠N–H···Cl close 176.4° or 176.6°) than the two types of short hydrogen bonds noted above (∠C–H···Cl varies between 141° and 172°).

A very similar topology of intermolecular bonding interactions is also present in the crystal of bis[(1,3-phenylene)dimethanaminium] hexabromo-tin (C_8_H_14_N_2_^2+^)_2_(SnBr_6_^4−^) [169], Figure 26c. The N···Br pnictogen bonds formed by the two ammonium heads are not symmetric; *r*(N···Br) = 3.632 Å and ∠C–H···Br = 177.5° for one pnictogen bond, and *r*(N···Br) = 3.355 Å and ∠C–H···Br = 168.3° for the other. This geometric feature is similar to those observed in the structures shown in Figure 26a,b (viz. *r*(N···Br) = 3.114 Å and ∠C–H···Br = 159.8° for one pnictogen bond and *r*(N···Cl) = 3.382 Å and ∠C–H···Cl = 176.7° for the other in Figure 26b). On the other hand, (C_3_H_12_N_2_^2+^)(CdI_4_^2−^)·2(H_2_O) [170], Figure 24, is a non-perovskite crystal and in this case, the N···I pnictogen bond is longer, with *r*(N···I) = 3.849 Å and ∠C–N···I = 172.9°).

## 6. Statistical Analysis of N-Centered Pnictogen Bond Distances and Angles in Crystals

Our search of the CSD for an R–(H/D)_3_N···X–R’ geometric motif with N···X in the range 2.6–4.0 Å and ∠R–N···X in the range 150–180° (X = any halogen derivative; R = any element; R’ = any metal) produced 1452 hits with 2337 close contacts. When the angular range was changed to 140–180° (160–180°) [170–180°] [[175–180°]] keeping the N···X intermolecular distance range unchanged, the number of hits became 1690 (967) [315] [[99]] with 3122 (1387) [404] [[109]] close contacts. When we constrained the N···X bond distance to the range 2.5–3.8 Å (or 2.5–3.7 Å) with ∠R–N···X in the range 175–180°, the number of hits were 88 (82) with 98 (90).

We only analyzed 82 hits from one of these searches to determine whether there were any false hits; only one false close contact was found. This was an N···Cl contact that appeared genuine in [Ru(NO)(NH_3_)_4_(SO_4_)](HSO_4_)·H_2_O (*r*(N···Cl) and [∠R–N···Cl] = 3.307 Å and [175.8°]) (CSD ref. AXOQAH [171]), but the N site in ammonia in this crystal system probably does not possess a positive region on its electrostatic surface with which to engage with the halogen (Cl) in a neighboring building block, causing an apparent N···Cl interaction. We did not categorize N···X (X = halogen) close contacts in the other crystal systems as false contacts since N in them was positive. For example, the crystal [C_4_H_16_Cl_2_N_6_Pt_2_].[ClO_4_] (CSD ref. LECRAN [172]) comprises two close contacts with *r*(N···Cl) [(∠R–N···Cl)] = 3.384 [175.0°] and 3.367 Å [177.4°]. In them, N in ammonia or ammonium has a positive region on its electrostatic surface, causing it to engage attractively with the Cl in a directional interaction.

**Figure 27:**
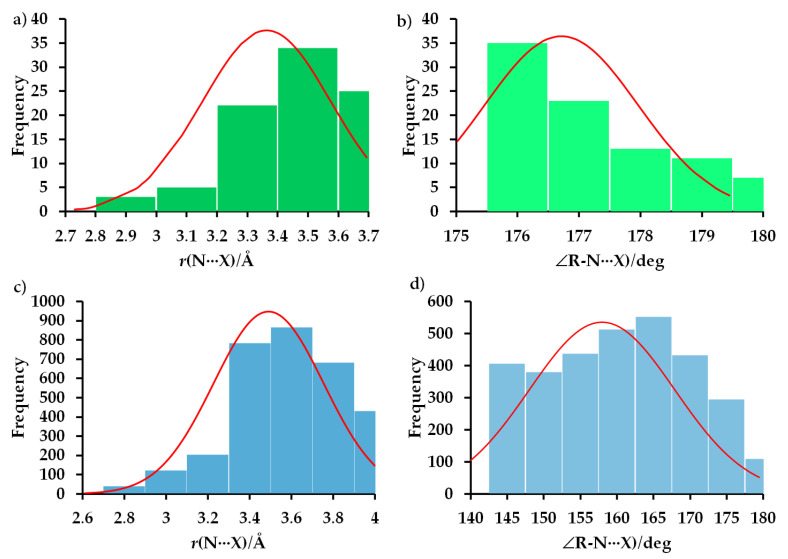
(**a**,**b**) Histograms illustrating the intermolecular distances *r*(N···X) and intermolecular bond angles ∠R–N···X (X = F, Cl, Br, I) for 81 hits (89 close contacts) found in a CSD search for the ranges 2.6–3.7 Å and 175–180°, respectively. Shown in (**c**,**d**) are the corresponding plots for 3122 geometric instances in 1690 crystals found with ranges of 2.6–4.0 Å and 140–180°, respectively. Potential false contacts in (**c**,**d**) have not been identified and omitted.

The histogram plots, together with the normal distribution of *r*(N···X) and ∠R–N···X found in the remaining 81 hits, comprising of 89 close contacts, are shown in Figure 27a,b. Figure 27c,d are from 1690 crystals with 3122 close contacts that emerged when N···X was in the range 2.6–4.0 Å and ∠R–N···X was in the range 140–180°. As can be inferred from the former, the formation of N-centered pnictogen bonding in crystals does not occur frequently in the bond distance and bond angle ranges of 2.6–3.7 Å and 175–180°, respectively. This suggests that highly directional nitrogen bonds are rare. This is understandable given that H atoms of the ammonium head are frequently involved in hydrogen bonds with nearest-neighbor halogens. From Figure 27c,d, it is clear that nitrogen bonds occur frequently in the distance range 3.2–3.8 Å, and angular range between 145–175°; these were originated especially when the electron density donors are the heavier halogens, Cl, Br and I.

We have also made an individual analysis of the N···X geometry for X in crystals obtained from a CSD search. We used the same geometry, R–(H/D)_3_N···X–R’, in our search, but the angle ∠R–N···X (X = I) was varied in the ranges 140–180° (150–180°) [160–180°] [[170–180°]] while keeping the N···X distance constrained to the range 2.6–4.0 Å. The four searches resulted in 398 (375) [271] [[82]] hits, with 711 (578) [362] [[96]] geometric instances. An inspection of the 82 crystals with 96 close contacts that emerged from the last search revealed that a great majority of them have perovskite structures in different dimensions, and five of them were a non-perovskites (viz. [2(C_6_H_18_N_3_^3+^)·(Cu_2_I_6_^4−^)·2(H_2_O)·2(I^−^)]; CSD ref. VEVVIE [173]). A non-perovskite crystal, ([C_5_H_10_I_2_N_4_OPt]·[C_3_H_7_NO]; CSD ref: VUPMOK) [174], was found to be a false hit since N in [C_5_H_10_I_2_N_4_OPt], does not have a positive region on its electrostatic surface. 

**Figure 28:**
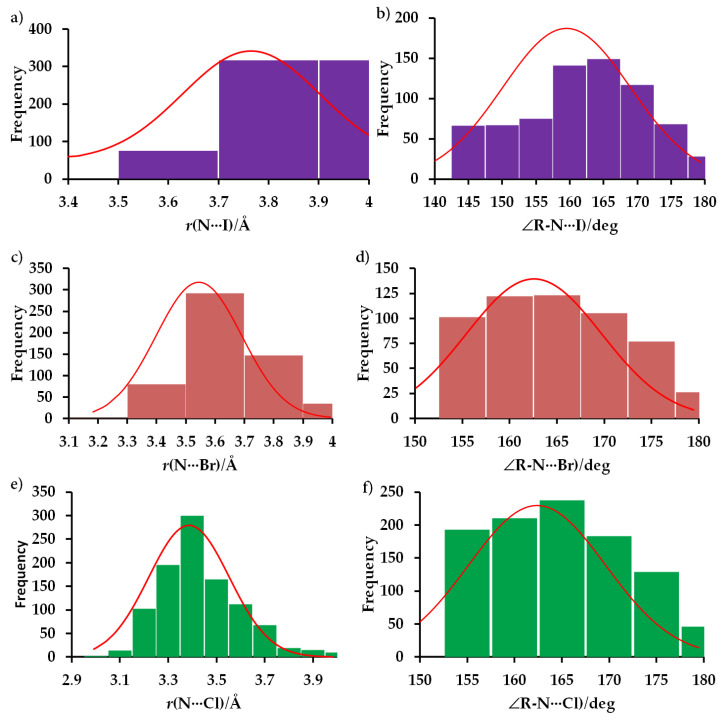
(**a**,**b**) Histograms illustrating the intermolecular distances *r*(N···I) and intermolecular bond angles ∠R–N···I for 81 hits (89 close contacts) found in a CSD search for the bond distance and bond angle ranges of 2.6–4.0 Å and 170–180°, respectively. Shown in (**c**,**d**) are the corresponding plots for 554 close instances from 309 hits for N···Br in the bond distance and bond angle ranges of 2.6–4.0 Å and 150–180°, respectively. Shown in (**e**,**f**) are the corresponding plots for 1000 close contacts from 658 hits for N···Cl in the bond distance and bond angle ranges of 2.6–4.0 Å and 150–180°, respectively. False contacts in (**a**,**b**) were removed, but not in (**c**–**f**). The bell curve in red in each case represents the normal distribution.

Figure 28a,b shows the normal distribution of *r*(N···I) and ∠R–N···I found in the remaining 81 hits comprising 89 geometric instances. From the former, it is apparent that N···I close contacts are extremely rare in the 2.6–3.2 Å range, and tend to occur in the range from 3.3 to 4.0 Å. The maximum of the normal distribution peaks around 3.77 Å. Similarly, the angle ∠R–N···I is populated largely around 160°, because the occurrence of pnictogen bond is largely influenced by hydrogen bonds formed by the ammonium head. There are a small number of N···I close contacts in the 175–180° range.

Our search of the CSD for the geometry R–(H/D)_3_N···X–R’ with ∠R–N···X (X = Br, Cl) in the range 140–180° and N···X in the range 2.6–4.0 Å gave 653 hits (691 close contacts) for N···Br, and 776 hits (1355 close contacts) for N···Cl. When we examined some of these crystals, we found that several of the N···X were bent (∠R–N···X between 140 and 150°). We then decided to search for the N···X close contacts that are within the angular range 150–180°. The resulting geometric instances are plotted in Figure 28c,d for N···Br close contacts, and in Figure 28e,f for N···Cl close contacts. The former consists of 554 geometric instances from 309 hits, and the latter of 1000 geometric instances from 658 hits. As can be seen, the nature of nature of the normal distribution of the angle ∠R–N···X is very similar when X = Br and X = Cl. A significant population occurs between 155° and 170° both for X = Cl and X = Br. However, the normal distribution curve associated with X = Cl shows that N···Cl close contacts are largely in the range 3.2–3.6 Å with a peak around 3.4 Å. This was not the case when X = Br, where the N···Br close contacts are largely in the 3.4–3.8 Å with a peak around 3.5 Å. These results suggest that N···X intermolecular distance can be longer than the sum of the vdW radii of N and X in many crystals.

## 7. Conclusions

This overview focused on several crystal structures, formed between various organic and inorganic moieties, to reveal the frequent existence of nitrogen-centered charge-assisted pnictogen bonding interactions; these have been largely overlooked in the metal halide perovskite literature. Since our motivation was to draw attention to the occurrence of this type of interaction in crystals, we examined its chemical manifestations. Our review of crystals structures deposited in the CSD suggests that N in ammonium derivatives is a potential pnictogen bond donor, and is, in part at least, responsible for the stability and functionality of many organic-inorganic hybrid perovskite systems, and hence could be exploited in the design of novel materials for photovoltaics, photocatalysis and other optoelectronics.

Our analysis suggests that the ammonium parts of mono-, bi- and multi-functional organic cations interact attractively with halogen atoms of the inorganic counterparts of the metal halide perovskites in two ways. Firstly, the N atom forms directional pnictogen bonding interactions with the bridging halogen sites of the MX_6_*^n^*^−^ octahedra of the inorganic counterpart. Secondly, the H atoms form hydrogen bonding interactions either with the bridging halogens and/or terminal halogen atoms, depending on the architectures of the organic cations and inorganic anions. Based on the spatial orientation of the organic cation, an additive, a single H-atom in the ammonium fragment could sustain an attractive engagement with at least two halogen atoms of the inorganic octahedra, thus forming a bi- or tri-furcated interaction bonding topology.

In cases where one of the terminal sites of the organic cation is –H, –X, or –CH_3_, the formation of charge-assisted H···X hydrogen bonding, or X′···X halogen bonding, or H···X/C···X hydrogen/tetrel bonding interactions between the organic cation and inorganic metal halide anion framework is a likely outcome. These, together with the attractive engagements driven by the ammonium part of the organic cation, act as forces that hold the layered inorganic framework together in low dimension and hence provide stability to the overall geometry of the organic-inorganic metal halide compounds.

The tilting of the MX_6_ octahedra of the inorganic framework in halide-based perovskites is often found to be driven by the competition between N···X pnictogen bonding, H···X hydrogen bonding and together with, when they occur, other non-covalent interactions. This attribute is shown through several illustrative crystal systems, including, for instance, orthorhombic MAPbX_3_ (X = Cl, Br, I) systems in 3D (Figure 4), and those shown in Figure 14, Figure 15 and Figure 16, Figure 18, Figure 19 and Figure 20 in 2D. What specific role does the nitrogen-centered pnictogen bonds play in determining the optoelectronic properties of the functional perovskite materials is not revealed in this study, and hence should be the topic of any future studies. We believe that the nitrogen-centered pnictogen bonding interactions revealed in this study will be beneficial to those are interested in the development of novel organic-inorganic hybrid halide-based perovskite materials and beyond. 

While the complexation energy of several illustrative simplified ion-pairs examined in this study was very large owning to the dominance of coulombic interactions involved, the empirical relationship known between potential energy density at the bcp and interaction energy allowed us to suggest that pnictogen bonds explored in these models were locally of weak-to-medium strength, and hence their contribution to the crystal stability should be not overlooked. However, it should be kept in mind that the intermolecular interaction energies estimated from intermolecular bond critical point properties of electron densities could be inherently unreliable, since they typically underestimate, but sometimes overestimate, more reliable values. Conclusions drawn regarding the energetic importance of specific intermolecular interactions based on this empirical relationship can be misleading, especially in the context of crystal packing and crystal engineering [175]. These interactions appeared persistently regardless of whether the geometry of the ion-pair in the crystal, or optimized with MP2 and DFT, was used for QTAIM analysis; their feasibility in the ion-pair was also confirmed by NBO’s second order analysis. Although it is not straightforward to quantify the energetic strength of each of the interaction types in each crystal system illustrated in this study, even with detailed computational studies, the simple model systems presented at the beginning of this overview may be considered for investigation using state of the art energy decomposition procedures, such as Interacting Quantum Atom Model, to quantify the strength of each of the interactions involved. In addition, charge density approaches (viz. ELF, RDG and QTAIM) may also be applicable to most of the crystal systems to explore the nature of various interlayer interactions that occur in the low dimensional crystal systems. These approaches allow one to visualize the nature of electron density localization, delocalization and depletion, among others. Future studies will address whether such computational and theoretical attempts would enable us to better understand the pnictogen-centered non-covalent bonding environments responsible not only for the stability of the crystals, but also their functionality in areas as diverse as optoelectronics.

## Figures and Tables

**Table 1:** Uncorrected and BSSE corrected Self-Consistent Fieldinteraction energies (*E*_int_ and *E*_int_(BSSE), respectively) of H_3_C-H_3_N···I and H_3_N-H_3_C···I ion-pairs, obtained on the fixed crystal geometry, as well as that on the MP2(FC)/TZVPPD and *ω*B97X-D/TZVPPD optimized geometries. Values are in kcal mol^−1 a^.

System	(*ω*B97X-D)_crystal geometry_		(*ω*B97X-D)_optimized geometry_		[MP2(FC)]_crystal geometry_		[MP2(FC)]_optimized geometry_	
	*E*_int_	*E*_int_(BSSE)	*E*_int_	*E*_int_(BSSE)	*E*_int_	*E*_int_(BSSE)	*E*_int_	*E*_int_(BSSE)
H_3_C-H_3_N···I	−94.25	−94.24	−102.1	−102.08	−95.68	−94.42	−105.31	−103.16
H_3_N-H_3_C···I	−70.21	−70.19	−76.1	−76.07	−71.16	−70.26	−78.77	−76.91

^a^ (*ω*B97X-D)_crystal geometry_ and (*ω*B97X-D)_optimized geometry_ refer to the crystal and *ω*B97X-D geometries of the ion-pairs used for the calculation of binding energies with *ω*B97X-D. Similarly, [MP2(FC)]_crystal geometry_ and [MP2(FC)]_optimized geometry_ refer to the crystal and MP2(FC) geometries of the ion-pairs used for the calculation of binding energies with MP2(FC).

**Table 2:** Comparison of total and dissected energy SAPT0 components with the SCF based interaction energies of H_3_C-H_3_N···I and H_3_N-H_3_C···I ion-pairs, obtained on the fixed crystal geometry, and the MP2(FC)/TZVPPD and *ω*B97X-D/TZVPPD optimized geometries. Values are in kcal mol^−1^.

Geometry (Complex Type)	*E*_els_	*E*_exch_	*E*_ind_	*E*_dis_	*E*_int_(SAPT0)	*E*_int_(SCF)(BSSE) ^a^
Crystal geometry (H_3_C-H_3_N···I)	−91.00	9.32	−8.87	−2.87	−93.44	−94.24
Crystal geometry (H_3_N-H_3_C···I)	−67.97	4.47	−4.4	−2.01	−69.92	−70.19
*ω*B97X-D (H_3_C-H_3_N···I)	−108.8	26.84	−14.12	−5.93	−102.00	−102.08
*ω*B97X-D (H_3_N-H_3_C···I)	−82.91	19.92	−8.51	−5.25	−76.76	−76.07
MP2(FC)(H_3_C-H_3_N···I)	−111.74	31.04	−14.95	−6.55	−102.2	−105.31
MP2(FC)(H_3_N-H_3_C···I)	−85.94	24.51	−9.46	−6.03	−76.93	−78.77

^a^ See Table 1 for *E*_int_ and *E*_int_(BSSE) values.

**Table 3:** RHF/6-311G* level second-order perturbative estimates of ‘donor- acceptor’ (bond-antibond) interaction energies, *E*^(2)^ (in kcal mol^−1^), in the NBO basis, obtained for H_3_C-H_3_N···I and H_3_N-H_3_C···I using their MP2(FC)/TZVPPD and *ω*B97X-D/TZVPPD level optimized geometries.

Interaction Type	Donor NBO		Acceptor NBO	*E*^(2)^[MP2(FC] Geometry]	*E*^(2)^[*ω*B97X-D Geometry]
(C)N···I	LP(4) I	→	RY*(1)N	2.86	2.66
(C)N···I	LP(4) I	→	BD*(1)N-C	4.51	3.99
N-H···I	LP(2)I	→	BD*(1)N-H	0.62	0.51
N-H···I	LP(2)I	→	BD*(1)N-H	0.62	0.51
N-H···I	LP(3)I	→	BD*(1)N-H	0.83	0.68
N-H···I	LP(4) I	→	BD*(1)N-H	1.14	0.94
N-H···I	LP(4) I	→	BD*(1)N-H	1.14	0.94
N-H···I	LP(4) I	→	BD*(1)N-H	1.14	0.94
(N)C···I	LP(4)I	→	RY*(1)(C)	2.58	2.26
(N)C···I	LP(1)I	→	BD*(1)C-N	0.56	0.41
(N)C···I	LP(4)I	→	BD*(C-N)	10.22	8.14
N-H···I	LP(2)I	→	BD*(1)C-H	0.12	0.08
N-H···I	LP(2)I	→	BD*(1)C-H	0.12	0.08
N-H···I	LP(3)I	→	BD*(1)C-H	0.12	0.11

## Data Availability

Not applicable.
